# Obesity-induced inflammation exacerbates clonal hematopoiesis

**DOI:** 10.1172/JCI163968

**Published:** 2023-06-01

**Authors:** Santhosh Kumar Pasupuleti, Baskar Ramdas, Sarah S. Burns, Lakshmi Reddy Palam, Rahul Kanumuri, Ramesh Kumar, Taruni Reddy Pandhiri, Utpal P. Dave, Nanda Kumar Yellapu, Xinyu Zhou, Chi Zhang, George E. Sandusky, Zhi Yu, Michael C. Honigberg, Alexander G. Bick, Gabriel K. Griffin, Abhishek Niroula, Benjamin L. Ebert, Sophie Paczesny, Pradeep Natarajan, Reuben Kapur

**Affiliations:** 1 Herman B Wells Center for Pediatric Research, Department of Pediatrics and; 2 Division of Hematology/Oncology, Department of Medicine, Indiana University School of Medicine, Indianapolis, Indiana, USA.; 3 Department of Biostatistics and Data Science, University of Kansas Medical Center, Kansas City, Kansas, USA.; 4 Department of Medical and Molecular Genetics and; 5 Department of Pathology and Laboratory Medicine, Indiana University School of Medicine, Indianapolis, Indiana, USA.; 6 Cardiovascular Research Center, Massachusetts General Hospital, Boston, Massachusetts, USA.; 7 Program in Medical and Population Genetics and the Cardiovascular Disease Initiative, Broad Institute of Harvard and MIT, Cambridge, Massachusetts, USA.; 8 Division of Genetic Medicine, Department of Medicine, Vanderbilt University Medical Center, Nashville, Tennessee, USA.; 9 Department of Pathology, Brigham and Women’s Hospital, Boston, Massachusetts, USA.; 10 Epigenomics Program, Broad Institute of Harvard and MIT, Cambridge, Massachusetts, USA.; 11 Department of Medicine, Brigham and Women’s Hospital, Harvard Medical School, Boston, Massachusetts, USA.; 12 Department of Microbiology and Immunology, Medical University of South Carolina, Charlestown, South Carolina, USA.; 13 Department of Medicine, Harvard Medical School, Boston, Massachusetts, USA.; 14 Department of Microbiology and Immunology, Indiana University School of Medicine, Indianapolis, Indiana, USA.

**Keywords:** Hematology, Inflammation, Cancer, Hematopoietic stem cells

## Abstract

Characterized by the accumulation of somatic mutations in blood cell lineages, clonal hematopoiesis of indeterminate potential (CHIP) is frequent in aging and involves the expansion of mutated hematopoietic stem and progenitor cells (HSC/Ps) that leads to an increased risk of hematologic malignancy. However, the risk factors that contribute to CHIP-associated clonal hematopoiesis (CH) are poorly understood. Obesity induces a proinflammatory state and fatty bone marrow (FBM), which may influence CHIP-associated pathologies. We analyzed exome sequencing and clinical data for 47,466 individuals with validated CHIP in the UK Biobank. CHIP was present in 5.8% of the study population and was associated with a significant increase in the waist-to-hip ratio (WHR). Mouse models of obesity and CHIP driven by heterozygosity of *Tet2*, *Dnmt3a*, *Asxl1*, and *Jak2* resulted in exacerbated expansion of mutant HSC/Ps due in part to excessive inflammation. Our results show that obesity is highly associated with CHIP and that a proinflammatory state could potentiate the progression of CHIP to more significant hematologic neoplasia. The calcium channel blockers nifedipine and SKF-96365, either alone or in combination with metformin, MCC950, or anakinra (IL-1 receptor antagonist), suppressed the growth of mutant CHIP cells and partially restored normal hematopoiesis. Targeting CHIP-mutant cells with these drugs could be a potential therapeutic approach to treat CH and its associated abnormalities in individuals with obesity.

## Graphical abstract

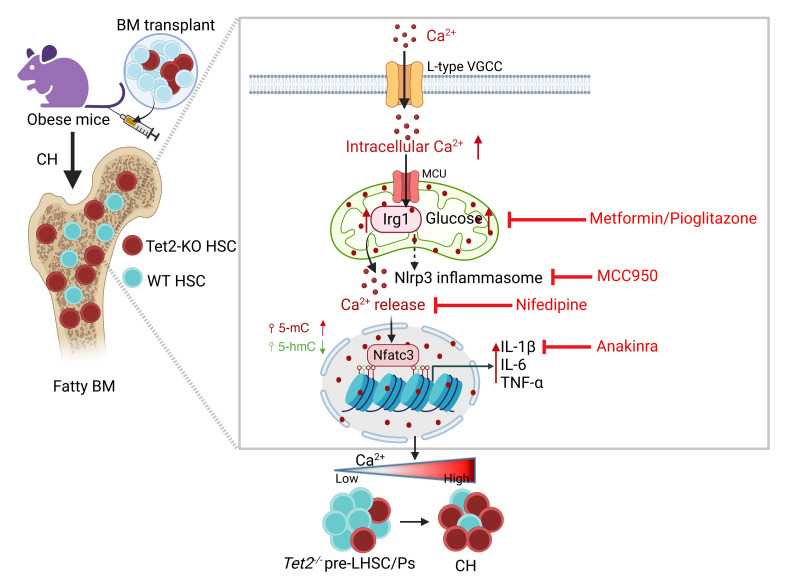

## Introduction

Clonal hematopoiesis of indeterminate potential (CHIP) is a newly-discovered condition that increases the risk of all-cause mortality and the development of hematologic malignancies (1, 2). The prevalence of CHIP increases with age, affecting more than 10%–20% of adults over 70 years of age (3). CHIP is characterized by the age-associated accumulation of somatic mutations in hematopoietic stem and progenitor cells (HSC/Ps). The most frequently mutated CHIP-associated genes in the hematopoietic system are ten-eleven translocation 2 (*TET2*), DNA methyltransferase 3A (*DNMT3A*), additional sex combs-like 1 (*ASXL1*), and Janus kinase 2 (*JAK2*). Mutations in these genes can provide a competitive advantage to cells carrying them, allowing for their clonal expansion in a process known as clonal hematopoiesis (CH) (2, 4). While normal HSCs have intact self-renewal and regeneration capacities with stable differentiation potential toward myeloid and lymphoid PCs, HSC/Ps carrying CHIP-associated mutations exhibit skewing toward the myeloid cell compartment (5). In addition to hematologic malignancies, epidemiological studies show that CH is also associated with an increase in mortality in patients with nonhematologic cancers (1, 6).

While CHIP-associated genes are mutated at high frequencies in patients with myeloproliferative neoplasms (MPNs) and acute myeloid leukemia (AML), these mutations are also detected in asymptomatic individuals (6). In fact, not all individuals carrying CHIP-associated mutations develop hematologic malignancies, suggesting that certain predisposing factors may promote clonal expansion and the development of CHIP-associated diseases. Recent studies indicate that common inflammatory disease states, such as obesity, diabetes, infection, smoking, and pulmonary inflammation, may contribute to the development of CHIP-associated pathologies, suggesting that environmental factors probably play a key role in CH emergence in HSC/Ps bearing CHIP-associated mutations; however, the mechanisms have not yet been fully elucidated (7–13). A better understanding of these processes will provide valuable information for the identification of individuals at risk for the development of CHIP-associated diseases and for the stratification of patients for treatment.

Obesity is one of the most prevalent diseases globally and is associated with type 2 diabetes mellitus (T2DM) and insulin resistance (IR). The main cause of T2DM is obesity-driven IR combined with inadequate secretion of insulin by pancreatic β cells (14, 15). Adipose tissue is an important reservoir of energy in the body, but its excessive accumulation contributes to the development of obesity in part by promoting a proinflammatory, hyperlipidemic, and insulin-resistant environment (14, 16). Although a clear picture of body fat distribution in individuals with obesity is critical for assessing obesity; waist-to-hip ratios (WHRs) and computed tomography (CT) scanning have become useful and commonly used tools to measure body fat distribution (16). Leptin (*Lep*) is a peptide hormone that is secreted exclusively by adipocytes and is essential for the regulation of body weight. A lack of either *Lep* or the leptin receptor (*Lepr*) in mice and humans results in obesity, hyperglycemia, and IR (17). Individuals with obesity or *Ob/Ob* mice have an increased accumulation of marrow adipocytes (fat cells), referred to as fatty bone marrow (FBM). FBM results in reduced bone mass by inhibiting osteoblast differentiation, leading to altered hematopoiesis (18, 19). Moreover, BM adipocytes secrete abnormal proinflammatory cytokines including IL-6, IL-1β, and TNF-α (20, 21).

Obesity has been linked to worse outcomes in various types of cancer, including hematologic malignancies (22, 23). However, the relationship between obesity and the growth and survival of pre-leukemic stem and progenitor cells (pre-LHSCs/PCs) remains unclear, and, more important, the relevant biological mechanisms of obesity-induced FBM transformation of pre-leukemic CHIP–bearing cells into more severe forms of MPN are not known. To investigate the relationship between CHIP and obesity, we analyzed several mouse models harboring common CHIP mutations: *Tet2*, *Dnmt3a*, *Asxl1*, or *Jak2* on the background of *Lep^Ob/Ob^* (*Ob/Ob*) mice to mimic the human pre-LHSCs/PCs condition occurring in individuals with obesity (24, 25). We show that transgenic compound mutant mice or *Ob/Ob* mice transplanted with BM containing CHIP-associated mutations (*Tet2^–/–^*, *Tet2^+/–^*, *Dnmt3a^+/–^*, *Asxl1^+/–^*, and *Jak2^V617F/+^*) developed rapid CH and a severe MPN-like phenotype, which was associated with increased expression of intracellular Ca^2+^/Irg1/Nfatc3 signaling in *Tet2*-mutant cells. We show that inhibition of members of the calcium signaling axis in pre-LHSCs/PCs using nifedipine (L-type voltage–gated calcium channel [VGCC] blocker) or SKF-96365 (store-operated calcium channel blocker), either alone or in combination with metformin (glucose inhibitor) or MCC950 (Nlrp3 inhibitor) or anakinra (IL-1 receptor antagonist), abrogated CHIP-associated pathologies, including the growth of mutant HSC/Ps and partially restored the growth of normal HSC/Ps.

## Results

### An increased presence of CHIP-associated mutations in individuals with obesity.

**Figure 1:**
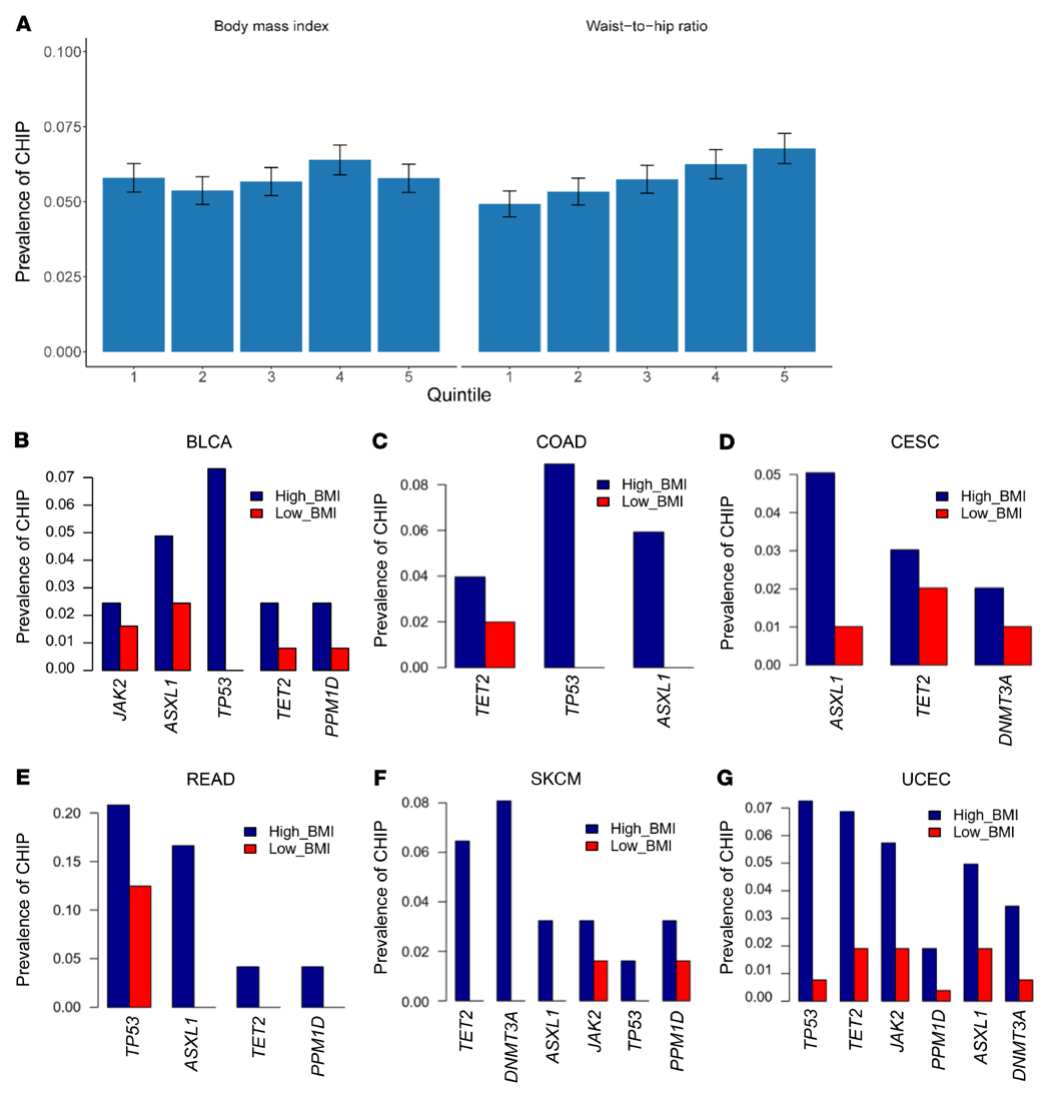
The prevalence of CHIP mutations is higher in patients with obesity. (**A**) Prevalence of CHIP mutations across quintiles of BMI and WHR in a cohort of 47,466 unrelated participants in the UK Biobank. The prevalence of CHIP increased with higher WHRs, and the percentage of participants with CHIP was 4.93%, 5.75%, and 6.56% in the lowest, middle, and highest WHR quintiles, respectively. (**B**–**G**) Higher frequencies of CHIP mutations in patients with a high BMI (>30 kg/m^2^) compared with patients with a low BMI (≤25 kg/m^2^). TCGA data were derived from patients with 1 of the following 6 types of cancer: BLCA, COAD, CESC, READ, SKCM, or UCEC.

The UK Biobank is a large population-based cohort with ongoing recruitment. Between 2006 and 2010, approximately 500,000 adult participants aged 40–69 years were recruited by one of 22 assessment centers across the United Kingdom. In the current analysis, we included 47,466 unrelated participants who were free of T2DM at baseline and had valid CHIP measurements in the UK Biobank. Details regarding this cohort have been described elsewhere (26). The mean (SD) age at enrollment was 56.5 years (8.0), 45.0% were male, 43.9% never smoked, and 82.6% self-reported as being of European descent. At baseline, the mean (SD) BMI was 27.3 kg/m^2^ (4.7), with 43.0% being overweight and 23.6% obese, and the overall mean (SD) WHR was 0.87 (0.09). CHIP was present in 5.8% of the study population, with the most common mutations detected in *DNMT3A* (3.7%) and *TET2* (1.0%) genes. A large CHIP clone was defined as a CHIP mutation with a variant allele fraction (VAF) of greater than 10% and was present in 2.4% of the study population. The prevalence of coronary artery disease (CAD), hypertension, and hypercholesterolemia was 2.4%, 29.1%, and 16.2%, respectively (Table 1). Individuals with CHIP mutations, on average, had a higher WHR. After adjusting for major covariates, the presence of the CHIP mutation was associated with a 0.0028 increase in WHR (*P* = 0.03). Figure 1A shows the CHIP burden among participants across quintiles of BMI and WHR. We observed that the prevalence of CHIP increased with higher WHRs: the percentage of participants with CHIP was 4.93%, 5.75%, and 6.56% in the lowest, middle, and highest WHR quintiles, respectively. Furthermore, the prevalence of the *TET2* mutation was significantly higher in individuals with a high BMI and WHR (Supplemental Table 1; supplemental material available online with this article; https://doi.org/10.1172/JCI163968DS1). We also examined the association between baseline obesity measured as BMI (*n* = 46,460) and WHR (*n* = 47,405) as well as the risk of developing future myeloid leukemia using the Cox proportional hazards model. The HR (95% CI) incident of myeloid leukemia per 1 SD increase in BMI was 1.71 (1.23, 2.38), with a *P* value of 1.31 × 10^–3^ and that of WHR was assessed to be 1.59 (0.91, 2.79), with a *P* value of 1.05 × 10^–1^ (Table 2). These results demonstrate that obesity is associated with myeloid leukemia.

We next interrogated the association between CHIP and nonhematologic cancers. In patients with nonhematologic cancers, CH is prevalent and associated with a poor prognosis (6). We assumed that if CHIP and BMI together form a risk factor, the prevalence of CHIP mutations would be higher in patients with a high BMI (>30 kg/m^2^) than in those with a low BMI (≤25 kg/m^2^). We examined The Cancer Genome Atlas (TCGA) database to determine whether CHIP was associated with patients with nonhematologic cancer with a higher BMI versus a lower BMI. We observed that all analyzed CHIP genes had a higher mutation rate in patients with a high BMI than in those with a low BMI among all 6 cancer types examined, including bladder cancer (BLCA), colon adenocarcinoma (COAD), cervical squamous cell carcinoma/endocervical adenocarcinoma (CESC), rectum adenocarcinoma (READ), skin cutaneous melanoma (SKCM), and uterine corpus endometrial carcinoma (UCEC). The following mutations were significantly higher in patients with high BMIs (*TP53* in BLCA: *P* = 0.003; *TP53* in COAD: *P* = 0.003; *DNMT3A* in SKCM: *P* = 0.05; and *TP53*: *P* = 0.0001; *TET2*: *P* = 0.009, *JAK2*: *P* = 0.03 in UCEC) compared with patients with low BMIs (Figure 1, B–G, and Supplemental Table 2). Furthermore, we tested the overall survival of patients with CHIP mutations versus that of patients without CHIP mutations for 7 different cancer types, namely breast cancer (BRCA), lung adenocarcinoma (LUAD), ovarian (OV) cancer, stomach adenocarcinoma (STAD), COAD, low-grade glioma (LGG), and liver hepatocellular carcinoma (LIHC). Patients were grouped according to whether they carried at least 1 CHIP mutation or no CHIP mutation. We observed that the presence of a CHIP mutation was significantly associated with worse overall survival (OS) for breast and lung cancer, whereas other cancer types showed a trend toward the presence of a CHIP mutation and an association with worse OS, but because of the small number of samples, significance was not reached (Supplemental Figure 1, A–G). These results suggest the clinical implication for patients carrying a CHIP mutation. Taken together, these data suggest that CHIP mutations occur with greater frequency in individuals with obesity and in obese patients with various solid tumors.

### Obesity induces the expansion and transformation of pre-LHSCs/PCs and exacerbates the development of a MPN-like phenotype.

**Figure 2:**
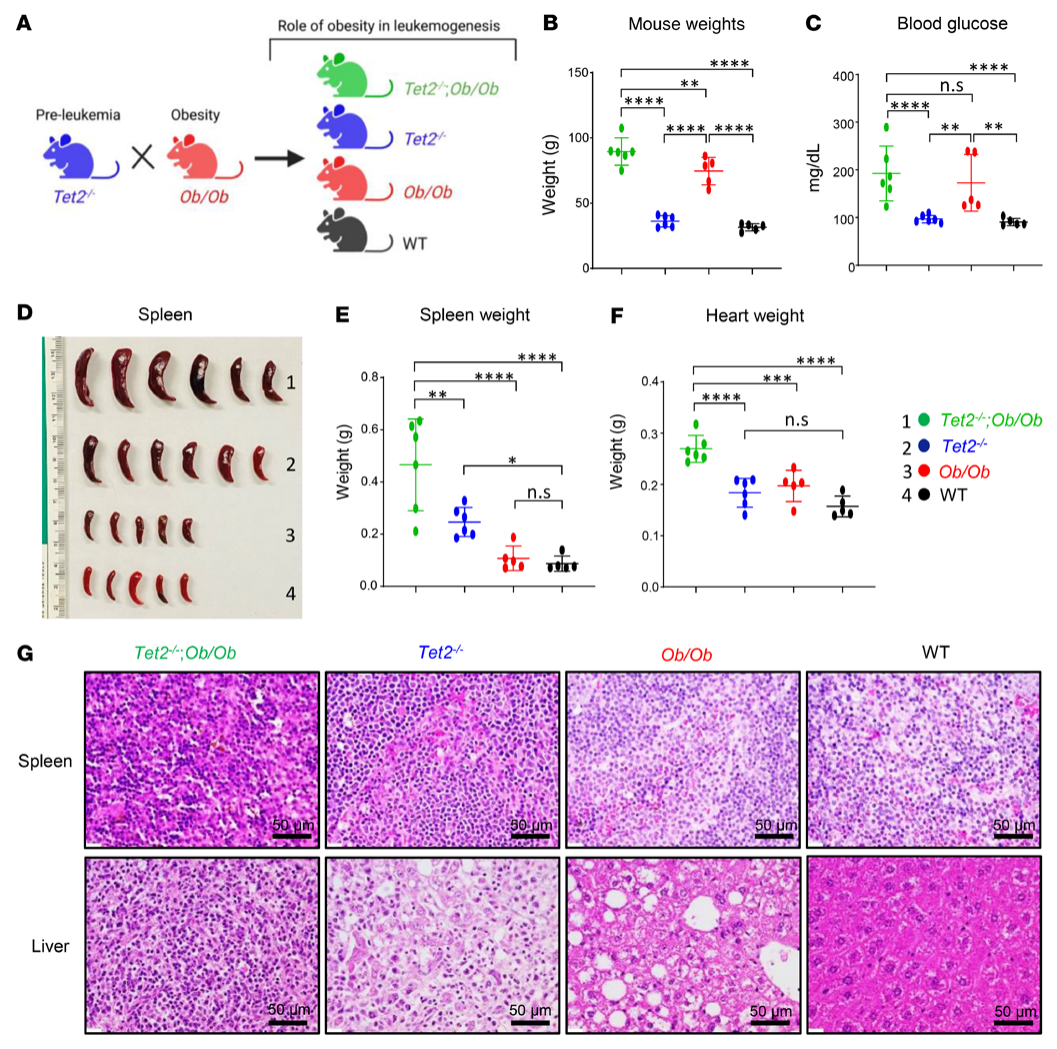
*Tet2* loss-of-function–driven CH exacerbates hyperglycemia and splenomegaly in obese mice. (**A**) Schematic demonstrating the generation of *Tet2^–/–^* *Ob/Ob* compound mutant mice along with *Tet2^–/–^*, *Ob/Ob*, and WT control mice. (**B**–**F**) Elevated body weights, fasting blood glucose levels, spleen weights, and heart weights were observed in *Tet2^–/–^* *Ob/Ob* compound mutant mice compared with mice in the other groups. (**G**) Histological analysis of H&E-stained sections of spleen and liver from a representative *Tet2^–/–^* *Ob/Ob* mouse along with controls (*n* = 5–6 mice per group). Scale bars: 50 μm. Data are shown as the mean ± SEM. *P < 0.05, **P < 0.005, ***P < 0.0005, and ****P < 0.0001, by 1-way ANOVA.

**Figure 3:**
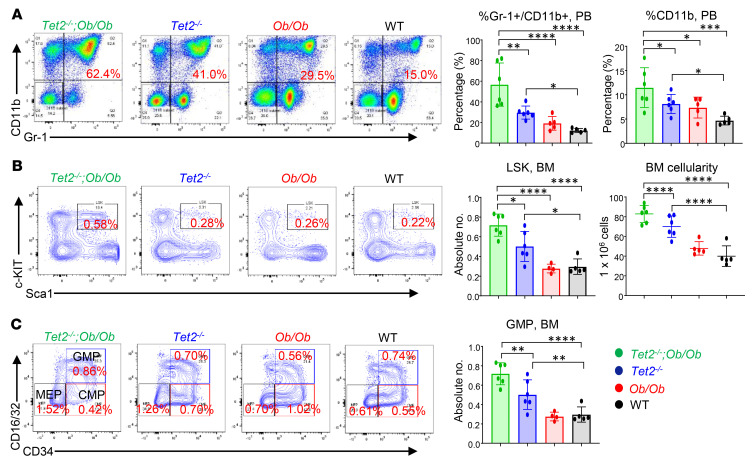
Obesity exacerbates the expansion of *Tet2^–/–^* pre-LHSCs/PCs. (**A**) Representative flow cytometry plots of PB mature myeloid cells (Gr-1^+^CD11b^+^) from mice of the indicated genotypes. An increased frequency of myeloid cells (Gr-1^+^CD11b^+^) and CD11b^+^ cells was observed in *Tet2^–/–^* *Ob/Ob* compound mutant mice compared with mice in the other groups. (**B**) Representative flow cytometry plots of BM LSK cells from mice of the indicated genotypes. Enhanced absolute numbers of total LSK cells and BM cellularity were observed in *Tet2^–/–^* *Ob/Ob* mice compared with mice in the other groups. (**C**) Representative flow cytometry plots of BM progenitors from mice of the indicated genotypes. Enhanced absolute numbers of total GMPs (Lin^−^c-KIT^+^CD16/CD32^+^CD34^+^) were observed in *Tet2^–/–^* *Ob/Ob* mice compared with mice in the other groups (*n* = 5–6 mice per group). Data are shown as the mean ± SEM. *P < 0.05, **P < 0.005, ***P < 0.0005, and ****P < 0.0001, by 1-way ANOVA.

To determine the biological consequence(s) of obesity on CHIP-bearing HSC/Ps, we generated obese FBM (leptin-deficient *Ob/Ob*) mice possessing *Tet2^–/–^* or *Dnmt3a^+/–^* mutations to mimic the human pre-leukemic condition in the context of obesity. *Ob^+/–^* mice (both males and females) were crossed with *Tet2^–/–^ mice* (males and females), and the F1 progeny of these matings (*Tet2^+/−^* *Ob^+/–^* mice) were then backcrossed to obtain *Tet2^–/–^ Ob/Ob* compound mutant mice and controls (Figure 2A). We analyzed compound mutant mice showing signs of disease. As expected, we observed increased body weight and fasting blood glucose levels in the obese mice (Figure 2, B and C). These mice also exhibited increases in peripheral blood (PB) WBCs, neutrophils, and monocytes and a reduction in lymphocytes compared with control mice (Supplemental Figure 2, A–E). Figure 2, D–F, shows severe splenomegaly and increased heart weights in *Tet2^–/–^* *Ob/Ob* compound mutant mice compared with controls. Additionally, histopathologic examination of H&E-stained spleens and liver sections from *Tet2^–/–^* *Ob/Ob* mice showed extensive infiltration of mutant myeloid cells, which resulted in disruption of the splenic and hepatic architecture in these mice compared with controls (Figure 2G). It is important to note that, in spite of *Ob/Ob* mice being twice as heavy as WT mice, their spleen sizes were similar to those of WT mice (Figure 2, D and E) (27). Moreover, the compound mutant mice also showed an increase in the frequency of Gr-1^+^CD11b^+^ double-positive cells as well as more mature myeloid cells (CD11b^+^) in the PB (Figure 3A), BM (Supplemental Figure 2F), and spleen (Supplemental Figure 2G). In contrast, B220^+^ B cells were significantly reduced in the BM and spleens of these mice compared with controls (Supplemental Figure 2H). Importantly, the compound mutant mice also showed an increased presence of myeloid blasts as reflected by the presence of c-KIT^+^CD11b^+^ double-positive cells in the BM and spleen (Supplemental Figure 2I) as well as in the absolute number of total lineage-negative (Lin^–^), Sca-1^+^, c-KIT^+^ (LSK) cells in the BM and overall BM cellularity (Figure 3B). Moreover, the absolute number of total HPC-1 (LSK CD48^+^CD150^−^ cells) long-term hematopoietic stem cells (LT-HSCs) (LSK CD48^−^CD150^+^ cells) (Supplemental Figure 2J) and granulocyte-macrophage progenitors (GMPs) (Lin^–^c-KIT^+^ [LK] CD16^+^/CD32^+^CD34^+^ cells) were significantly increased in the compound mutant mice compared with the absolute numbers in control mice (Figure 3C).

We extended these studies to determine whether other CHIP mutations such as *Dnmt3a* also result in similar outcomes in the setting of obesity. To obtain the *Dnmt3a^+/–^* Mx1-Cre^+^ *Ob/Ob* compound mutant mice and control mice, we crossed *Ob^+/–^* mice (both males and females) with *Dnmt3a^+/–^* Mx1-Cre^+^ mice (males and females), and the F1 progeny of these matings were then backcrossed (Supplemental Figure 3A). We administered polyinosine-polycytidine (pIpC) to conditionally delete 1 of the *Dnmt3a* alleles. At 3 months, we observed elevated WBC, neutrophil, and monocyte counts in the *Dnmt3a^+/–^* Mx1-Cre^+^ *Ob/Ob* compound mutant mice compared with the controls (Supplemental Figure 3, B–D). These mice were sacrificed 22 weeks after pIpC treatment. We observed splenomegaly and increased heart weights, body weights, and blood glucose levels in the compound mutant mice compared with control mice (Supplemental Figure 3, E–H). We also observed an increased frequency of Gr-1^+^CD11b^+^ double-positive mature myeloid cells in the PB and BM of these compound mutant mice compared with controls (Supplemental Figure 3, I and J). Notably, these compound mutant mice also had an increased presence of myeloid blasts (c-KIT^+^CD11b^+^ cells) in the BM (Supplemental Figure 3K). The absolute numbers of total LSK cells and GMPs were also significantly increased in the compound mutant mice compared with absolute numbers in the controls (Supplemental Figure 3, L and M). These data suggest that FBM in obese mice induced changes in the compound mutant mice to stimulate the growth of *Dnmt3a*-bearing pre-LHSCs/PCs, similar to what we observed in *Tet2*-deficient HSC/Ps. Together, these data suggest that obesity induced the expansion and transformation of pre-LHSCs/PCs bearing *Dnmt3a* or *Tet2* mutations and contributed to the development of a severe MPN-like phenotype.

### Obesity-induced FBM promotes the expansion of CHIP-mutant clones in mice.

**Figure 4:**
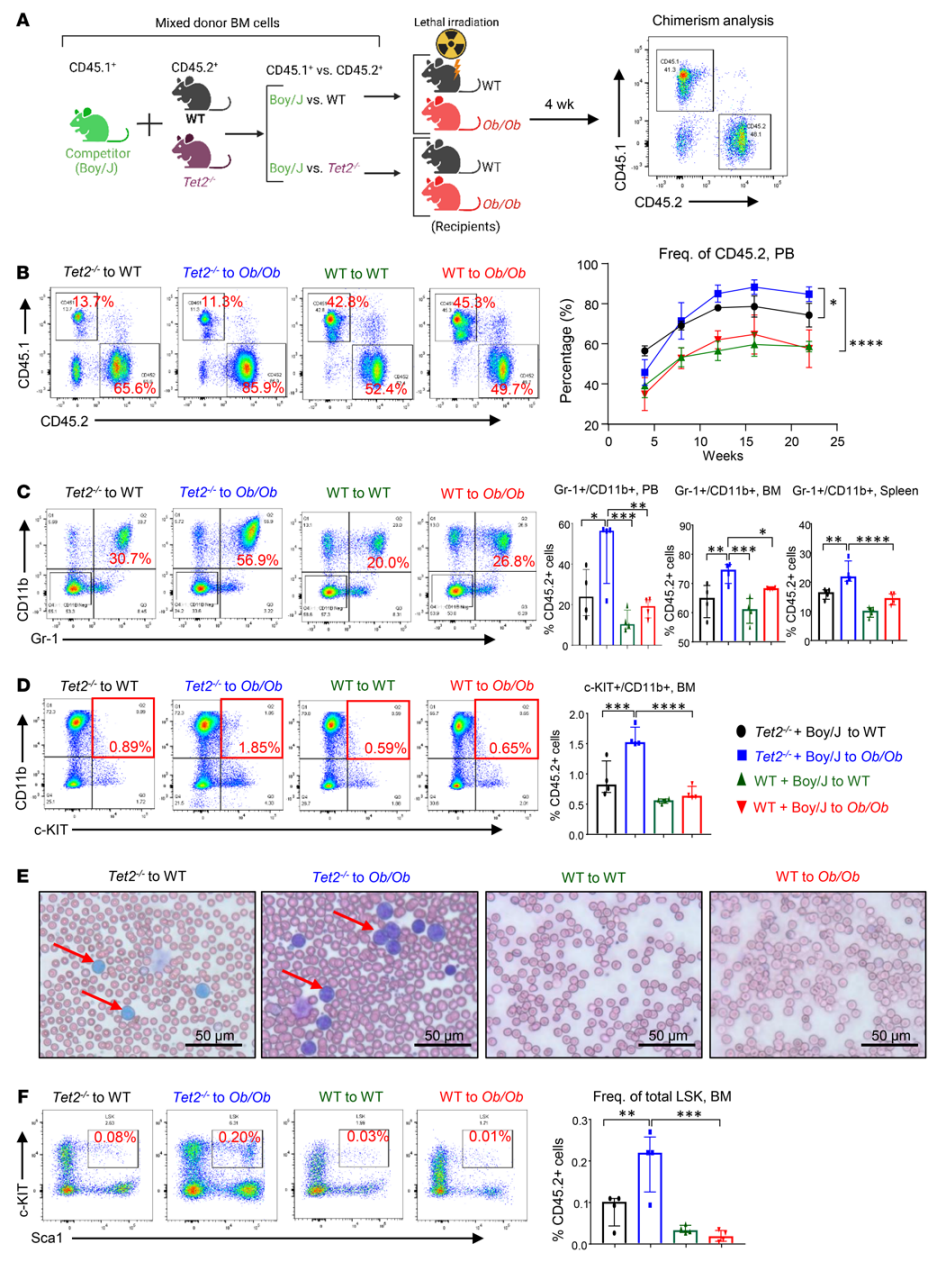
*Tet2* deficiency confers a competitive advantage to HSC/Ps in obese recipient mice. (**A**) Schematic of the competitive BMT assay. Donor cells from *Tet2^–/–^* or WT mice were mixed with Boy/J cells, and a competitive BMT assay was performed using lethally irradiated WT or *Ob/Ob* mice as recipients. Donor-derived chimerism was observed using antibodies against CD45.1^+^ or CD45.2^+^. (**B**) Representative flow cytometry profiles for donor chimerism in the PB of recipient mice measured every 4 weeks, and quantification of CD45.2^+^ cells in the PB of the indicated recipient mice. (**C**) Representative flow cytometry profile of Gr-1^+^CD11b^+^ double-positive cells in the PB of the indicated recipient mice, and frequency of myeloid cells in the PB and BM and spleens of competitive transplant recipients over 22 weeks. (**D**) Representative flow cytometry plots of myeloid blasts (c-KIT^+^CD11b^+^ double-positive cells) and frequency of myeloid blast cells in the BM of competitive transplant recipients over 22 weeks. (**E**) PB smears from the indicated recipient mice 22 weeks after BMT. Scale bars: 50 μm. (**F**) Representative flow cytometry profile of LSK cells in the BM from the indicated recipient mice, and frequency of LSK cells in the BM of competitive transplant recipients over 22 weeks (*n* = 4 mice per group). Data are shown as the mean ± SEM. *P < 0.05, **P < 0.005, ***P < 0.0005, and ****P < 0.0001, by 1-way ANOVA. Freq., frequency.

Although the above studies demonstrated a role of obesity in driving the growth of *Tet2*- and *Dnmt3a*-mutant HSC/Ps and a MPN-like phenotype in transgenic mice, we next sought to explore whether obesity contributes to CH and whether the expansion of mutant HSC/Ps is driven by FBM in a microenvironment-dependent manner. We used a competitive BM transplantation (BMT) assay that enables the measurement of clonal expansion , as described previously by us and others (13, 28–30). Under these transplantation settings, *Tet2*-mutant HSC/Ps outcompete WT HSC/Ps when transplanted at a 1:1 ratio (13, 28–30). We transplanted a mixture of WT or *Tet2^–/–^* and Boy/J BM cells (1:1 ratio) into lethally irradiated FBM mice or WT lean recipients (Figure 4A). Donor chimerism in the PB of the recipient mice was determined at the indicated time points by flow cytometry. We found that the frequency of donor-derived (CD45.2^+^) *Tet2^–/–^* cells was significantly increased in *Ob/Ob* recipients compared with that detected in control mice (Figure 4B). The increase in CD45.2^+^ *Tet2^–/–^* donor cells was also observed in the BM and spleens of *Ob/Ob* mice compared with controls (Supplemental Figure 4A). Upon sacrifice, *Ob/Ob* recipient mice bearing *Tet2^–/–^* cells showed increased spleen weights, WBC counts, and neutrophil and monocyte counts and reduced lymphocytes compared with control groups (Supplemental Figure 4, B–F). Mature myeloid cells (Gr-1^+^CD11b^+^ double-positive population) were significantly increased in the PB, BM, and spleens of these recipients (Figure 4C). Importantly, these mice also had myeloid blasts, as reflected by the presence of c-KIT^+^CD11b^+^ double-positive cells in the BM and in PB smears, consistent with the development of a severe MPN-like phenotype (Figure 4, D and E). Moreover, the frequency of total LSK (Figure 4F) and HPC-1 (LSK CD48^+^CD150^−^) cells (Supplemental Figure 4G) and the absolute number of common myeloid progenitors (CMPs) (LK CD16/CD32^−^CD34^+^) in the BM were significantly increased in *Ob/Ob* recipients reconstituted with *Tet2^–/–^* cells compared with control groups (Supplemental Figure 4H). Furthermore, serum cytokine analysis of these mice revealed a significant increase in the presence of proinflammatory cytokines such as IL-6, IL-1β, TNF-α, IL-5, and G-CSF compared with control groups (Supplemental Figure 4I). These findings suggest that FBM in obese mice provided selective clonal advantage to *Tet2^–/–^* pre-LHSCs/PCs through increased self-renewal and accelerated the development of a MPN-like phenotype compared with lean WT recipient mice.

**Figure 5:**
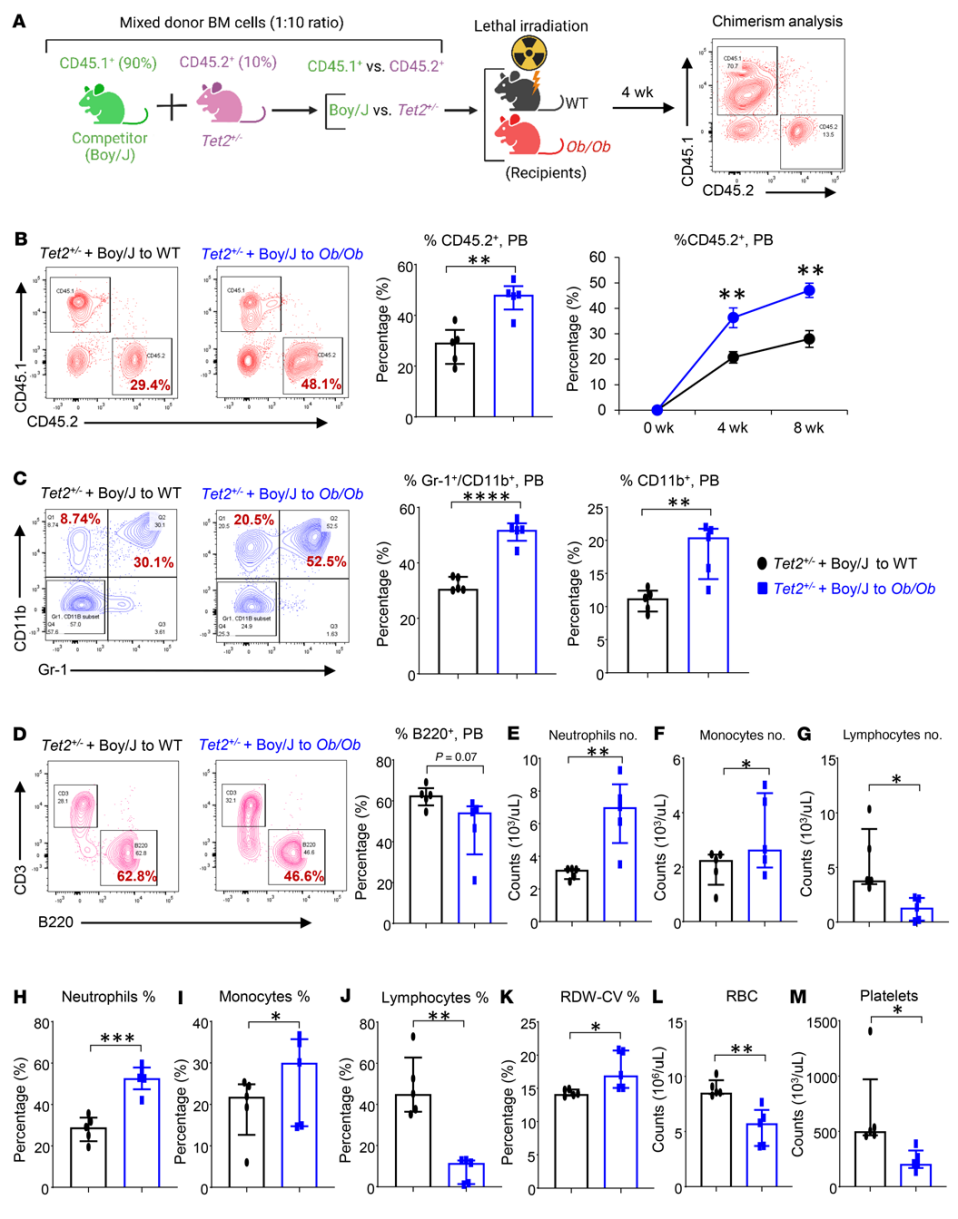
*Tet2^+/–^* mutant pre-LHSCs/PCs outcompete WT cells and promote CH in obese recipient mice. (**A**) Schematic of the competitive BMT assay. Donor BM cells (10%) from *Tet2^+/–^* mice were mixed with Boy/J BM cells (90%), and a competitive BMT assay was performed using lethally irradiated WT and *Ob/Ob* mice as recipients. Donor-derived chimerism was observed using antibodies against CD45.1^+^ or CD45.2^+^. (**B**) Representative flow cytometry profiles for donor chimerism (CD45.1^+^CD45.2^+^ cells) in the PB of recipient mice measured every 4 weeks, and quantification of CD45.2^+^ cells in the PB of the indicated recipient mice. (**C**) Representative flow cytometry profile of myeloid cells (Gr-1^+^CD11b^+^ cells) in the PB of the indicated recipient mice, and frequency of Gr-1^+^CD11b^+^ double-positive myeloid cells in PB and of CD11b^+^ myeloid cells in PB of competitive transplant recipients over 8 weeks. (**D**) Representative flow cytometry plots of lymphocytes (CD3^+^B220^+^ cells) from the indicated recipient mice, and frequency of B220^+^ B lymphocytes in the PB of competitive transplant recipients over 8 weeks. (**E**–**M**) PB counts of (**E**) neutrophils, (**F**) monocytes, and (**G**) lymphocytes; percentages of (**H**) neutrophils, (**I**) monocytes, (**J**) lymphocytes, and (**K**) RDWs; and (**L**) RBC and (**M**) platelet counts in the indicated recipients 8 weeks after competitive BMT (*n* = 5 mice per group). Data are shown as the mean ± SEM. *P < 0.05, **P < 0.005, ***P < 0.0005, and ****P < 0.0001, by 2-tailed Student’s *t* test.

Given that most of the *TET2* mutations in individuals with CHIP or in patients with chronic myelomonocytic leukemia (CMML), AML, or MPN are heterozygous in nature (*TET2^+/–^*), we next tested whether transplantation of a lower percentage of *Tet2^+/–^* pre-LHSCs/PCs (1:10 ratio) also results in their expansion at a higher rate in *Ob/Ob* FBM mice, similar to what we observed in *Tet2^–/–^* pre-LHSCs/PCs. To test this, we transplanted a mixture of unfractionated BM cells containing 10% CD45.2^+^ *Tet2^+/–^* cells and 90% CD45.1^+^ cells from Boy/J competitor mice into lethally irradiated *Ob/Ob* or WT recipients, a true measure of clonal expansion (Figure 5A) (11). Donor-derived *Tet2^+/–^* CD45.2^+^ cells rapidly increased in the PB of *Ob/Ob* mice compared with that of their WT counterparts (CD45.1^+^ cells) and compared with cells transplanted into WT recipient mice (Figure 5B). Further, *Ob/Ob* mice transplanted with *Tet2^+/–^* BM cells showed higher frequencies of Gr-1^+^CD11b^+^ double-positive myeloid cells and monocytic skewing (increase in Gr1^–^CD11b^+^ cells) (Figure 5C) and a decrease in B220^+^ B lymphocytes in the PB compared with WT recipient mice (Figure 5D). Similarly, PB counts revealed that *Ob/Ob* mice transplanted with *Tet2*^+/–^ cells had increased absolute numbers and frequencies of neutrophils, monocytes, and red cell distribution width-coefficient of variation (RDW-CV) and reduced numbers of lymphocytes, RBCs, and platelets compared with WT recipient mice (Figure 5, E–M). An increase in RDW is a hallmark of individuals bearing *TET2* mutations (31). Taken together, both *Tet2^–/–^* and *Tet2^+/–^* HSC/Ps outcompeted WT cells in *Ob/Ob* recipients compared with WT lean recipients, suggesting that the FBM observed in obese mice played an important role in CH. To test this further and to determine whether donor mice bearing heterozygous CHIP mutations, including *Dnmt3a*, *Asxl1*, and *Jak2*, also contribute to superior clonal expansion in obese mice, we carried out experiments identical to those described above for *Tet2*-mutant donors. In every scenario, BM cells bearing CHIP mutations outcompeted WT BM cells and resulted in increased myeloid cell skewing as well as expansion of pre-LHSCs/PCs (Supplemental Figure 5, A–L, Supplemental Figure 6, B–D, Supplemental Figure 7, A–D, Supplemental Figure 8, A and B, and Supplemental Figure 9, A–G). Importantly, we observed a significant increase in the frequency of c-KIT^+^/CD11b^+^–expressing myeloid blasts in each of the above-described mutation-bearing cells (Supplemental Figure 6A, Supplemental Figure 7E, and Supplemental Figure 9D). All of these phenotypes were associated with a significant increase in proinflammatory cytokines that were either unique to a specific CHIP mutation or were commonly observed in mice bearing different CHIP mutation–bearing HSC/Ps (Supplemental Figure 6E and Supplemental Figure 8C). These observations with regard to the upregulation of proinflammatory cytokines as well as RDW values were remarkably similar to the ones reported in humans bearing different CHIPs (31). Our data on *Dnmt3a* and *Asxl1* heterozygous HSC/Ps in *Ob/Ob* recipient mice were also remarkable because normal *Dnmt3a^+/–^* or *Asxl1^+/–^* mice do not manifest any phenotype and show no signs of overt CH or hematologic malignancy (32, 33).

### Obesity modulates Ca^2+^ levels in Tet2^–/–^ HSC/Ps.

**Figure 6:**
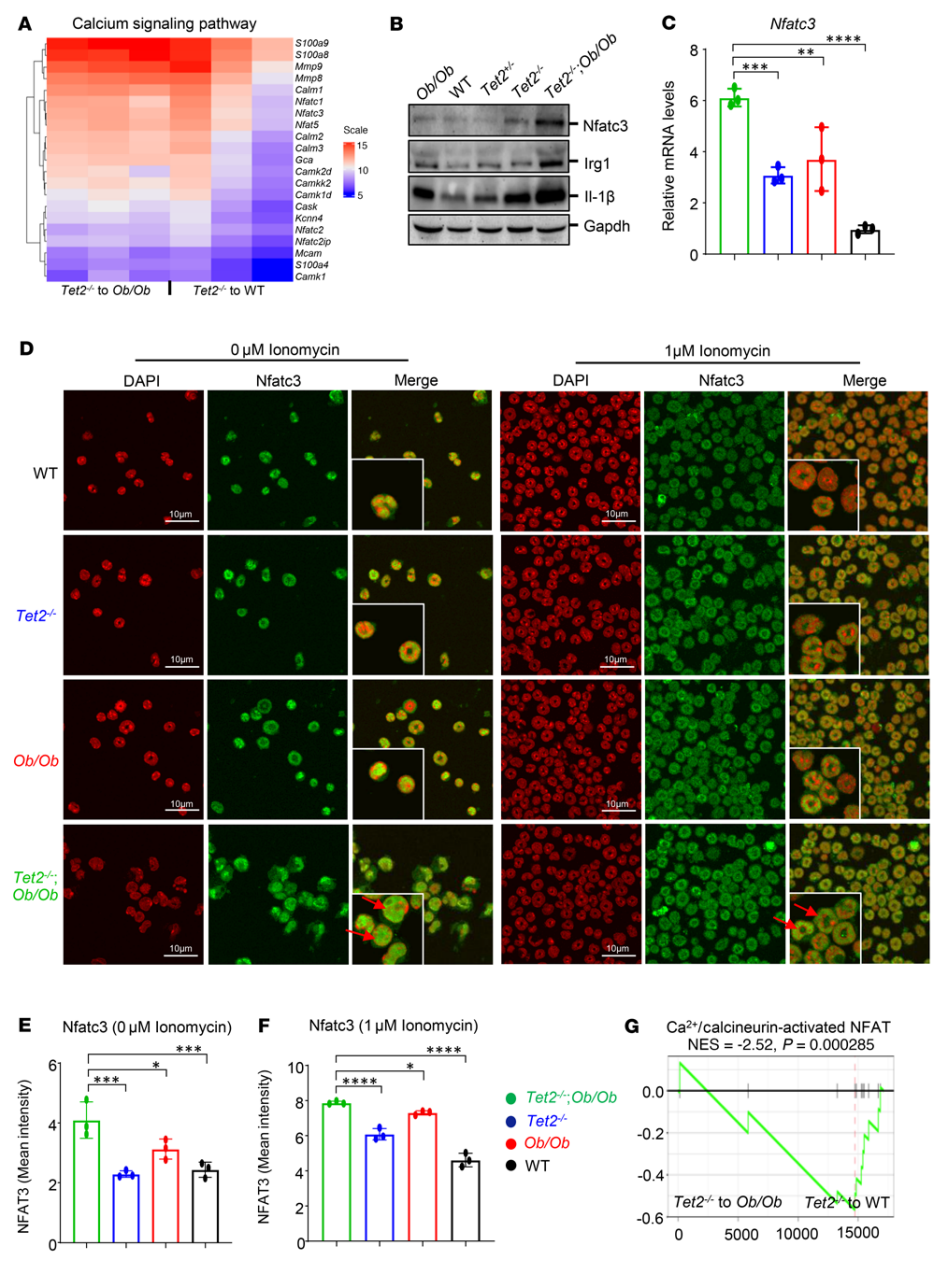
The Ca^2+^ signaling pathway is enriched in *Ob/Ob* mice bearing *Tet2^–/–^* cells. (**A**) Heatmap of the expression of calcium channel–related genes in obese and WT mice bearing *Tet2^–/–^* cells. (**B**) WB analysis was performed to measure the protein levels of Nfatc3, Irg1, and IL-1β in BM cells from *Tet2^–/–^* *Ob/Ob,* *Tet2^–/–^*, *Ob/Ob*, and WT mice. (**C**) Expression of the *Nfatc3* gene in *Tet2^–/–^* *Ob/Ob* compound mutant mice and control mice BM cells was detected by qRT-PCR. (**D**) HSC/Ps from *Tet2^–/–^* *Ob/Ob,* *Tet2^–/–^*, *Ob/Ob*, and WT mice were stimulated or not with 1 μm ionomycin, and Nfatc3 (green) localization was visualized by confocal microscopy. DAPI (red, pseudocolor) was used to stain the nucleus. Red arrow indicate nuclear translocation of *Nfat3*. Scale bars: 10 μM . The insets show the zoomed images (×2). (**E** and **F**) Quantification of the percentage of cells with nuclear Nfatc3 per high-power field using Fiji (ImageJ, NIH). (**G**) GSEA plot of gene sets related to the Ca^2+^/calcineurin-activated NFAT pathway that were upregulated in *Ob/Ob* mice bearing *Tet2^–/–^* cells compared with WT mice. NES, normalized enrichment score Data are shown as the mean ± SEM. *P < 0.05, **P < 0.005, ***P < 0.0005, and ****P < 0.0001, by 1-way ANOVA.

**Figure 7:**
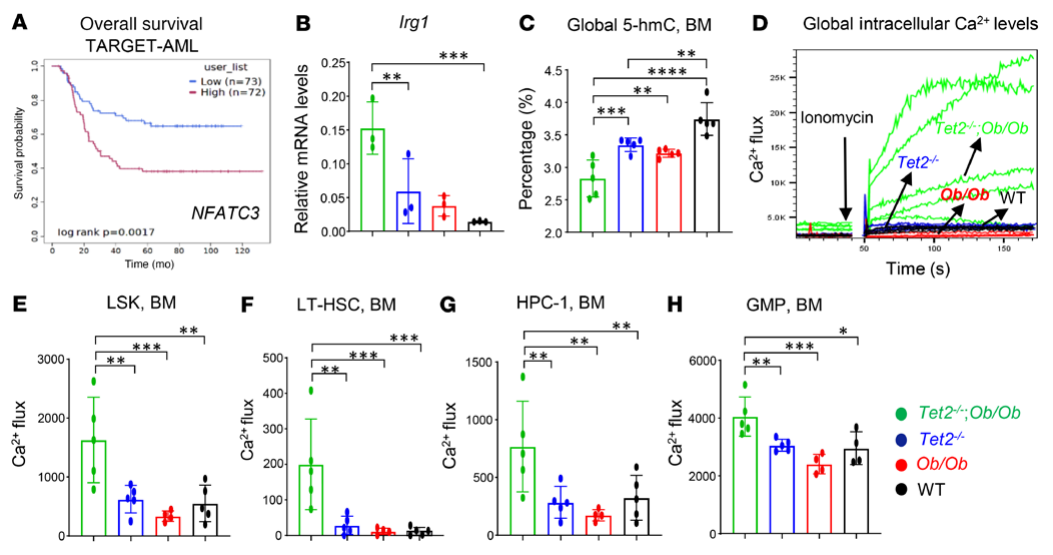
Obesity modulates Ca^2+^ levels in *Tet2^–/–^* HSC/Ps. (**A**) Data from TARGET-AML (Survival Genie, https://bbisr.shinyapps.winship.emory.edu/SurvivalGenie/) were used to analyze the relationship between disease-free survival and *NFATC3* expression in patients with AML. (**B**) Expression of the *Irg1* gene in *Tet2^–/–^* *Ob/Ob* compound mutant mice and control mice BM cells was detected by qRT-PCR. (**C**) Quantification of 5-hmC levels in HSC/Ps derived from *Tet2^–/–^* *Ob/Ob* compound mutant mice compared with other groups. (**D**–**H**) Quantification of intracellular Ca^2+^ levels in the global BM and different HSC/Ps compartments. BM cells were stained with Calbryte AM-520 and induced with 1 μM ionomycin and gated on LSK, HPC-1, LT-HSC, and GMP cell populations. Representative flow kinetics plots demonstrate the changes in Ca^2+^ flux in global BM cells (**D**) and quantification of Ca^2+^ levels in LSK cells (**E**), LT-HSCs (**F**), HPC-1 cells (**G**), and GMPs (**H**) in BM from mice of the indicated genotypes (*n* = 3–6 mice per group). Data are shown as the mean ± SEM. *P < 0.05, **P < 0.005, ***P < 0.0005, and ****P < 0.0001, by 1-way ANOVA.

We next focused our efforts on understanding the mechanism or mechanisms by which obesity drives CH in HSC/Ps bearing CHIP mutations and used the *Tet2*-mutant model to study this. We isolated total RNA from whole BM cells from WT mice and from *Ob/Ob* FBM mice that had been transplanted with *Tet2^–/–^* pre-LHSCs/PCs and performed bulk RNA-Seq. As shown in Supplemental Figure 1H, RNA-Seq analyses revealed that a set of differentially expressed genes (DEGs) were upregulated in *Ob/Ob* mice bearing *Tet2^–/–^* cells compared with WT cells. A comprehensive list of the RNA-Seq analyses, including the DEGs and pathway enrichment analysis, is included in Supplemental Tables 4 and 5. We analyzed the DEGs in *Ob/Ob* and WT mice bearing *Tet2^–/–^* cells and subjected them to ClueGo analysis (34) on the Cytoscape platform (35). A total of 433 DEGs representing known pathways for calcium signaling, PI3K/AKT signaling, RAS signaling, MAPK signaling, PPAR signaling, insulin secretion, and dilated and hypertrophic cardiomyopathy signaling were upregulated in *Ob/Ob* mice bearing *Tet2^–/–^* cells compared with WT recipient mice (Supplemental Figure 1I). Furthermore, protein-protein interaction (PPI) network maps were constructed from both downregulated and upregulated genes to investigate the nature of each gene in the network for their connectivity and to identify the hot spots/hub nodes of the PPI networks. The PPI maps were further subjected to clustering analysis using the Molecular Complex Detection (MCODE) application (36), followed by CytoHubba analysis (37) in the Cytoscape environment. The top 50 upregulated genes were extracted, and their significance to the relevant pathways described above was interpreted (Supplemental Figure 1J). This analysis revealed genes involved in calcium signaling, EGFR (*Egfr*) signaling, Erb-B2 receptor tyrosine kinase 2 (*Erbb2*) signaling, and calcium/calmodulin-dependent protein kinase II β (*Camk2b*) signaling, the latter *Camk2b* being an intracellular kinase triggered by inflammatory stimuli that respond to intracellular Ca^2+^ changes. Increased intracellular Ca^2+^ can bind to calmodulin and activate the phosphatase calcineurin and *Camk2* (38), which dephosphorylates calcium-dependent nuclear factor of activated T cells (NFAT) and induces its translocation into the nucleus (39). *NFAT* is an important Ca^2+^ channel–related family gene associated with tumor development and progression (40). Ingenuity Pathway Analysis (IPA) showed that the Ca^2+^ signaling pathway was enriched and one of the top 5 signaling pathways in *Ob/Ob* mice bearing *Tet2^–/–^* cells (Supplemental Figure 1K). A heatmap of DEGs revealed that Ca^2+^ signaling–related gene expression was higher in *Ob/Ob* mice bearing *Tet2^–/–^* cells than in WT recipient mice (Figure 6A). Western blot (WB) and quantitative reverse transcription PCR (qRT-PCR) analysis revealed that *Tet2^–/–^* *Ob/Ob* HSC/Ps had increased expression of nuclear factor of activated T cells 3 (*Nfatc3*) compared with expression in control cells (Figure 6, B and C). Confocal microscopy analysis showed that nuclear translocation of *Nfatc3* was significantly higher in *Tet2^–/–^* *Ob/Ob* HSC/Ps than in control HSC/Ps (Figure 6, D–F). Furthermore, gene set enrichment analysis (GSEA) showed that the Ca^2+^/calcineurin-activated NFAT pathway was upregulated in *Ob/Ob* mice bearing *Tet2^–/–^* HSC/Ps compared with WT recipients (Figure 6G). Last, TARGET-AML data from the Survival Genie website (https://bbisr.shinyapps.winship.emory.edu/SurvivalGenie/) revealed poor survival rates for patients with AML who had high *NFATC3* expression levels (Figure 7A).

Prior studies have shown that loss of Tet2 results in the upregulation of IL-1β, a proinflammatory cytokine known to interfere with insulin signaling (41). Obesity is also associated with T2DM and IR (14). In agreement with these observations, our WB analysis revealed higher IL-1β protein levels in obese mice carrying Tet2-deficient cells (*Tet2^–/–^* *Ob/Ob*) compared with controls (Figure 6B). Furthermore, abnormal glucose availability can cause higher levels of acetyl-CoA, which frequently increases the influx of calcium into cells, leading to the activation and translocation of NFAT into the nucleus and resulting in the recruitment of the lysine acetyltransferase (KAT) p300, which drives site-specific regulation of H3K27ac followed by increased expression of genes associated with cell adhesion and migration including induction of immune-response gene 1 (*Irg1*), which leads to the accumulation of itaconate (42). Itaconate is an antiinflammatory metabolite that binds to the same site as α-ketoglutarate (α-KG) in TET enzyme and inhibits its catalytic activity during the process of active DNA demethylation (40, 42–45). These observations suggest a potential link between intracellular metabolism and epigenetic modifications. Consistent with these observations, we found that *Tet2^–/–^* *Ob/Ob* compound mutant HSC/Ps had higher expression of *Irg1* compared with HSC/Ps from control mice (Figure 6C and Figure 7B).

A recent study showed a significant reduction in 5-hydroxymethylcytosine (5-hmC) levels in pregnant obese (BMI >30 kg/m^2^) women compared with nonobese (BMI <25 kg/m^2^) controls (46). In mice, the expression of epigenetic genes in the placenta is affected by obesity, suggesting that obesity is associated with an alteration of epigenetic markers (47). Following the discovery that TET proteins convert 5-methylcytosine (5-mC) to 5-hmC, it has been shown that TET-mediated active DNA demethylation requires α-KG and ascorbate as cofactors (48, 49). Along these lines, we observed a reduction in global 5-hmC levels in HSC/Ps derived from *Tet2^–/–^ Ob/Ob* mice compared with HSC/Ps from *Tet2^–/–^*, *Ob/Ob*, or WT mice (Figure 7C). Given that reduced 5-hmC is a hallmark of several myeloid malignancies that often exhibit reduced levels of TET2, including in AML, MPN, and CMML (50, 51), and given the link between intracellular metabolism, epigenetic modifications, and increased Ca^2+^ influx in cells, we speculated that a reduction in 5-hmC in *Tet2^–/–^ Ob/Ob* HSC/Ps is at least associated with increased levels of Ca^2+^ in HSC/Ps in these mice. To test this hypothesis, we examined intracellular Ca^2+^ levels in different HSC/PC populations from the BM of *Tet2^–/–^* *Ob/Ob* mice using Calbryte 520 AM dye (FITC conjugated) and flow cytometry. Notably, intracellular Ca^2+^ levels were significantly upregulated in total BM cells, LSK cells, LT-HSCs, HPC-1 cells, and GMPs of *Tet2^–/–^ Ob/Ob* compound mutant mice compared with controls (Figure 7, D–H). These data suggest that, among the enrichment of many pathways (Supplemental Figure 1K), loss of Tet2 in pre-LHSCs/PCs under conditions of obesity induced higher intracellular levels of Ca^2+^, which was associated with reduced 5-hmC levels and exacerbated CH compared with control HSC/Ps.

### Nifedipine inhibits exacerbation of CH in combination with metformin, MCC950, and anakinra.

We show that both the compound mutant mice (*Tet2^–/–^* *Ob/Ob* and *Dnmt3a^+/–^* *Ob/Ob*) and the *Ob/Ob* mice that were transplanted with CHIP-mutant BM (*Tet2^+/–^*, *Tet2^–/–^*, *Dnmt3a^+/–^*, *Asxl1^+/–^*, and *Jak2^+/–^*) had exacerbated CH and a MPN-like phenotype. This was associated with an upregulation of proinflammatory cytokines such as *Il1β*, *Il6*, and *Tnfα* as well as of Ca^2+^ levels, enrichment of PPAR signaling, and elevated glucose levels. To determine whether CH would be affected by inhibiting these pathways pharmacologically, either with an individual drug or a combination of drugs, including pathways for the suppression of glucose and calcium levels as well as of expression of proinflammatory cytokines, we evaluated the therapeutic benefits of blocking these pathways using both individual and combination drug treatments.

**Figure 8:**
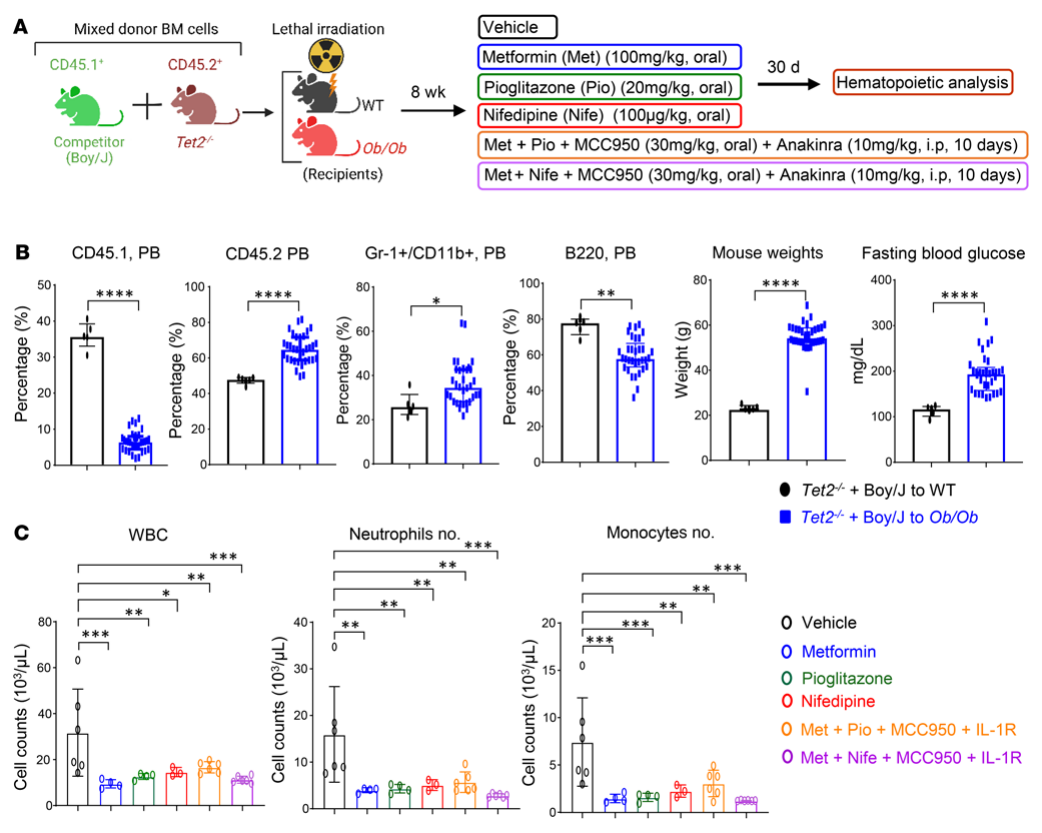
Combination of metformin, nifedipine, MCC950, and anakinra treatment reduces *Tet2^–/–^* myeloid cells in *Ob/Ob* mice. (**A**) Schematic of the competitive BMT assay and drug treatment. (**B**) Percentages of CD45.1, CD45.2, and myeloid Gr-1^+^/CD11b^+^ cells and B220^+^ B cells in the PB of the indicated recipients over 8 weeks of BMT, and body weights and fasting blood glucose levels in the indicated recipients over 8 weeks of BMT. (**C**) PB WBC, neutrophil, and monocyte counts in the indicated recipients after 30 days of the indicated drug treatments (*n* = 3–6 mice per group). Data are shown as the mean ± SEM. *P < 0.05, **P < 0.005, ***P < 0.0005, and ****P < 0.0001, by 2-tailed Student’s *t* test (**B**) or 1-way ANOVA (**C**).

**Figure 9:**
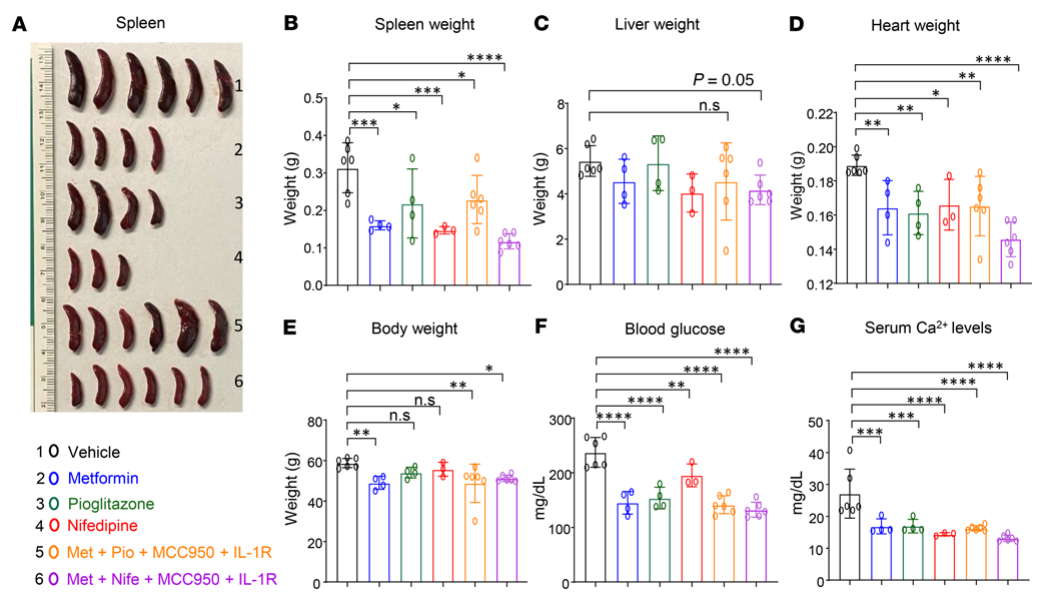
Combination of metformin, nifedipine, MCC950, and anakinra treatment reduces splenomegaly and hyperglycemia. (**A**) Spleen images, (**B**) spleen weights, (**C**) liver weights, (**D**) heart weights, (**E**) body weights, (**F**) fasting blood glucose levels, and (**G**) serum Ca^2+^ levels after 30 days of the indicated drug treatments (*n* = 3–6 mice per group). Data are shown as the mean ± SEM. *P < 0.05, **P < 0.005, ***P < 0.0005, and ****P < 0.0001, by 1-way ANOVA.

**Figure 10:**
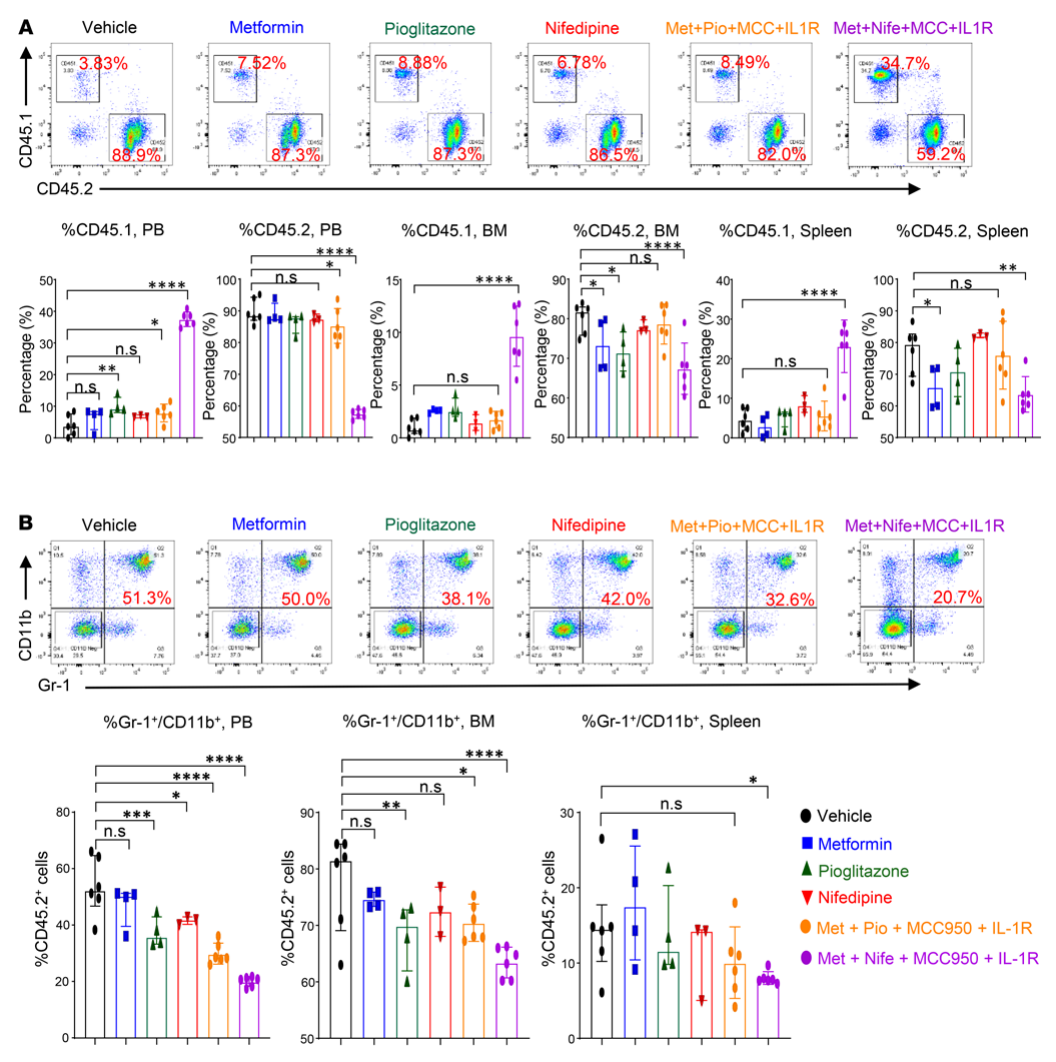
Effect of metformin, nifedipine, MCC950, and anakinra treatment on *Tet2*-deficient myeloid cells. (**A**) Representative flow cytometry plots of donor chimerism (CD45.1^+^/CD45.2^+^) in the PB of recipient mice after 30 days of the indicated drug treatment and quantification of CD45.1^+^ cells in the PB, CD45.2^+^ cells in the PB, CD45.1^+^ cells in the BM, CD45.2^+^ cells in the BM, CD45.1^+^ cells in the spleen, and CD45.2^+^ cells in the spleen after 30 days of the indicated drug treatment. (**B**) Representative flow cytometry profile of myeloid cells (Gr-1^+^CD11b^+^) in the PB of recipient mice after 30 days of the indicated drug treatment and quantification of Gr-1^+^CD11b^+^ double-positive cells in the PB, BM, and spleen after 30 days of the indicated drug treatment (*n* = 3–6 mice per group). Data are shown as the mean ± SEM. *P < 0.05, **P < 0.005, ***P < 0.0005, and ****P < 0.0001, by 1-way ANOVA.

**Figure 11:**
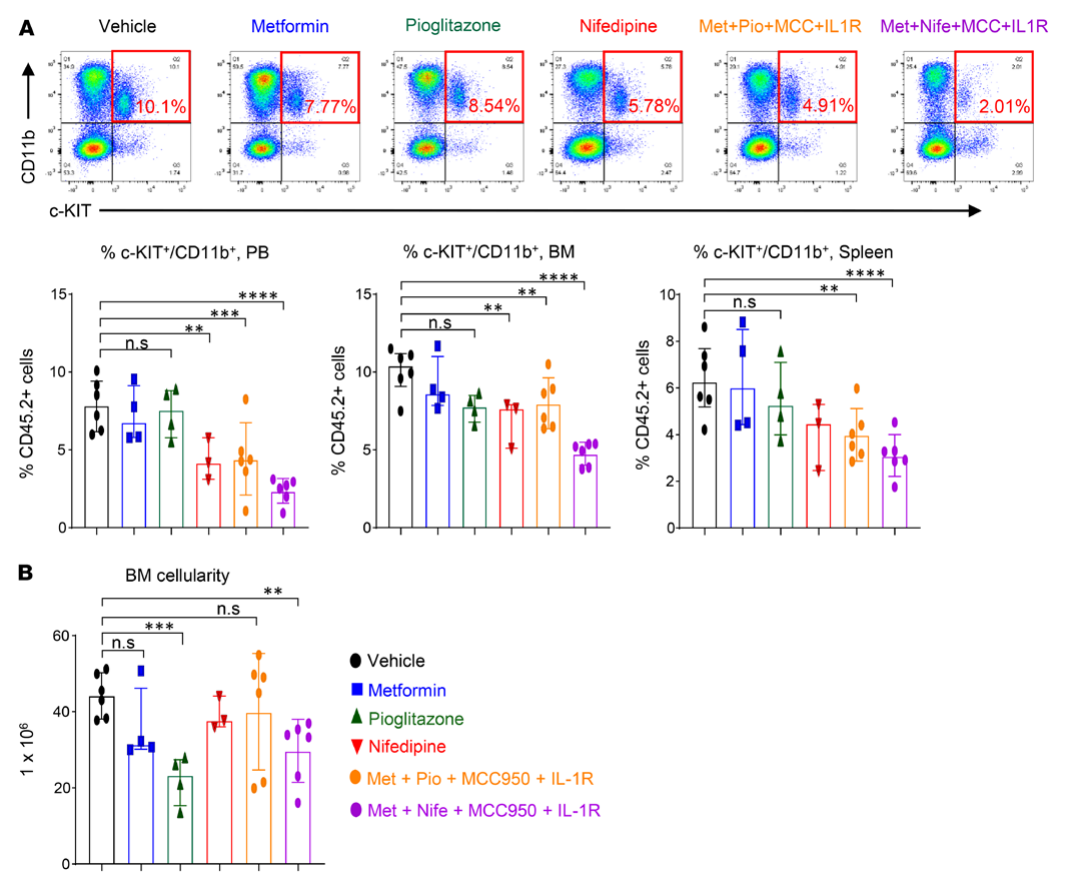
Effect of metformin, nifedipine, MCC950, and anakinra treatment on *Tet2*-deficient myeloid blasts and BM cellularity. (**A**) Representative flow cytometry profile of myeloid blasts (c-KIT^+^CD11b^+^ double-positive cells) in the PB of recipient mice after 30 days of drug treatment and quantification of c-KIT^+^CD11b^+^ double-positive cells in the PB, BM, and spleen. (**B**) BM cellularity after 30 days of the indicated drug treatment (*n* = 3–6 mice per group). Data are shown as the mean ± SEM. **P < 0.005, ***P < 0.0005, and ****P < 0.0001, by 1-way ANOVA.

Briefly, we performed a competitive BMT experiment using a mixture of *Tet2^–/–^* BM cells and age-matched Boy/J BM cells and transplanted them into *Ob/Ob* mice. Eight weeks later, we treated these mice with pharmacological inhibitors, either individually or in combination. Metformin (gluconeogenesis inhibitor; 100 mg/kg, oral) (52), pioglitazone (PPAR-γ inhibitor; 20 mg/kg, oral) (53), nifedipine (Ca^2+^ channel blocker; 100 μg/kg, oral) (40), MCC950 (Nlrp3 or IL-1β inhibitor; 30 mg/kg, oral) (54, 55), and anakinra (IL-1 receptor antagonist; 10 mg/kg, i.p, 10 days) (56) were administered individually or in combination for 30 days (Figure 8, A and B). As shown in Figure 8C and Supplemental Figure 10, all the single and combined drug treatment strategies reduced PB WBC, neutrophil, and monocyte counts in *Ob/Ob* mice bearing *Tet2^–/–^* cells. However, treatment with the combination of metformin+nifedipine+MCC950+anakinra demonstrated the most robust and durable reduction in both the frequency and percentage of monocytes, neutrophils, WBCs, and eosinophils and improved the lymphocyte, RBC, and platelet counts in *Ob/Ob* mice bearing *Tet2^–/–^* cells compared with mice in the other groups (Figure 8C and Supplemental Figure 11, A–F). The presence of CHIP-associated mutations in older individuals is associated with a higher RDW (>14.5%) (1, 31). Importantly, RDW values were significantly reduced in the above combination treatment in *Ob/Ob* mice bearing *Tet2^–/–^* cells compared with other groups (Supplemental Figure 11G). We found that the combination of metformin, pioglitazone, MCC950, and anakinra was equally effective at reducing RDW values and improving platelets counts. Spleen, liver, heart, and body weights as well as fasting blood glucose levels were all significantly reduced upon treatment of the mice with individual drugs as well as a combination of the drugs. However, the combination of metformin, nifedipine, MCC950, and anakinra treatment showed the most robust and durable reduction in *Ob/Ob* recipient mice transplanted with *Tet2^–/–^* cells compared with mice in the other groups (Figure 9, A–F, and Supplemental Figure 12, A–C). Moreover, serum Ca^2+^ levels (Figure 9G), *Irg1* gene expression levels (Supplemental Figure 12D), and global 5-hmC levels (Supplemental Figure 12E) were all modulated by single-drug treatment of these mice to a different extent, with the most efficient response seen with the combination drug treatment. Remarkably, we observed a greater reemergence of normal CD45.1^+^ WT (Boy/J) cells in the PB, BM, and spleens and a significant reduction in the presence of mutant *Tet2^–/–^* CD45.2^+^ pre-LHSCs/PCs after 30 days of combination drug treatment (Figure 10A). Furthermore, myeloid cells (Gr-1^+^CD11b^+^) in the PB, BM, and spleens were reduced upon single drug treatment, although the response was more robust in the combination drug treatment group (Figure 10B). The frequency of myeloid blasts (c-KIT^+^CD11b^+^ cells) in the PB, BM, and spleens was also reduced in *Ob/Ob* mice bearing *Tet2^–/–^* cells compared with the other groups. Again, we observed the most robust responses in mice treated with the combination of drugs, in particular metformin+nifedipine+MCC950+anakinra (Figure 11A). Abnormal BM cellularity (Figure 11B) and the absolute number of hematopoietic progenitors, including total LSK cells, HPC-1 cells, and LT-HSCs (Supplemental Figure 11, H–K) and the frequency of total GMPs in the BM (Supplemental Figure 11, L and M) were also partially reduced to different degrees by various drug treatments.

A pronounced myeloid shift is observed in both individuals with CHIP and in mouse models of CHIP, along with a concomitant decrease in lymphocytes (1, 57, 58). One possible mechanism to explain this phenomenon is that the elevated numbers of myeloid cells may result in the suppression of lymphocytes (59). Consistent with this notion, lymphocytes were reduced in *Ob/Ob* mice bearing *Tet2^–/–^* cells (Supplemental Figure 4F, Figure 5G, and Supplemental Figure 2, E and H). Remarkably, B cells (B220^+^) in the PB and BM of *Ob/Ob* mice transplanted with *Tet2^–/–^* cells were improved upon individual drug treatment but most profoundly with the combined drug treatment (Supplemental Figure 11, N and O). Likewise, all single drug treatments rescued the suppression of lymphocytes to a different extent in the PB and of CD4^+^ T cells in the spleen and BM of *Ob/Ob* mice bearing *Tet2^–/–^* cells compared with mice in the other groups (Supplemental Figure 11, D, P, and Q). In the context of CHIP, myeloid cells have been shown to be proinflammatory, and CHIP exhibits elevated levels of serum cytokines and chemokines (41, 57, 58). Serum cytokine and chemokine analysis of single-drug–treated *Ob/Ob* mice bearing *Tet2^–/–^* cells revealed a differential reduction in the levels of proinflammatory cytokines such as *Il1b*, *Tnfa*, *IL10*, *IL12p70*, *Il13*, and *GMCSF* and chemokines such as *CXCL1*, *CXCL9*, *CXCL10*, and *Rantes*; however, the most robust decline was noted in mice treated with the combination of metformin+nifedipine+MCC950+anakinra (Supplemental Figure 12F).

Given that more durable responses across the board were seen in mice treated with the combination of 4 drugs, in particular with the addition of the Ca^2+^ blocker nifedipine, we more closely examined the role of Ca^2+^ blockers in driving obesity-induced CH. Apart from voltage-gated channels, the store-operated calcium entry (SOCE) channel is a major mechanism for Ca^2+^ influx from the extracellular space into nonexcitable cells such as HSC/Ps (60). Therefore, we also examined the role of SOCE inhibitors in suppressing Tet2-mediated clonal expansion under conditions of obesity. *Ob/Ob* mice bearing *Tet2^–/–^* cells were treated with the known SOCE inhibitor SKF-96365 (61–63), alone or in combination with metformin, MCC950, and anakinra. As seen earlier in mice treated with metformin+nifedipine+MCC950+anakinra, we also observed that the SOCE inhibitor SKF-96365 in combination with metformin+MCC950+anakinra resulted in a significant reduction in WBCs, neutrophils, monocytes, and RDW percentage and improved the deficiencies in lymphocytes, RBCs, and platelets in *Ob/Ob* mice bearing *Tet2^–/–^* cells compared with the other groups (Supplemental Figure 13, A–G). Spleen weight, body weight, and blood glucose levels were also reduced with this combination treatment in *Ob/Ob* recipient mice transplanted with *Tet2^–/–^* HSC/Ps compared with controls (Supplemental Figure 13, H–J). Moreover, the combination treatment of metformin+SKF-96365+MCC950+anakinra also resulted in a reduction of *Tet2^–/–^*-mutant CD45.2 cells in the PB, with a concomitant reemergence of normal CD45.1^+^ WT cells in the PB and BM (Supplemental Figure 13, K–N). Abnormal BM cellularity, the frequencies of total LSK cells, HPC-1 cells, and GMPs were all reduced in the combination treatment group compared with controls (Supplemental Figure 13, O–S). Altogether, these results suggest that both VGCC and SOCE blockers in combination with metformin+MCC950+anakinra can affect CH.

### Affect of drug treatment on gene expression in Tet2^–/–^ cells from obese mice.

**Figure 12:**
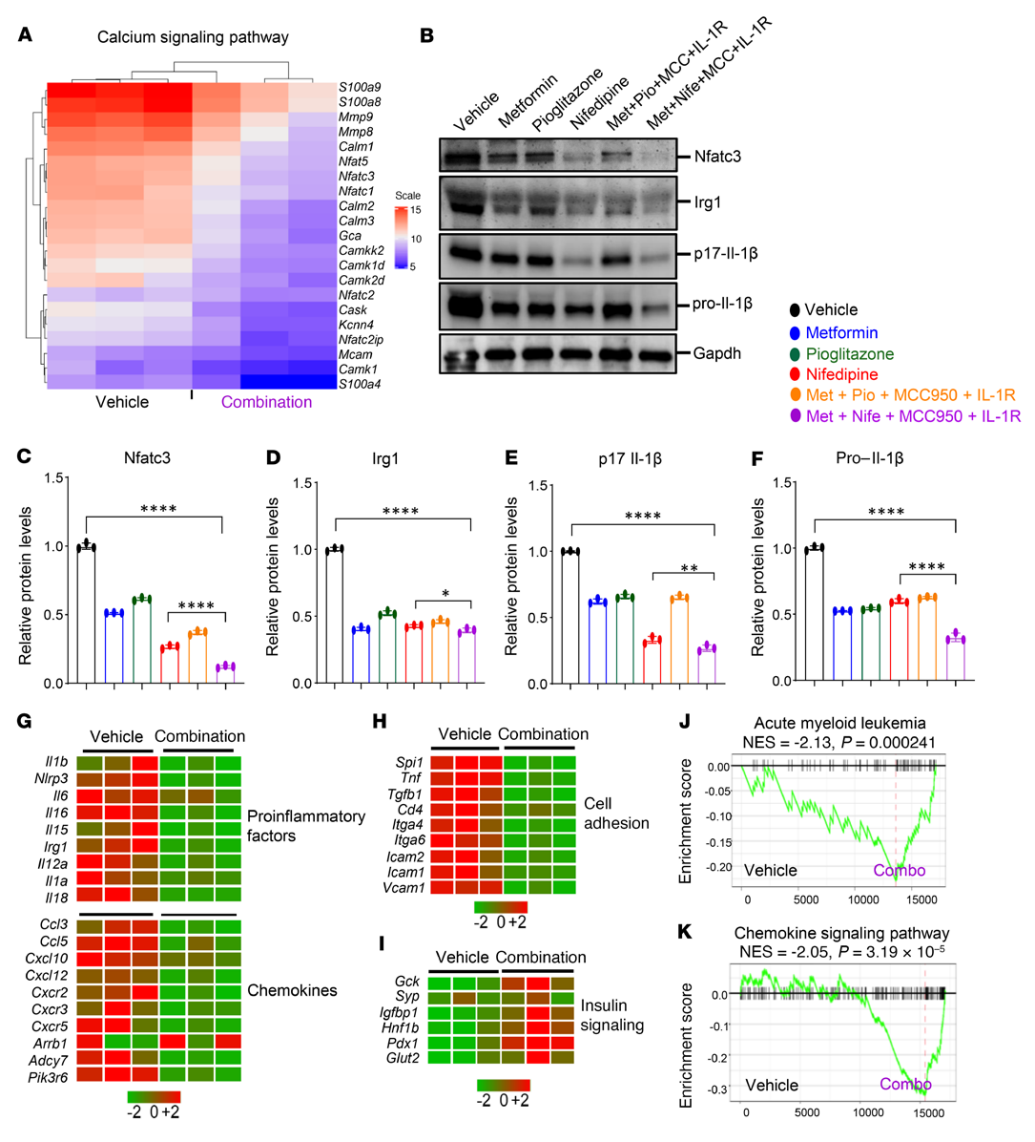
Effect of metformin, nifedipine, MCC950, and anakinra treatment on gene expression. (**A**) Heatmap of DEGs related to Ca^2+^ signaling pathway after 30 days of the indicated drug treatment. (**B**–**F**) WB analysis was performed to measure the protein levels of Nfatc3, Irg1, and Il-1β (p17 and pro–Il-1β) after 30 days of the indicated drug treatment. Data are shown as the mean ± SEM. *P < 0.05, **P < 0.005, and ****P < 0.0001, by 1-way ANOVA. (**G**–**I**) Heatmaps of DEGs of proinflammatory factors, chemokines, cell-adhesion molecules, and insulin signaling pathway genes after 30 days of the indicated drug treatment. (**J** and **K**) GSEA plots of gene sets related to AML (**J**) and the chemokine signaling pathway (**K**) after 30 days of the indicated drug treatment.

To assess the impact of drug treatment on gene expression in *Tet2^–/–^* cells under obesity conditions, total RNA was isolated from whole BM from drug-treated mice. We performed bulk RNA-Seq on whole BM samples from 3 individual mice. DEG and pathway enrichment analysis of the RNA-Seq data from the combined drug treatment group showed a reduction in the expression of genes involved in the calcium signaling pathway, including *Nfatc3*, *Nfatc2*, *Nfatc1*, *Nfat5*, *S100a8*, *S100a9*, *Camkk2*, *Mmp8*, *Mmp9*, and *Calm2* (Figure 12A). Furthermore, we also observed a reduction in the Ca^2+^/calcineurin-activated NFAT pathway and cytosolic Ca^2+^ levels in the drug-treated *Ob/Ob* mice bearing *Tet2^–/–^* cells compared with mice in the vehicle-treated group (Supplemental Figure 14, A and B). Additionally, WB analysis confirmed that Nfatc3, Irg1, and Il-1β (p17 and pro–Il-1β) protein levels were reduced to a different extent in the drug-treated mice, but the mice treated with the combination of metformin+nifedipine+MCC950+anakinra showed the greatest reduction in these protein levels (Figure 12, B–F). The combination drug treatment markedly inhibited the increased expression of proinflammatory factor genes including inflammasome (*Il1b*, *Il6*, *Il12*, *Il16*, *Irg1*, and *Nlrp3)*, chemokine, and cell adhesion genes (Figure 12, G and H). Moreover, GSEA of the combined drug treatment group revealed a negative enrichment of genes related to AML and the chemokine signaling pathway compared with the vehicle-treated group (Figure 12, J and K).

RNA-Seq analysis of cells independently treated with nifedipine and metformin also showed a significant correction in pathway genes for calcium signaling, cell cycle, peroxisome proliferator–activated receptor γ coactivator 1 α (PGC-1α) (aberrant increases in PGC-1α activity have been linked to heart failure and diabetes), glucose and mineral transport, alanine, and aspartate and glutamate metabolism compared with other groups (Supplemental Figure 14, C–H). Furthermore, individual drug treatment of obese mice bearing *Tet2^–/–^* pre-LHSCs/PCs demonstrated that these drugs targeted both similar and independent genes in CHIP mutation–bearing cells as depicted in the scatter plot analysis (Supplemental Figure 14, I and J). Moreover, drug combination treatment markedly reduced the expression of genes involved in diabetes and glucotoxicity pathways (Supplemental Figure 14K) and improved the expression of genes in the insulin signaling pathway, including pancreatic and duodenal homeobox 1 (*Pdx1*), glucose transporter 2 (*Glut2*), synaptopodin (*Syp*), glucokinase (*Gck*), and insulin-like growth factor–binding protein 1 (*Igfbp1*) compared with the vehicle-treated mice (Figure 12I).

## Discussion

CHIP represents a novel risk factor for the development of hematologic malignancy (1, 2). As CHIP is present in asymptomatic individuals and since not all individuals with CHIP develop these pathologies, it is critical to understand the factors that promote clonal expansion and disease progression. Common comorbidities such as obesity, diabetes, and infection, which promote proinflammatory environments, may play key roles in driving and accelerating CHIP-associated pathologies. To explore the possibility that obesity can exacerbate clonal expansion and CHIP-associated diseases, we investigated the relationship between obesity and CHIP in a cohort of individuals carrying CHIP-associated mutations and tested the biological consequences of this relationship in mouse models of CHIP-associated mutations in the context of obesity. We show that the presence of a CHIP-associated mutation was associated with a higher WHR and prevalence of CAD, hypertension, and hypercholesterolemia compared with a low WHR in a cohort of 47,466 unrelated participants who had valid CHIP measurements in the UK Biobank. Importantly, we observed that obesity (BMI, *n* = 46,460; WHR, *n* = 47,405) was associated with myeloid leukemia. In addition to the analysis of our own data described above, additional recent studies have shown a strong association between obesity and leukemia, including both myeloid and lymphoid types of leukemia (22, 23, 64–70). A number of publications have demonstrated a strong correlation between myeloid leukemias and obesity in both children and adults, with an increased risk and worse survival outcomes for obese individuals and/or overweight individuals (69, 71–75). More specifically, Larsson et al. (23), Kasim et al. (70), and Castillo et al. (22) noted an increased risk and/or incidence of both AML and chronic myeloid leukemia (CML) in obese individuals and/or overweight individuals (22, 23, 70). MacInnis et al. (76) showed that the risk of myeloid leukemia is associated BMI, fat-free mass, and waist circumference. Additionally, a relationship between increased BMI and acute promyelocytic leukemia (APL), a subset of AML, has been identified (76). Furthermore, in patients with APL, a higher BMI is associated with complications including thrombohemorrhagic early death and a higher risk of relapse and differentiation syndrome, as well as increased treatment-related mortality (64, 77). In the CCG-2961 clinical trial for pediatric AML, obesity was identified as a predictive factor for inferior survival rates (78). Patients with promyelocytic leukemia also exhibit upregulation of genes involved in polyunsaturated fatty acid metabolism, and individuals with de novo APL have more frequently have a history of hyperlipidemia as well as higher median BMIs and incidence of obesity, further supporting a potential pathogenic link between obesity and myeloid leukemias (64, 79). Together, these studies show that obesity is linked to worse outcomes for patients with leukemia, including myeloid leukemia, thus highlighting the clinical relevance of obesity in these patient populations. As CHIP-associated mutations have been detected in both myeloid and lymphoid malignancies and coincide with a pre-leukemic state, our findings of increased CHIP-associated mutations in individuals with obesity are consistent with these studies demonstrating a correlation between obesity and leukemia. Moreover, our TCGA data analysis revealed that the mutation rate of CHIP-associated genes was higher in patients with a high BMI (>30 kg/m^2^) compared with those with a low BMI (≤25 kg/m^2^) in 6 different cancer types. These data support previous studies that CHIP may also contribute to disease progression in solid tumors and therapy-related neoplasms and suggest that obesity may be an important risk factor in these patient populations (6, 80–84).

Our experimental studies in mice corroborate the observations in the patient cohort, providing a proof of concept that obesity-induced changes can promote the expansion of pre-leukemic CHIP to a severe MPN-like phenotype in mouse models using several CHIP-associated genes including *Tet2*, *Dnmt3a*, *Asxl1*, and *Jak2*. The increased clonal expansion and the development of a MPN-like phenotype in all of these models suggest that obesity may play a universal role in catalyzing or exacerbating CHIP-associated disease, regardless of the type of mutation. Our results from a competitive transplantation assay provide evidence that the significant differences in CHIP-mutant pre-LHSCs/PCs (CD45.2^+^ cells) in obese FBM recipients support their competitive advantage, clonal expansion, and subsequent malignancy compared with WT HSC/Ps. Further, these mice also had a higher frequency of myeloid cells and myeloid blasts in the PB, BM, and spleen and of HSC/PC populations such as LSK cells, HPC-1 cells, and GMPs in the BM compared with control mice. Importantly, these mice also had elevated fasting blood glucose levels and increased heart weights compared with control mice.

The competitive selection of HSC/Ps depends on both somatic mutations and environmental factors. We and others have recently shown that LPS or microbial exposure of *Tet2*-deficient mice induces a strong inflammatory response (2, 13), thus indicating that the interaction between pre-leukemic mutations and environmental stressors drives CH. Consistently, all of the *Ob/Ob* FBM-recipient mice transplanted with BM bearing CHIP mutations showed significant increases in proinflammatory cytokines such as IL-6, IL-1β, and TNF-α compared with WT lean BM recipient mice. Recent reports demonstrate that aggravated IR in conditions of TET2 loss-of-function in HSCs is primarily facilitated by exacerbated IL-1β expression in aged and high-fat/high-sucrose (HF/HS) obesogenic diet–fed mice. Blockade of NLRP3 inflammasome–mediated secretion of IL-1β in TET2-deficient immune cells using MCC950 suppressed the higher levels of IL-1β and increased hyperglycemia and IR in mice carrying TET2-deficient HSCs (11). Furthermore, combined treatment with anakinra (IL-1 receptor antagonist) and MCC950 suppressed MAPK and Nlrp3-caspase via inhibition of the NF-κB pathway to improve LPS- and mechanical ventilation-induced acute lung injury inflammation in vivo compared with treatment with anakinra or MCC950 alone (85).

To better understand the mechanism by which FBM from obese mice promotes clonal expansion and CHIP-associated diseases, we investigated the role of several pathways that were modulated in *Ob/Ob* mice bearing *Tet2^–/–^* cells. In *Tet2^–/–^* *Ob/Ob* mice and chimeric *Ob/Ob* FBM mice transplanted with *Tet2^–/–^* HSC/Ps, we demonstrated that highly impaired expression of calcium signaling in *Tet2^–/–^* HSC/Ps might be one of the contributors to the development of CHIP-associated pathologies. We also found that aberrant Ca^2+^/calcineurin induced *Nfat* and *Irg1* signaling in *Ob/Ob* FBM recipient mice transplanted with *Tet2^–/–^* HSC/Ps, which has been shown, compared with controls, to result in the accumulation of extensive amounts of itaconate (86), a metabolite that plays a key role in restoring the TCA cycle and inhibits succinate dehydrogenase (SDH) (87). Furthermore, obesity-induced aberrant glucose availability can cause higher levels of acetyl-CoA, which frequently increases the influx of Ca^2+^ into the cells, leading to the activation and translocation of NFAT into the nucleus. This results in the recruitment of lysine acetyltransferase (KAT) p300 to drive the site-specific regulation of H3K27ac and results in overexpression of the *Irg1* gene, which can lead to the accumulation of itaconate. Itaconate binds to the same site as α-KG in TET enzyme and inhibits its catalytic activity during active DNA demethylation (42, 45), signifying a potential link between intracellular metabolism and epigenetic modifications. Consistent with these findings, we show that transgenic *Tet2^–/–^* *Ob/Ob* compound mutant mice and chimeric *Ob/Ob* FBM recipient mice transplanted with *Tet2^–/–^* HSC/Ps exhibited increased Ca^2+^ flux and a further reduction of global 5-hmC levels in their HSC/Ps compared with controls. These data demonstrate that increased levels of Ca^2+^, *Nfat*, and *Irg1* cooperated in the obesity-induced inflammation, expansion, and transformation of *Tet2^–/–^* pre-LHSCs/PCs.

Recent studies reported that SARS-CoV-2–infected individuals with obesity, dyslipidemia, or metabolic dysregulation have a higher risk of acute respiratory distress syndrome (ARDS), an inflammatory condition with high mortality rates (88), but that ARDS and mortality rates were considerably reduced by long-term metformin usage (89). Metformin treatment prevents LPS-induced ARDS in mice by inhibiting Nlrp3 inflammasome activation and IL-1β and IL-6 secretion (90). There is increasing evidence to suggest that metformin, a widely used antidiabetes drug, is also a potential anticancer agent (91, 92). Prior studies showed that abnormal activation of Ca^2+^ channels plays a key role in tumor progression and development, and recent findings showed that nifedipine, a dihydropyridine, L-type calcium channel blocker that inhibits Ca^2+^ influx and is also widely used to treat hypertension, effectively inhibits colorectal cancer progression (40). Given that we detected elevated levels of intracellular Ca^2+^, blood glucose, and IL-1β inflammatory cytokines in *Ob/Ob* FBM recipient mice transplanted with *Tet2^–/–^* HSC/Ps compared with controls, we evaluated the therapeutic efficacy of targeting these pathways in *Tet2^–/–^* *Ob/Ob* mice. We treated *Ob/Ob* mice bearing *Tet2^–/–^* pre-LHSCs/PCs with metformin, pioglitazone, nifedipine, or SKF-96365, MCC950, and anakinra, either individually or in combination. The efficacy of the combination treatment compared with the other treatments suggests that the inhibition of multiple signaling pathways and environmental stimuli may be needed to suppress CH, at least in the context of obesity. Our single-drug treatments using metformin and pioglitazone in *Tet2^–/–^* *Ob/Ob* mice also led to a decrease in Ca^2+^ levels as well as a decrease in *Irg1* expression. While one would expect to see this response in nifedipine-treated mice only, both metformin and pioglitazone have been shown to inhibit Ca^2+^ signaling to different degrees (93–95). Likewise, while metformin and pioglitazone are considered potent inhibitors of glucose, nifedipine, a calcium channel blocker, also lowers glucose levels in diabetic mice (96). Collectively, these prior studies help explain the reduction in Ca^2+^ levels, *Irg1* expression, and glucose levels observed in our studies involving single-drug treatment in *Tet2^–/–^* *Ob/Ob* mice. We believe that the impact of the drugs described in our study on the repression of CH is likely to be overlapping as well as distinct, with some drugs being more effective at repressing the growth of certain mutant hematopoietic lineages than others. Therefore, the combined effect of these drugs is more robust at inhibiting CH and thus yields stronger and more durable responses, as shown in our studies.

In summary, human genetic studies and murine models demonstrated that CHIP mutations were associated with obesity and that targeting CHIP-mutant cells with drugs such as metformin, nifedipine, MCC950, or anakinra may be a reasonable approach to treat CH. The association of obesity with CHIP-associated mutations has important implications for the identification of at-risk individuals and patient stratification. Collectively, these results indicate that obesity may be an important risk factor for the development and exacerbation of CHIP-associated diseases and propose a potential therapeutic approach to prevent these CHIP pathologies, at least in individuals with obesity.

## Methods

Detailed methods are provided in the supplemental material. A complete list of antibodies and reagents is provided in Supplemental Table 3.

### Data availability.

RNA-Seq data have been deposited in the NCBI’s Gene Expression Omnibus (GEO) database (GEO GSE193062).

### Statistics.

Statistical analysis was performed using GraphPad Prism 7 (GraphPad Software). One-way ANOVA with an uncorrected Fisher’s test or 2-tailed Student’s *t* test was used to determine significant differences between groups. A *P* value of less than 0.05 was considered significant. Data in figures are presented as mean ± SEM.

### Study approval.

The mouse studies were approved by the Indiana University Laboratory Animal Resource Center (Indianapolis, Indiana, USA), and all experiments were conducted at the Laboratory Animal Resource Center according to the protocol.

## Author contributions

SKP and R Kapur conceived the study and designed experiments. SKP and BR designed and executed the experiments and analyzed the data. SSB, R Kanumuri, R Kumar, and TP assisted with the experiments. ZY, MH, AB, GKG, AN, BLE, and PN provided human clinical and exome sequence data from 50,000 individuals with valid CHIP in the UK Biobank. LRP, NKY, XZ, and CZ analyzed the RNA-Seq data. GES performed histopathological analysis. SKP, SSB, and R Kapur wrote the manuscript. SP conceptualized the study. UD read the manuscript and provided critical input. All authors read and approved the manuscript.

## Supplementary Material

Supplemental data

Supplemental table 4

Supplemental table 5

## Figures and Tables

**Table 1:**
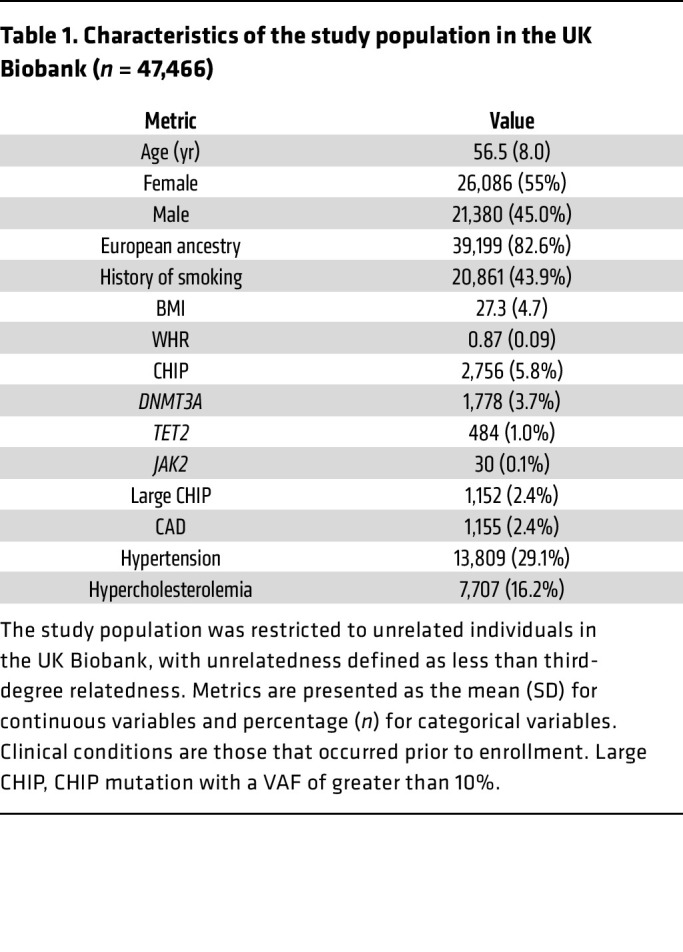
Characteristics of the study population in the UK Biobank (*n* = 47,466)

**Table 2:**
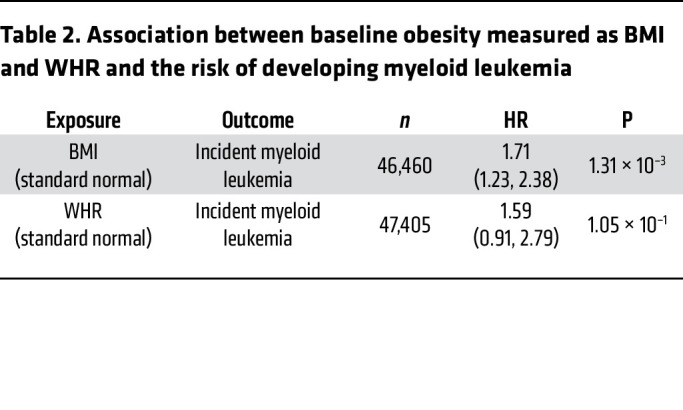
Association between baseline obesity measured as BMI and WHR and the risk of developing myeloid leukemia

## Version 1 04/18/2023

In-Press Preview

## Version 2 06/01/2023

Electronic publication
