# Supplementary material for: Obesity-induced inflammation exacerbates clonal hematopoiesis
Source: J Clin Invest. 2023 Jun 1;133(11):e163968. doi: 10.1172/JCI163968 (PMC10231999; doi:10.1172/JCI163968)
Supplement: Supplemental data [file jci-133-163968-s045.pdf]

Methods

Study population

The UK Biobank is a large, ongoing population-based cohort. Between 2006 and 2010, approximately 500,000 adult participants aged 40-69 years were recruited by one of 22 assessment centers across the United Kingdom. At baseline, participants provided a range of information, including detailed demographics and medical history, through questionnaires and interviews; anthropometric measures and blood samples were also taken. Details regarding this cohort have been described elsewhere (1). In the current analysis, we included 47,466 participants who were free of type 2 diabetes at baseline and had available whole exome sequences. We restricted to unrelated individuals where relatedness was defined as one individual in each pair within the third degree of relatedness determined based on kinship coefficients centrally calculated by UK Biobank (2). Access to UK Biobank data analyzed in this analysis can be applied for by researchers through https://www.ukbiobank.ac.uk/.

Whole exome sequencing and CHIP detection

Exomes of approximately 50,000 UK Biobank participants were sequenced from their whole blood cell-derived DNA as reported previously (3). CHIP detection was conducted using GATK Mutect2 software (https://software.broadinstitute.org/gatk) for detecting putative somatic SNPs and short indels in pre-specified list of variants in *DNMT3A* and *TET2* reported as driver mutations of myeloid malignancies in the literature and/or the Catalog of Somatic Mutations in Cancer (COSMIC; http://cancer.sanger.ac.uk/cancergenome/projects/cosmic) (Supplemental Table X) (4). Common sequencing artifacts and germline variants were excluded as described elsewhere (5).

Ascertainment of outcomes

Anthropometric measures were collected and recorded by trained nurses at baseline. Body weight was measured with a Tanita BC-418MA bio-impedance analyzer. Standing height was measured using a Seca height measure. Body mass index (BMI) was calculated as weight in kilograms divided by height in meters squared. Waist and hip circumferences were measured with a Seca 200 measuring tape at the level of the umbilicus and the widest part over the buttocks, respectively. Waist-to-hip ratio (WHR) was calculated by dividing the waist measurement by the hip measurement. Sex-specific quintiles of BMI and WHR were generated for this analysis.

Statistical analysis

Associations of CHIP with BMI and WHR were obtained using linear regression, with adjusting for age and age squared at recruitment, sex, current smoker status, genotyping array and the first 10 principal components of ancestry. We also examined the prevalence of CHIP mutation by sex-specific quintiles of BMI and WHR. All statistical analyses were conducted using R version 3.6.0. This research has been conducted using the UK Biobank Resource under Application number (7089).

Mice

All mice used for these studies were a mix of male and female on a C57BL/6J background. All mice were bred and maintained under specified pathogen-free conditions in the animal facility at Indiana University School of Medicine with 12-hr light/dark cycle and were provided food and water ad libitum. All the mouse experiments were approved and maintained by Laboratory Animal Resource Center at Indiana University School of Medicine. $Lep^{Ob/Ob}$ (*Ob/Ob*) mice were obtained from Jackson Laboratory (Stock No: 000632), $Tet2^{+/-}$, $Tet2^{-/-}$, $Dnmt3a^{+/-}$;Mx-Cre^{+}, $Asxl1^{+/-}$;Mx-Cre^{+}, $Jak2^{V6117F/+}$;Vav-Cre^{+} mice were obtained from our in-house stock and C57/B6 mice were procured from IUSM core facility and used as WT controls. To obtain leptin deficient (*Ob/Ob*) on

a background of Tet2 deficiency, *Ob*$^{+/-}$ mice were crossed with *Tet2*$^{-/-}$ mice, and the F1 progeny of these matings (*Tet2*$^{+/-}$;*Ob*$^{+/-}$) were then crossed to obtain mice that had either zero, one, or both normal *Tet2* alleles and were leptin-deficient (*Tet2*$^{-/-}$;*Ob/Ob*, *Tet2*$^{+/-}$;*Ob/Ob*, and *Tet2*$^{+/+}$;*Ob/Ob*, respectively) as well as control *Tet2*$^{-/-}$, *Tet2*$^{+/-}$, and WT mice. Similarly, *Ob*$^{+/-}$ mice were crossed with *Dnmt3a*$^{+/-}$;Mx-Cre^{+} mice to generate *Dnmt3a*$^{+/-}$;Mx-Cre^{+};*Ob/Ob* compound mutant mice and all of the controls. 11-months-old *Tet2*$^{-/-}$;*Ob/Ob* and *Dnmt3a*$^{+/-}$;*Mx-Cre*$^{+}$;*Ob/Ob* compound mutant mice and age matched control mice were used in the study.

Competitive bone marrow transplantation

Recipient animals (12-weeks-old *Ob/Ob* and WT) were lethally irradiated (700 cGy + 400 cGy) one day prior to transplantation (intravenous tail injection) of donor cells. The CD45.2^{+} donor BM cells from *Tet2*$^{-/-}$, *Dnmt3a*$^{+/-}$;Mx-Cre^{+}, *Asxl1*$^{+/-}$;Mx-Cre^{+}, *Jak2*$^{V617F/+}$;Vav-Cre^{+} mice (all of these donor mice were 11-months-old) were mixed individually with age matched Boy/J CD45.1^{+} competitor BM donor cells (a total of 1 x 10^{6} cells with an equal number of viable total BM cells, 500K:500K). For 1:10 BM transplantation, 10% CD45.2^{+} BM cells from 11-months-old *Tet2*$^{+/-}$ mice were mixed with 90% CD45.1^{+} BM cells from age matched Boy/J competitor mice and transplanted into lethally irradiated *Ob/Ob* or WT recipients (12-weeks-old). Chimerism analysis for progressive engraftment was analyzed on PB samples at 4-week intervals post-BM transplantation (6, 7).

Flow cytometry analysis

Immunophenotyping was performed as described previously (8). Single-cell suspensions of BM and spleen were prepared. Briefly, the BM and spleen cells were lysed in an RBC lysis buffer and resuspended in phosphate buffered saline (PBS) containing 0.2% bovine serum albumin (BSA) (Sigma) and 10% Fetal bovine serum (Sigma). Fluorochrome-conjugated antibodies specific to mouse Gr-1 APC/Cy7, CD11b PE, CD3 PE/Cy7 and B220 PerCp/Cy5.5 were used for detecting

mature myeloid cells. A panel of antibodies containing c-Kit APC, CD11b PE were used for myeloid blast cells labeling. A panel of antibodies containing Lin-PE cocktail (TER-119, Gr1, CD11b, B220 and CD3), c-Kit APC, Sca-1 PE/Cy7, CD48 APC/Cy7 and CD150 PerCp/Cy5.5 were used for LSK/HSC labeling. A panel of antibodies containing Lin-PE cocktail, c-Kit APC/Cy7, Sca-1 PerCp/Cy5.5, CD16/32 PE/Cy7, CD34 BV421 and CD127 APC were used for progenitors (CMP/GMP/MEP/CLP) labeling. For chimerism analysis of mature cells in PB, BM and spleen, CD45.1 APC and CD45.2 FITC were mixed with CD11b PE, Gr-1 APC/Cy7, CD3 PE/Cy7 and B220 PerCp/Cy5.5. For chimerism analysis in LSK/HSCs, CD45.1 APC and CD45.2 FITC were mixed with Lin-PE cocktail, c-Kit BV785, Sca-1 PE/Cy7, CD48 APC/Cy7 and CD150 PerCp/Cy5.5. For chimerism analysis in progenitors (CMP/GMP/MEP/CLP), CD45.1 APC and CD45.2 FITC were mixed with Lin-PE cocktail, c-Kit BV785, Sca-1 PE/Cy7, CD16/32 APC/Cy7 and CD34 BV421. For chimerism analysis in myeloid blasts, CD45.1 APC and CD45.2 FITC were mixed with c-Kit APC/Cy7 and CD11b PE. For chimerism analysis in cytotoxic T-cells, CD45.1-PE-CF594 and CD45.2-PB were mixed with CD3 FITC, CD4 APC and CD8 PerCp/Cy5.5. Multiparameter analysis of stained single-cell suspensions of BM and spleen were subjected to flow cytometric analysis using FASC Canto, LSR-II and LSR Fortessa flow cytometers with diva software (BD biosciences) and the data was analyzed using FlowJo software (v10.7.0) (6, 8, 9). List of antibodies and reagents are provided in **Supplemental Table 3**.

In vivo therapeutic interventions

The CD45.2^{+} donor BM cells from *Tet2*$^{-/-}$ mice were mixed with age matched Boy/J CD45.1^{+} competitor BM donor cells (with an equal number of viable total BM cells, 500K:500K). Recipient mice (*Ob/Ob* and WT) were lethally irradiated (700 cGy + 400 cGy) one day prior to transplantation (intravenous tail injection) of donor cells. At 8 weeks post-transplantation, the *Ob/Ob* recipient mice were treated with these mice with pharmacological inhibitors, either individually, or in combination. Metformin (Cat no: S1950, Selleckchem) (10), (Cat no: HY-13956,

MedChemExpress) (11), nifedipine (Cat no: Ab120135, Abcam) (12), SKF-96365 (Cat no: HY-100001, MedChemExpress) (13–15) MCC950 (Cat no: S7809, Selleckchem) (16, 17) and anakinra (Cat no: HY-108841, MedChemExpress) (18) were administered individually or in combination for 30 days.

Peripheral blood counts

Total white blood cell counts in freshly collected mouse blood were performed using hematology cell counter (HT5 Element) (6).

5-hmC quantification

DNA was extracted from whole BM samples from mice in the aforementioned seven treatment groups, and 5-hmC levels were quantified per the manufacturer's protocol (Quest 5-hmC™ DNA ELISA Kit (Cat no: D5425, ZYMO research) (19).

Serum cytokine analysis

Serum was separated from peripheral blood and subjected to mouse serum cytokine/chemokine (array MD31-plex) analysis through Eve Technologies (Canada). Results are expressed as pg/mL of serum (6).

Assessment of systemic metabolism

Fasting blood glucose levels were measured with an Accu-Chek glucometer (Roche Diagnostics) (20).

Quantitative real-time PCR

RNA was isolated from BM cells using the RNeasy kit (Qiagen) and reverse transcribed with the SuperScript™ VILO™ cDNA Synthesis Kit (ThermoFisher Scientific). qPCR was performed using the TaqMan™ Fast Advanced Master Mix (Applied Biosystems) in an Applied Biosystems Real-

time PCR system. The primers used were: *Irg1*: Mm01224531_m1 (ThermoFisher), *Nfatc3*: Mm01249200_m1 and *Gapdh* (Mm99999915_g1) was employed as a reference gene. Results were analyzed with the $^{\Delta\Delta}$Ct method.

Western Blot analysis

Protein extracts were obtained from BM samples using ice-cold lysis buffer supplemented with protease and phosphatase inhibitors. Equal amount of protein extracts was separated on 4-20% SDS-polyacrylamide gels and analyzed by Western Blot (NCM membranes).

RNA sequencing, differential gene expression, and pathway enrichment analysis

The RNA samples were processed and sequenced at the Center for Medical Genomics, IUSM as described previously (21). Differential gene expression analysis was conducted by using DESeq2 [25516281] with $p<0.005$ as the significant cut-off (22). Pathway enrichment (PE) analysis was led by hypergeometric tests against mouse Gene Ontology and MsigDB v6 canonical pathways, with p value less than 0.001 as the significant cutoff. For selected pathways, gene set enrichment analysis (GSEA) enrichment score and ssGSEA sample-wise enrichment score were used for visualization (23). ClueGo analysis was performed on the cytoscape V.3.9.1 software platform as mentioned earlier (24, 25). The Protein-protein interaction (PPI) maps were further subjected to clustering analysis using Molecular Complex Detection (MCODE) application (26), followed by CytoHubba analysis (27) in the cystoscape environment. The RNA-seq raw data was submitted to the NCBI Gene Expression Omnibus (GEO) database (accession number GSE193062).

Measurement of intracellular $[Ca2^+]_i$ levels

Intracellular flow cytometry was performed to detect the expression of $Ca2^+$ levels in HSC/Ps. Freshly prepared BM cells were pre-stained using cell-surface antibodies for HSC/Ps (LSK and/or progenitors). The pre-stained cells were loaded with Calbryte 520 AM dye (AAT Bioquest, Sunnyvale CA; Cat. No. 36310), a FITC-conjugated fluorophore, following the manufacturer's

protocol. After incubation for 35 min, the LSK and/or progenitors were subjected to flow cytometric analysis using LSRFortessa flow cytometer with diva software (BD biosciences) maintained at 37°C. After allowing the baseline to settle, 1μM ionomycin was added to induce calcium transients. Relative changes in $[Ca2^+]_i$ were read using the kinetic reading mode at Ex/Em 490/525 nm. FlowJo v10.7.0 software (BD Biosciences) was used to analyze the kinetics data (28).

Confocal microscopy

For nuclear translocation of *Nfatc3* expression, *Tet2*$^{-/-}$;*Ob/Ob*, *Tet2*$^{-/-}$, *Ob/Ob* and WT HSC/Ps were grown on ibidi µ-slide 8 well™ slides (Ibidi, Munich, Germany) and incubated with or without 1µm ionomycin for 2 hours according to the manufacturer's instructions. Further, these cells were fixed with 4% para formaldehyde at room temperature for 15 minutes and blocked with 5% UltraCruz® blocking reagent (sc-516214) before being incubated 90 min with *Nfatc3* primary antibody conjugated to Alexa Fluor® 488 (sc-8405 AF488). The slides were washed with three changes of PBS for 5 min each, and stain with DAPI for 1 min, and wash the slides with PBS. Images were taken with an Olympus Fluoview 1000 MPE confocal microscope system in Indiana University microscope core facility, and 4-5 fields/condition were analyzed with (Fiji) ImageJ.

TCGA data analysis of CHIP genes in high BMI and low BMI patients in 6 different cancer types

We collected the BMI information from the Cancer Genome Atlas (TCGA) data in six cancer types, namely bladder cancer (n=345), cervical squamous cell carcinoma (n=237), colon cancer (n=223), rectal cancer (n=65), uterine corpus endometrial cancer (n=375) and melanoma (n=140) (29). We matched BMI information and mutation information in the six cancer types and checked the mutation rate of CHIP genes in the samples of high BMI and low BMI in each cancer type and tested the statistical significance using fisher exact test.

References

1. Collins R. What makes UK Biobank special?. *Lancet* 2012;379(9822):1173–1174.

2. Bycroft C et al. The UK Biobank resource with deep phenotyping and genomic data. *Nature* 2018;562(7726):203–209.

3. Van Hout C V. et al. Exome sequencing and characterization of 49,960 individuals in the UK Biobank. *Nature* 2020;586(7831):749–756.

4. Cibulskis K et al. Sensitive detection of somatic point mutations in impure and heterogeneous cancer samples. *Nat. Biotechnol.* 2013;31(3):213–219.

5. Bick AG et al. Genetic Interleukin 6 Signaling Deficiency Attenuates Cardiovascular Risk in Clonal Hematopoiesis. *Circulation* 2020;124–131.

6. Ramdas B et al. Driver Mutations in Leukemia Promote Disease Pathogenesis through a Combination of Cell-Autonomous and Niche Modulation. *Stem Cell Reports.* 2020;15(1):95–109.

7. Cai Z et al. Targeting Bim via a lncRNA Morrbid Regulates the Survival of Preleukemic and Leukemic Cells. *Cell Rep.* 2020;31(12):107816.

8. Cai Z et al. Inhibition of Inflammatory Signaling in Tet2 Mutant Preleukemic Cells Mitigates Stress-Induced Abnormalities and Clonal Hematopoiesis. *Cell Stem Cell* 2018;23(6):833–849.

9. Cai Z et al. Hyperglycemia cooperates with Tet2 heterozygosity to induce leukemia driven by proinflammatory cytokine-induced lncRNA Morrbid. *J. Clin. Invest.* 2021;131(1):e140707.

10. Zhou ZY et al. Metformin exerts glucose-lowering action in high-fat fed mice via attenuating endotoxemia and enhancing insulin signaling. *Acta Pharmacol. Sin.* 2016;37(8):1063–1075.

11. Lam YY, Tsai SF, Chen PC, Kuo YM, Chen YW. Pioglitazone rescues high-fat diet-induced

depression-like phenotypes and hippocampal astrocytic deficits in mice [Internet]. *Biomed. Pharmacother.* 2021;140(February):111734.

12. Wu L et al. Calcium Channel Blocker Nifedipine Suppresses Colorectal Cancer Progression and Immune Escape by Preventing NFAT2 Nuclear Translocation. *Cell Rep.* 2020;33(4):108327.

13. Mai X et al. Blockade of orai1 store-operated calcium entry protects against renal fibrosis. *J. Am. Soc. Nephrol.* 2016;27(10):3063–3078.

14. Jing Z et al. SKF-96365 activates cytoprotective autophagy to delay apoptosis in colorectal cancer cells through inhibition of the calcium/CaMKIIγ/AKT-mediated pathway [Internet]. *Cancer Lett.* 2016;372(2):226–238.

15. Liang X, Zhang N, Pan H, Xie J, Han W. Development of Store-Operated Calcium Entry-Targeted Compounds in Cancer. *Front. Pharmacol.* 2021;12(May):1–18.

16. Wang L, Lei W, Zhang S, Yao L. MCC950, a NLRP3 inhibitor, ameliorates lipopolysaccharide-induced lung inflammation in mice. *Bioorganic Med. Chem.* 2021;30:115954.

17. Perera AP et al. MCC950, a specific small molecule inhibitor of NLRP3 inflammasome attenuates colonic inflammation in spontaneous colitis mice. *Sci. Rep.* 2018;8(1):1–15.

18. De Luca A et al. IL-1 receptor blockade restores autophagy and reduces inflammation in chronic granulomatous disease in mice and in humans. *Proc. Natl. Acad. Sci. U. S. A.* 2014;111(9):3526–3531.

19. Gilat N et al. Single-molecule quantification of 5-hydroxymethylcytosine for diagnosis of blood and colon cancers. *Clin. Epigenetics* 2017;9(1):1–8.

20. Nagareddy PR et al. Adipose tissue macrophages promote myelopoiesis and monocytosis in obesity. *Cell Metab.* 2014;19(5):821–835.

21. Pandey R et al. SHP2 inhibition reduces leukemogenesis in models of combined genetic and epigenetic mutations. *J. Clin. Invest.* 2019;129(12):5468–5473.

22. Love MI, Huber W, Anders S. Moderated estimation of fold change and dispersion for RNA-seq data with DESeq2. *Genome Biol.* 2014;15(12):1–21.

23. Subramanian A et al. Gene set enrichment analysis: A knowledge-based approach for interpreting genome-wide expression profiles. *Proc. Natl. Acad. Sci. U. S. A.* 2005;102(43):15545–15550.

24. Bindea G et al. ClueGO: A Cytoscape plug-in to decipher functionally grouped gene ontology and pathway annotation networks. *Bioinformatics* 2009;25(8):1091–1093.

25. Shannon P, Markiel A, Owen Ozier, Nitin S. Baliga, Jonathan T. Wang DR, Amin N, Benno Schwikowski TI. Cytoscape: A Software Environment for Integrated Models [Internet]. *Genome Res.* 2003;13(22):2498–2504.

26. Gary D Bader CWH. An automated method for finding molecular complexes in large protein interaction networks. *BMC Bioinformatics* 2003;4(2).

27. Chin CH et al. cytoHubba: Identifying hub objects and sub-networks from complex interactome. *BMC Syst. Biol.* 2014;8(4):1–7.

28. Chaudhuri P, Smith AH, Graham LM, Rosenbaum MA. Inhibition of P110a and P110d catalytic subunits of PI3 kinase reverses impaired arterial healing after injury in hypercholesterolemic male mice. *Am. J. Physiol. - Cell Physiol.* 2021;320(6):C943–C955.

29. Hu C et al. Body mass index-associated molecular characteristics involved in tumor immune and metabolic pathways. *Cancer Metab.* 2020;8(1):1–14.

Supplemental Table 1. Prevalence of CHIP across BMI (N=46460) and WHR (N=47405) status.

Exposure	Outcome	N	Beta	SE	P
**CHIP**	BMI	46460	0.030	0.093	7.43E-01
**Large CHIP (VAF>10%)**		46460	0.204	0.141	1.48E-01
TET2		46460	0.433	0.215	4.45E-02
DNMT3A		46460	-0.156	0.114	1.69E-01
**CHIP**	WHR	47405	0.003	0.001	3.39E-02
**Large CHIP (VAF>10%)**		47405	0.002	0.002	3.55E-01
TET2		47405	0.007	0.003	3.12E-02
DNMT3A		47405	0.000	0.002	9.32E-01
**CHIP**	BMI (standardized to 0-mean and unit-variance and normalized)	46460	0.006	0.020	7.43E-01
**Large CHIP (VAF>10%)**		46460	0.043	0.030	1.48E-01
TET2		46460	0.092	0.046	4.45E-02
DNMT3A		46460	-0.033	0.024	1.69E-01
**CHIP**	WHR (standardized to 0-mean and unit-variance and normalized)	47405	0.031	0.015	3.39E-02
**Large CHIP (VAF>10%)**		47405	0.021	0.022	3.55E-01
TET2		47405	0.074	0.034	3.12E-02
DNMT3A		47405	-0.002	0.018	9.32E-01
*Linear model with adjusting for age, age^2, sex, ever smoker status, and PC1-10					

Supplemental Table 2. Higher frequencies of CHIP mutations in patients with high BMI compared to patients with low BMI.

**Bladder cancer (BLCA)**			
	High BMI	Low BMI	p.value
JAK2	3	2	1
ASXL1	6	3	0.500205
TP53	9	0	0.003538
TET2	3	1	0.622018
PPM1D	3	1	0.622018
**carcinoma/endocervical adenocarcinoma (CESC)**			
	High BMI	Low BMI	p.value
ASXL1	5	1	0.212337
TET2	3	2	1
DNMT3A	2	1	1
**Colon adenocarcinoma (COAD)**			
	High BMI	Low BMI	p.value
TET2	4	2	0.682994
TP53	9	0	0.003503
ASXL1	6	0	0.029399
**Rectum adenocarcinoma (READ)**			
	High BMI	Low BMI	p.value
TP53	5	3	0.70656
ASXL1	4	0	0.11488
TET2	1	0	1
PPM1D	1	0	1
**Skin cutaneous melanoma (SKCM)**			
	High BMI	Low BMI	p.value
TET2	4	0	0.11985
DNMT3A	5	0	0.0586
ASXL1	2	0	0.496127
JAK2	2	1	1
TP53	1	0	1
PPM1D	2	1	1
**Uterine corpus endometrial carcinoma (UCEC)**			
	High BMI	Low BMI	p.value
TP53	19	2	0.000195
TET2	18	5	0.00955
JAK2	15	5	0.038418
PPM1D	5	1	0.216167
ASXL1	13	5	0.091405
DNMT3A	9	2	0.062971

Supplemental Table 3: List of reagents and antibodies

REAGENT or RESOURCE	SOURCE	IDENTIFIER
**Antibodies for Flow cytometry**		
Gr-1, APC/Cy7 (clone: RB6-8C5)	BioLegend	catalog #: 108424
CD11b, PE (clone: M1/70)	BioLegend	catalog #: 101208
CD3, PE/Cy7 (clone: 17A2)	BioLegend	catalog #: 100220
B220, PerCp/Cy5.5 (clone: RA3-6B2)	BioLegend	catalog #: 103236
TER, 119-PE (clone: TER-119)	BioLegend	catalog #: 116208
Gr-1, PE (clone: RB6-8C5)	BioLegend	catalog #: 108408
B220, PE (clone: RA3-6B2)	BioLegend	catalog #: 103208
CD3, PE (clone: 17A2)	BioLegend	catalog #: 100206
c-Kit, APC (clone: ACK2)	BioLegend	catalog #: 135108
Sca-1, PE/Cy7 (clone: D7)	BioLegend	catalog #: 108114
CD48, APC/Cy7 (clone: HM48-1)	BioLegend	catalog #: 103432
CD150, PerCp/Cy5.5 (clone: TC15-12F12.2)	BioLegend	catalog #: 115922
c-Kit, APC/Cy7 (clone:2B8)	BioLegend	catalog #: 105826
CD16/32, PE/Cy7 (clone: 93)	BioLegend	catalog #: 101318
CD34, BV421 (clone: MEC14.7)	BioLegend	catalog #: 119321
CD127, APC (clone: A7R34)	BioLegend	catalog #: 135012
CD45.1, APC (clone: A20)	BioLegend	catalog #: 110714
CD45.2, FITC (clone: 104)	BioLegend	catalog #: 109806
c-Kit, BV785 (clone: ACK2)	BioLegend	catalog #: 135138
CD16/32, APC/Cy7 (clone: 93)	BioLegend	catalog #: 101328
CD3, FITC (clone: 17A2)	BioLegend	catalog #: 100204
CD4, APC (clone: RM4-4)	BioLegend	catalog #: 116014
Calbryte™ 520 AM, FITC	AAT Bioquest	catalog #: 36310
**Antibodies for Western blot/Confocal microscopy**		
Nfatc3 primary antibody conjugated to Alexa Fluor® 488	Santa Cruz Biotechnology	catalog #: sc-8405 AF488
Anti-Nfatc3 antibody	Santa Cruz Biotechnology	catalog #: sc-8405
Anti-Il-1β antibody	Cell Signaling Technologies	catalog #: 12242S
Anti-Irg1 Antibody	Cell Signaling Technologies	catalog #: 17805S
Gapdh	Proteintech	catalog #: 10494-1-AP
**Experimental Models: Organisms/Strains**		
Mouse: *Tet2*$^{-/-}$ and *Tet2*$^{+/-}$	IUSM	N/A
Mouse: *Lep*$^{Ob/Ob}$ (*Ob/Ob*)	Jackson Laboratory	Cat #000632
Mouse: *Tet2*$^{-/-}$;*Ob/Ob*	IUSM	N/A
Mouse: *Dnmt3a*$^{+/-}$;*Mx-Cre*$^{+}$	IUSM	N/A
Mouse: *Dnmt3a*$^{+/-}$;*Mx-Cre*$^{+}$;*Ob/Ob*	IUSM	N/A
Mouse: *Asxl1*$^{+/-}$;*Mx-Cre*$^{+}$	IUSM	N/A
Mouse: *Jak2*$^{V617F/+}$;*Vav-Cre*$^{+}$	IUSM	N/A
Mouse: C57/B6	IUSM	N/A
Mouse: Boy/J	IUSM	N/A
**Critical Commercial Assays**		
Multiplex cytokine assays	Eve Technologies	#MD31
Rneasy Plus Micro Kit (50)	QIAGEN	#74034

SuperScript™ VILO™ cDNA Synthesis Kit	ThermoFisher Scientific	#11754050
TaqMan™ Fast Advanced Master Mix	Applied Biosystems	#4444557
Quest 5-hmC™ DNA ELISA Kit	ZYMO research	#D5425
Accu-Chek glucometer	Roche Diagnostics	#07453736001
**Oligonucleotides**		
Irg1	Thermofisher	#Mm01224531_m1
Gapdh	Thermofisher	#Mm99999915_g1
Nfatc3	Thermofisher	#Mm01249200_m1
**Chemicals**		
Metformin	Selleckchem	catalog #: S1950
Pioglitazone	MedChemExpress	catalog #: HY-13956
Nifedipine	Abcam	catalog #: Ab120135
MCC950	Selleckchem	catalog #: S7809
Anakinra (IL1R antagonist)	MedChemExpress	catalog #: HY-108841
SKF-96365	MedChemExpress	catalog #: HY-100001
UltraCruz® blocking reagent	Santa Cruz Biotechnology	catalog #: sc-516214
**Software and Algorithms**		
FlowJo	FlowJo	v10.7.0
Prism	GraphPad Software	v7.0
Differential gene expression	DESeq	v2.0.
GSEA	clusterProfiler	v4.0
CluGo	Cytoscape	V3.9.1
Molecular Signatures Database	MSigDB	v7.5
**Deposited data**		
Mouse RNA-seq data	NCBI-GEO	GSE193062
**Human data source**		
Study population data	UK Biobank	https://www.ukbiobank.ac.uk/
UK Biobank Resource	Application #7089	N/A
CHIP detection	GATK Mutect2 software	https://software.broadinstitute.org/gatk
WHR (Waist-to-hip ratio)	Seca 200	N/A
Body weight measurement	Tanita BC-418MA bio-impedance analyzer	N/A

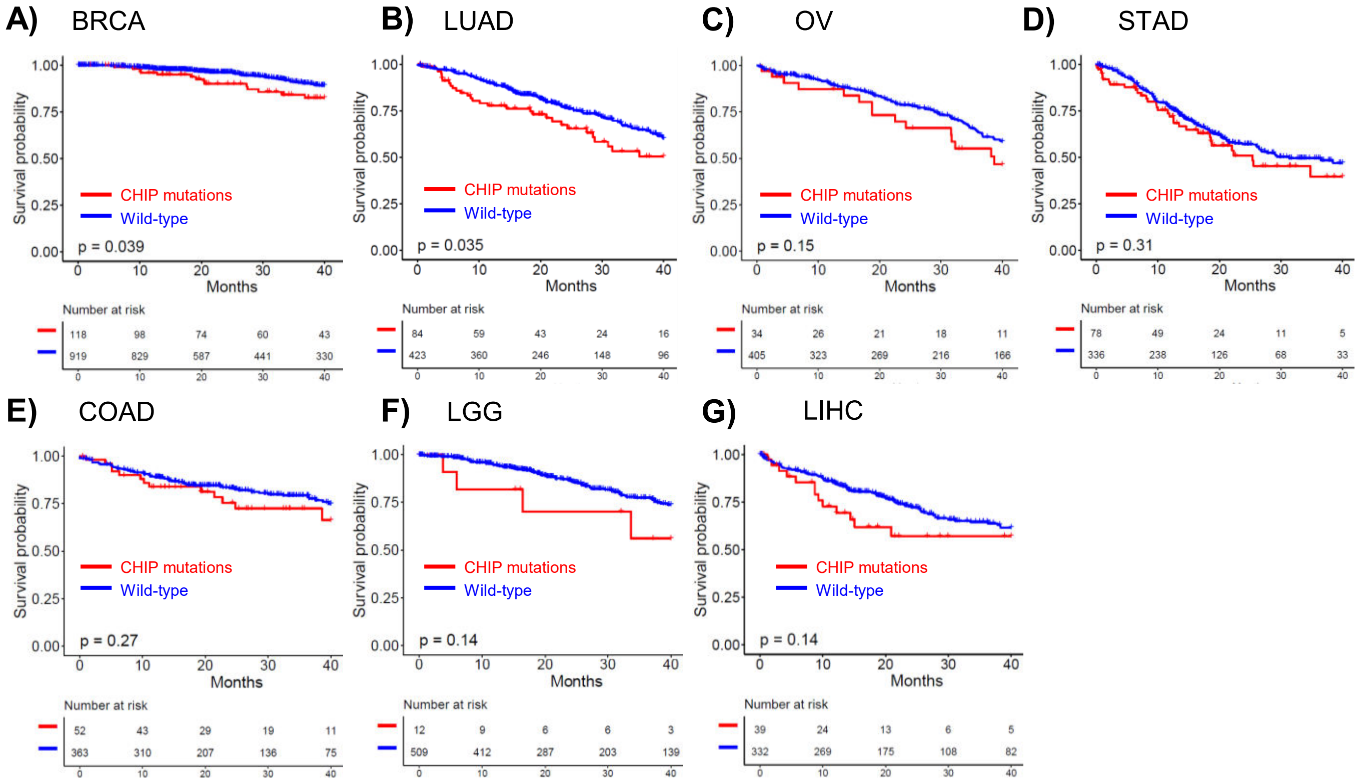

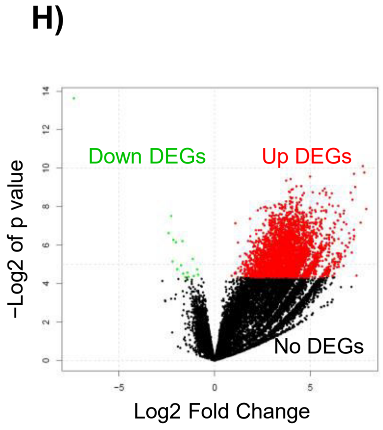

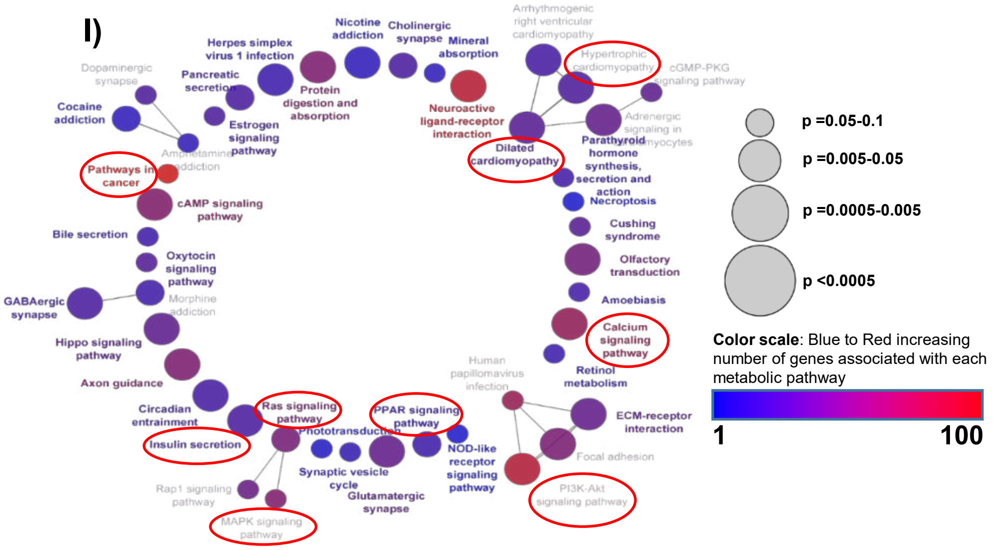

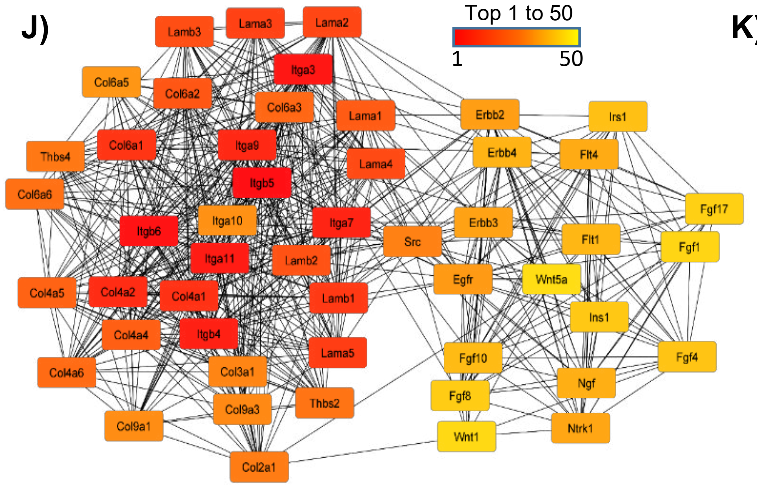

K)

Pathway	Hits	Pathway size	p.value
1. Glycine serine and threonine metabolism	9	31	0.042683
2. Taurine and hypotaurine metabolism	5	10	0.010441
3. PPAR signaling pathway	18	67	0.015324
4. Calcium signaling pathway	49	173	9.46E-06
5. Neuroactive ligand receptor interaction	73	263	1.47E-07

Supplemental Figure 1

Supplemental Figure 1: Patients with CHIP mutations show poor overall survival compared to patients without CHIP mutations in non-hematologic malignancies

(A-G) Overall survival of patients with CHIP mutations versus patients without CHIP mutations in 7 different cancer types including, breast (BRCA) (n=1037; p=0.039), lung (LUAD) (n=507; p=0.035), ovarian (OV) (n=439; p=0.15), stomach (STAD) (n=414; p=0.31), colon (COAD) (n=415; p=0.27), low grade glioma (LGG) (n=521; p=0.14), and liver (LIHC) (n=371; p=0.14) cancer.

(H) Volcano plot showing fold change and FDR adjusted p value in *Ob/Ob* mice bearing *Tet2*$^{-/-}$ cells compared to WT recipients.

(I) ClueGo analysis using the Cytoscape platform confirmed that some of the 433 differentially expressed genes (DEGs) in *Ob/Ob* mice bearing *Tet2*$^{-/-}$ cells compared to WT recipient mice. Color scale: Blue to Red increasing number of genes associated with each metabolic pathway. All pathways represented are with p value <0.05, size of the node explains the significant p values.

(J) A global protein-protein interaction network map was derived from the 433 DEGs followed by clustering analysis. The clustering analysis revealed the essential hot spots/hubs of the protein network. The top 50 up-regulated hot spot genes were extracted from the network and their significance with relevant pathways was interpreted.

(K) IPA analysis of differentially expressed genes in *Ob/Ob* mice bearing *Tet2*$^{-/-}$ cells compared to WT recipient mice.

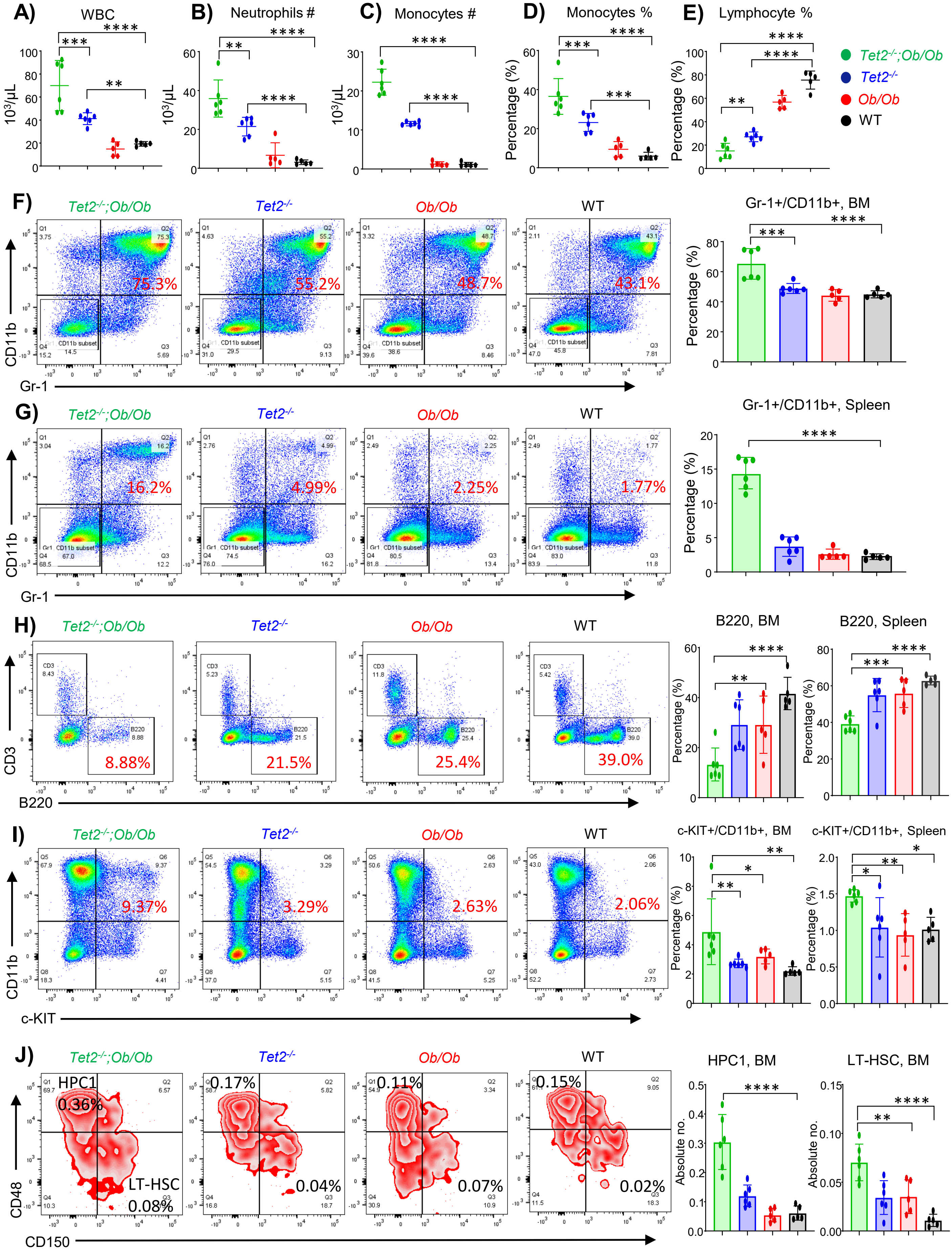

Supplemental Figure 2

Supplemental Figure 2: Obesity induces expansion of pre-leukemic (*Tet2*$^{-/-}$) HSC/Ps

(A-E) Hematologic analysis of PB using the automated blood cell counter (Element HT5 Hematology analyzer, HESKA) in indicated genotypes.

(F) Representative flow cytometry profile of myeloid cells (Gr-1^{+}/CD11b^{+}) in the indicated genotypes, and frequency of Gr-1^{+}/CD11b^{+} cells in the BM from the indicated genotypes.

(G) Representative flow cytometry profile of myeloid cells (Gr-1^{+}/CD11b^{+}) in the indicated genotypes, and frequency of Gr-1^{+}/CD11b^{+} cells in spleens from the indicated genotypes.

(H) Representative flow cytometry profile of T cells and B cells (CD3^{+}/B220^{+} cells) in the indicated genotypes, and frequency of B220^{+} cells in the BM and spleens from the indicated genotypes.

(I) Representative flow cytometry profile of myeloid blasts (c-KIT^{+}/CD11b^{+} cells) in the indicated genotypes, and frequency of c-KIT^{+}/CD11b^{+} cells in the BM and spleens from the indicated genotypes.

(J) Representative flow cytometry profile of HPC-1 cells (LSK/CD48^{+}/CD150^{-}) and LT-HSCs (LSK/CD48^{-}/CD150^{+}) compartment and quantification of absolute number of total HPC-1 cells and LT-HSCs in the BM from the indicated genotypes. (n= 5 to 6 mice per group). Data are shown as the mean ± SEM. *$p<0.05$, **$p<0.005$, ***$p<0.0005$, ****$p<0.0001$, by one-way ANOVA.

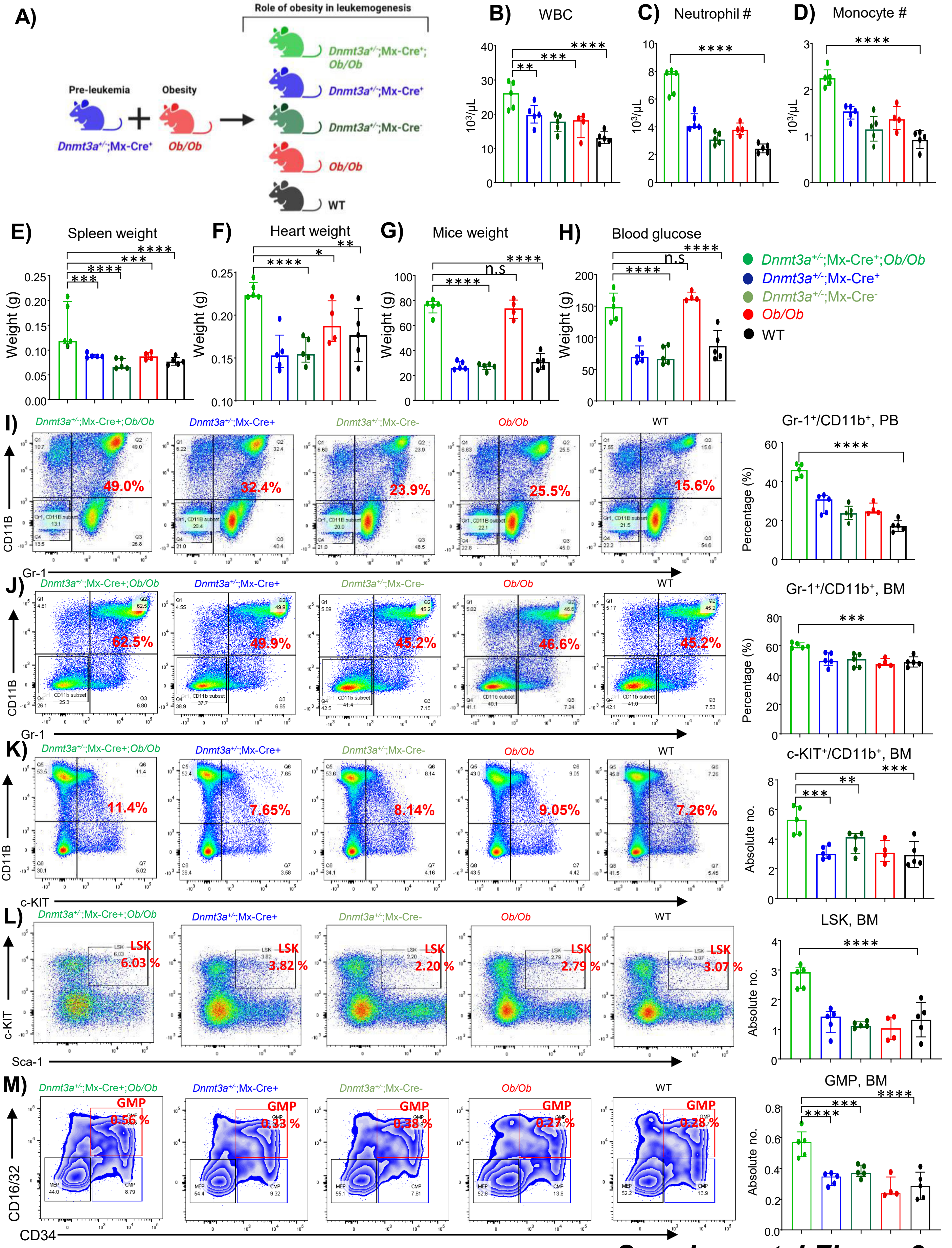

Supplemental Figure 3

Supplemental Figure 3: Haploinsufficiency of *Dnmt3a* causes clonal expansion and myeloid malignancy under obese conditions

(A) Schematic for generating *Dnmt3a*$^{+/-}$;Mx-Cre^{+};*Ob/Ob* compound mutant mice along with *Dnmt3a*$^{+/-}$;Mx-Cre^{+}, *Dnmt3a*$^{+/-}$;Mx-Cre^{-}, *Ob/Ob* and WT control mice.

(B-D) Examination of PB WBCs, neutrophils and monocytes in indicated genotypes.

(E-H) (E) Spleen weights **(F)** heart weights and **(G)** mice weight and **(H)** fasting blood glucose levels of indicated genotypes.

(I) Representative flow cytometry profile of myeloid cells (Gr-1^{+}/CD11b^{+} double positive cells) in the PB and frequency of myeloid cells in the PB of indicated genotypes.

(J) Representative flow cytometry profile of myeloid cells in the BM and frequency of myeloid cells in the BM of indicated genotypes.

(K) Representative flow cytometry profiles of myeloid blasts (c-KIT^{+}/CD11b^{+} cells) in the BM and absolute number of myeloid blasts in the BM.

(L) Representative flow cytometry profiles of LSK cells in the BM of indicated genotypes and absolute number of LSK cells in the BM.

(M) Representative flow cytometry profiles of GMPs in the BM of indicated genotypes and absolute number of total GMPs in the BM. (n= 4 to 5 mice per group). Data are shown as the mean ± SEM. *p<0.05, **p<0.005, ***p<0.0005, ****p<0.0001, by one-way ANOVA.

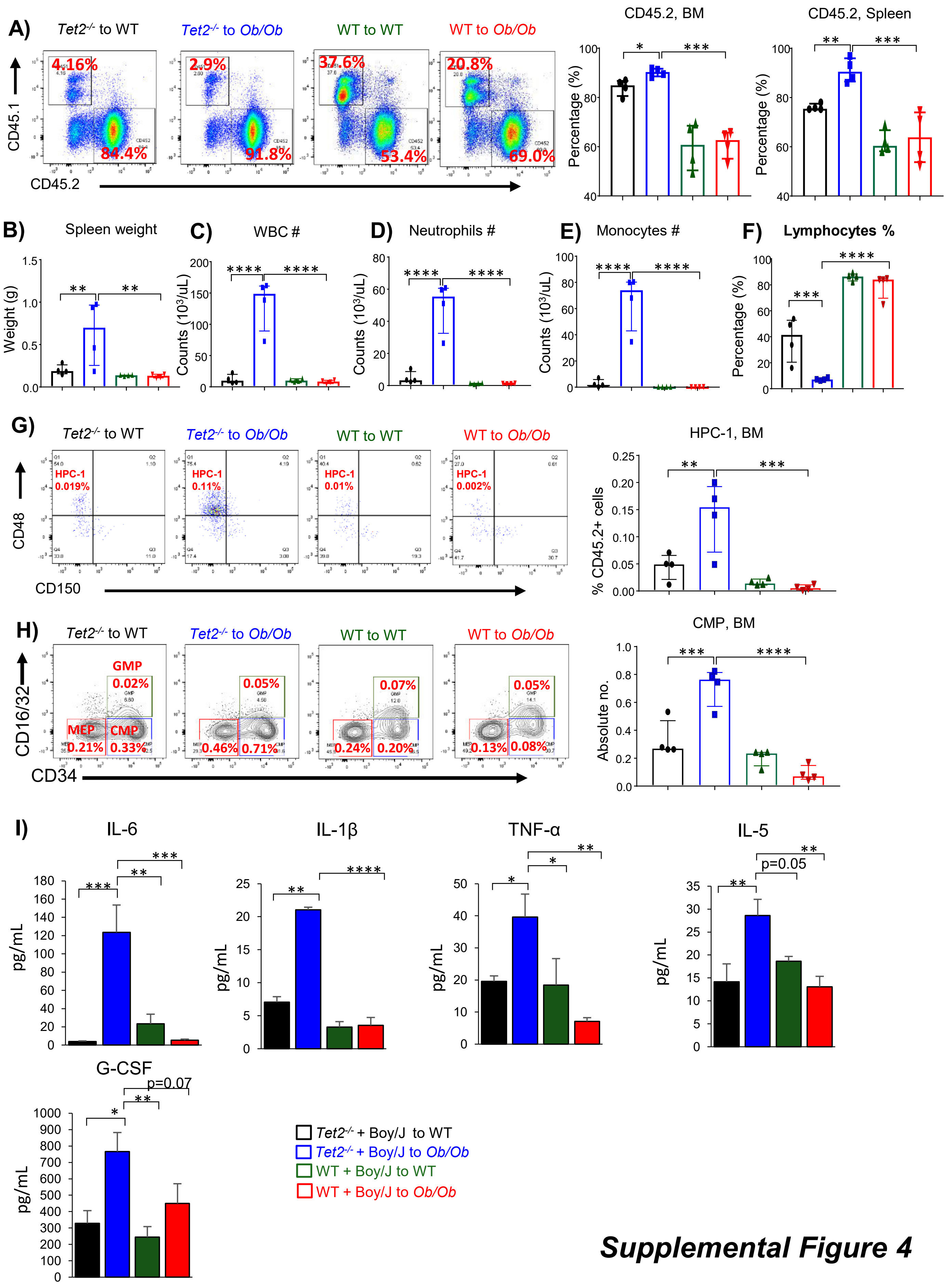

Supplemental Figure 4

Supplemental Figure 4: Development of MPN in obese recipients is due to the expansion of *Tet2*$^{-/-}$ HSC/Ps in the BM and pro-inflammatory cytokines in the serum

(A) Representative flow cytometry plots of donor chimerism ($CD45.1^{+}/CD45.2^{+}$) in the BM of indicated recipient mice and frequency of $CD45.2^{+}$ cells in the BM and spleens of recipient mice over 22 weeks.

(B-F) Spleen weights and PB counts at 22 weeks after BMT from the indicated recipient mice.

(G) Representative flow cytometry profile of CD48/CD150 cells in the BM and frequency of HPC-1 cells in the BM of competitive transplant recipients over 22 weeks.

(H) Representative flow cytometry profile of common myeloid progenitors (CMPs; Lin^{-}/c-$KIT^{+}/CD34^{+}/CD16/32^{-}$) and absolute number of total CMPs in the BM of competitive transplant recipients over 22 weeks.

(I) Elevated serum cytokines, including IL-6, IL-1β, TNF-α, IL-5, and G-CSF were observed in *Ob/Ob* mice bearing *Tet2*$^{-/-}$ cells compared to controls (at 22 weeks of post-transplantation). (n= 4 mice per group). Data are shown as the mean ± SEM. *$p<0.05$, **$p<0.005$, ***$p<0.0005$, ****$p<0.0001$, by Student's *t*-test (2-tailed) (**A** and **I**) or one-way ANOVA (**B-H**).

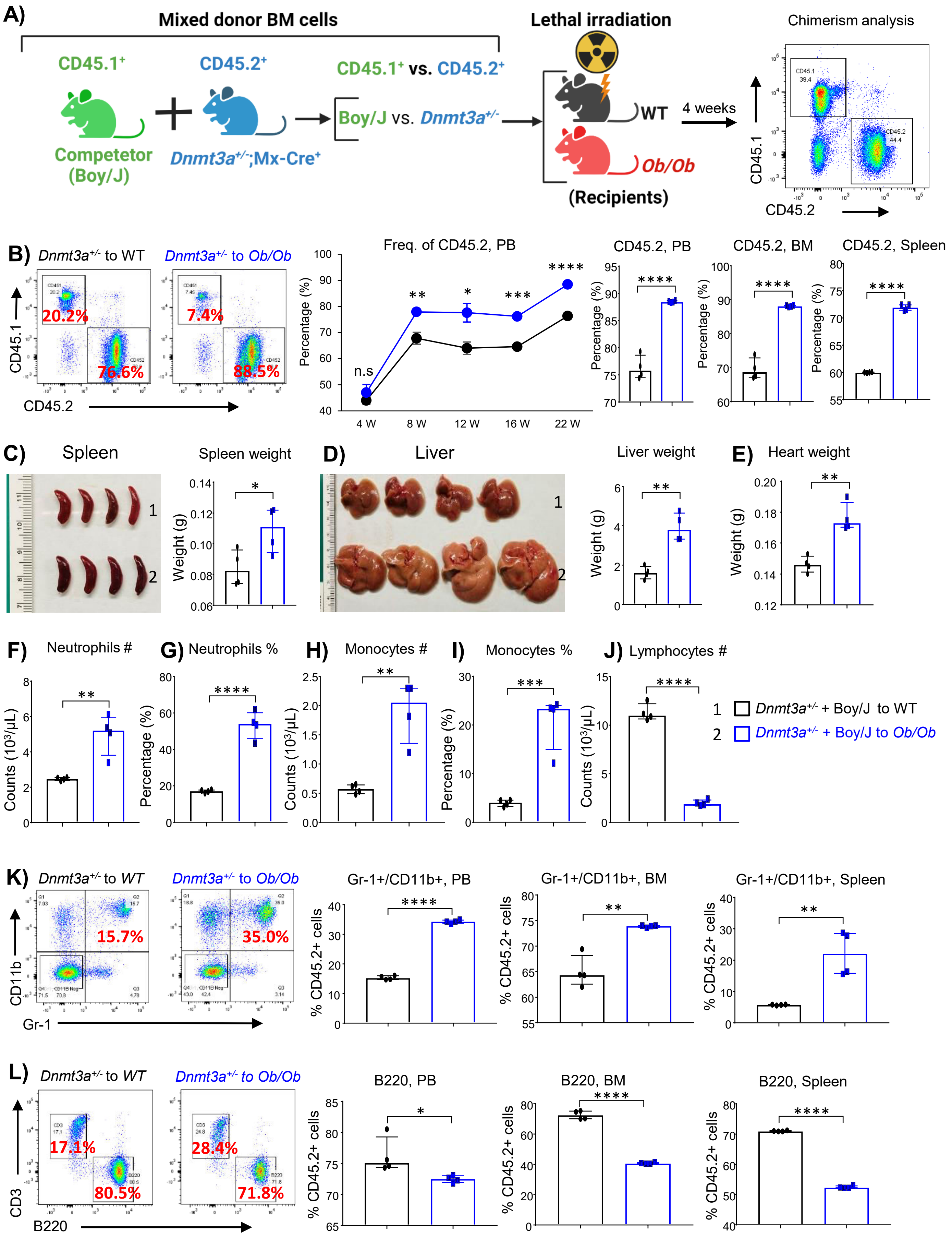

Supplemental Figure 5

Supplemental Figure 5: *Dnmt3a*$^{+/-}$ mutant myeloid cells rapidly expanded in obese FBM recipients

(A) Schematic of the competitive bone marrow transplantation (BMT) assay. Donor cells from pIpC treated *Dnmt3a*$^{+/-}$;Mx-Cre^{+} mice were mixed with Boy/J cells and a competitive BMT assay was performed using lethally irradiated WT or *Ob/Ob* mice as recipients. Donor-derived chimerism was observed using antibodies against CD45.1^{+} or CD45.2^{+}.

(B) Representative flow cytometry plots of donor chimerism (CD45.1^{+}/CD45.2^{+}), PB chimerism was measured at every 4 weeks after BMT and quantification of CD45.2^{+} cells in the PB, BM and spleens of recipient mice at 22 weeks after BMT.

(C-E) **(C)** Spleen weight, **(D)** liver weight and **(E)** heart wight examined at 22 weeks after BMT.

(F-J) Examination of PB counts at 22 weeks after BMT from the indicated recipient mice.

(K) Representative flow cytometry profile of myeloid cells (Gr-1^{+}/CD11b^{+}) and quantification of Gr-1^{+}/CD11b^{+} in the PB, BM and spleens of recipient mice at 22 weeks after BMT.

(L) Representative flow cytometry profile of CD3^{+}/B220^{+} cells and quantification of B220^{+} in the PB, BM and spleens of recipient mice at 22 weeks after BMT. (n= 4 mice per group). Data are shown as the mean ± SEM. *p<0.05, **p<0.005, ***p<0.0005, ****p<0.0001, by Student's *t*-test (2-tailed).

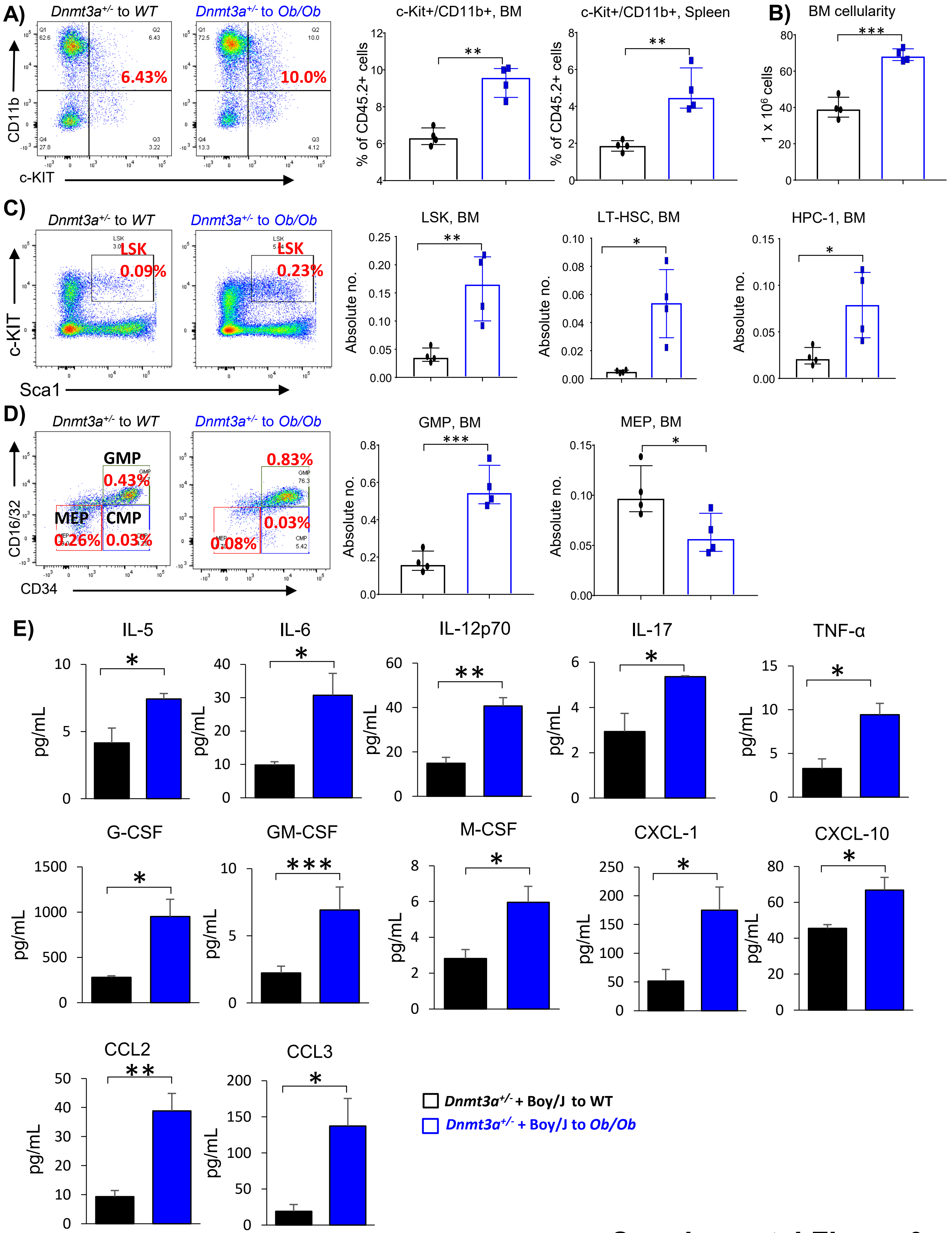

Supplemental Figure 6

Supplemental Figure 6: Obesity drives *Dnmt3a*$^{+/-}$ mutant HSC/Ps expansion and up-regulation of pro-inflammatory cytokines

(A) Representative flow cytometry profile of c-KIT^{+}/CD11b^{+} double positive cells and frequency of myeloid blasts (c-KIT^{+}/CD11b^{+} cells) in the BM and spleens of recipient mice at 22 weeks after BMT.

(B, C) (**B**) BM cellularity, and **(C)** representative flow cytometry profile of LSK cells in the BM and absolute number of total LSK cells, LT-HSCs and HPC-1 cells in the BM of recipient mice at 22 weeks after BMT.

(D) Representative flow cytometry profile of progenitors in the BM and frequency of total GMPs and megakaryocyte erythroid progenitors (MEPs; Lin−/c-Kit^{+}/CD34^{-}/CD16/32^{-} cells) in the BM of recipient mice at 22 weeks after BMT.

(E) Analysis of serum cytokine and chemokines, *IL-5*, *IL-6*, *IL-12p70*, *IL-17*, *TNF-α*, *G-CSF*, *GM-SCF*, *M-CSF*, *CXCL-1*, *CXCL-10*, *CCL-2*, and *CCL-3* concentrations in the serum of recipient mice at 22 weeks after BMT. (n= 4 mice per group). Data are shown as the mean ± SEM. *p<0.05, **p<0.005, ***p<0.0005, ****p<0.0001, by Student's *t*-test (2-tailed).

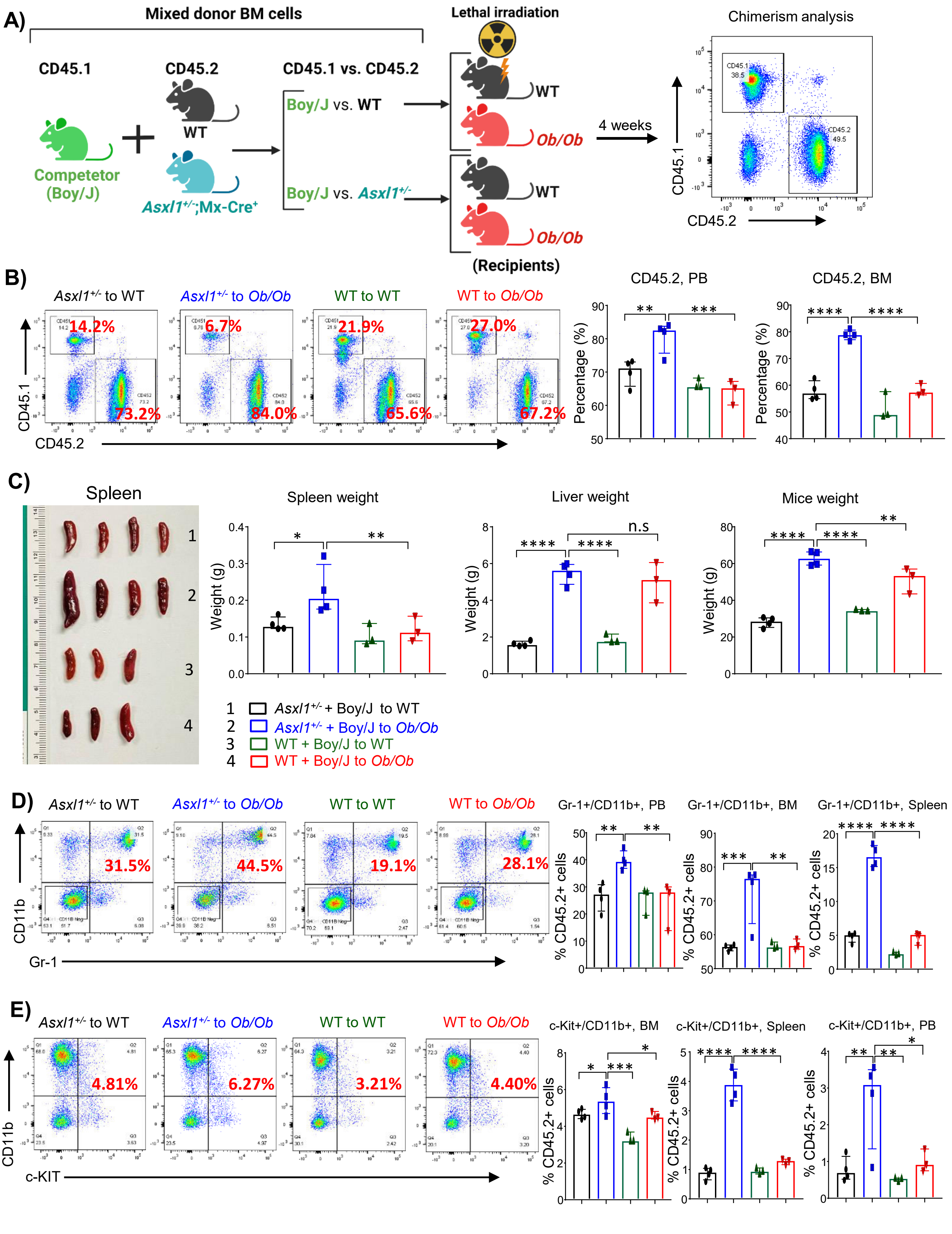

Supplemental Figure 7

Supplemental Figure 7: *Asxl1$^{+/-}$* mutant myeloid cells rapidly expanded in obese recipients

(A) Schematic of the competitive BMT assay. Donor BM cells from pIpC treated *Asxl1$^{+/-}$*;Mx-Cre^{+} or WT mice were mixed with Boy/J BM cells and a competitive BMT assay was performed using lethally irradiated WT or *Ob/Ob* mice as recipients. Donor-derived chimerism was observed using antibodies against CD45.1^{+} or CD45.2^{+}.

(B) Representative flow cytometry profiles of donor chimerism (CD45.1^{+}/CD45.2^{+}) in the PB and frequency of CD45.2^{+} in the PB and BM of recipient mice at 26 weeks after BMT.

(C) Spleen pictures and quantification of spleen weights, liver weights and mice weights were examined at 26 weeks after BMT.

(D) Representative flow cytometry profile of myeloid cells (Gr-1^{+}/CD11b^{+} cells) and frequency of Gr-1^{+}/CD11b^{+} cells in the PB, BM and spleens of recipient mice at 26 weeks after BMT.

(E) Representative flow cytometry profile of myeloid blasts (c-KIT^{+}/CD11b^{+} cells) and frequency of c-KIT^{+}/CD11b^{+} cells in the BM, spleens and PB of recipient mice at 26 weeks after BMT. (n= 4 mice per group). Data are shown as the mean ± SEM. *p<0.05, **p<0.005, ***p<0.0005, ****p<0.0001, by one-way ANOVA.

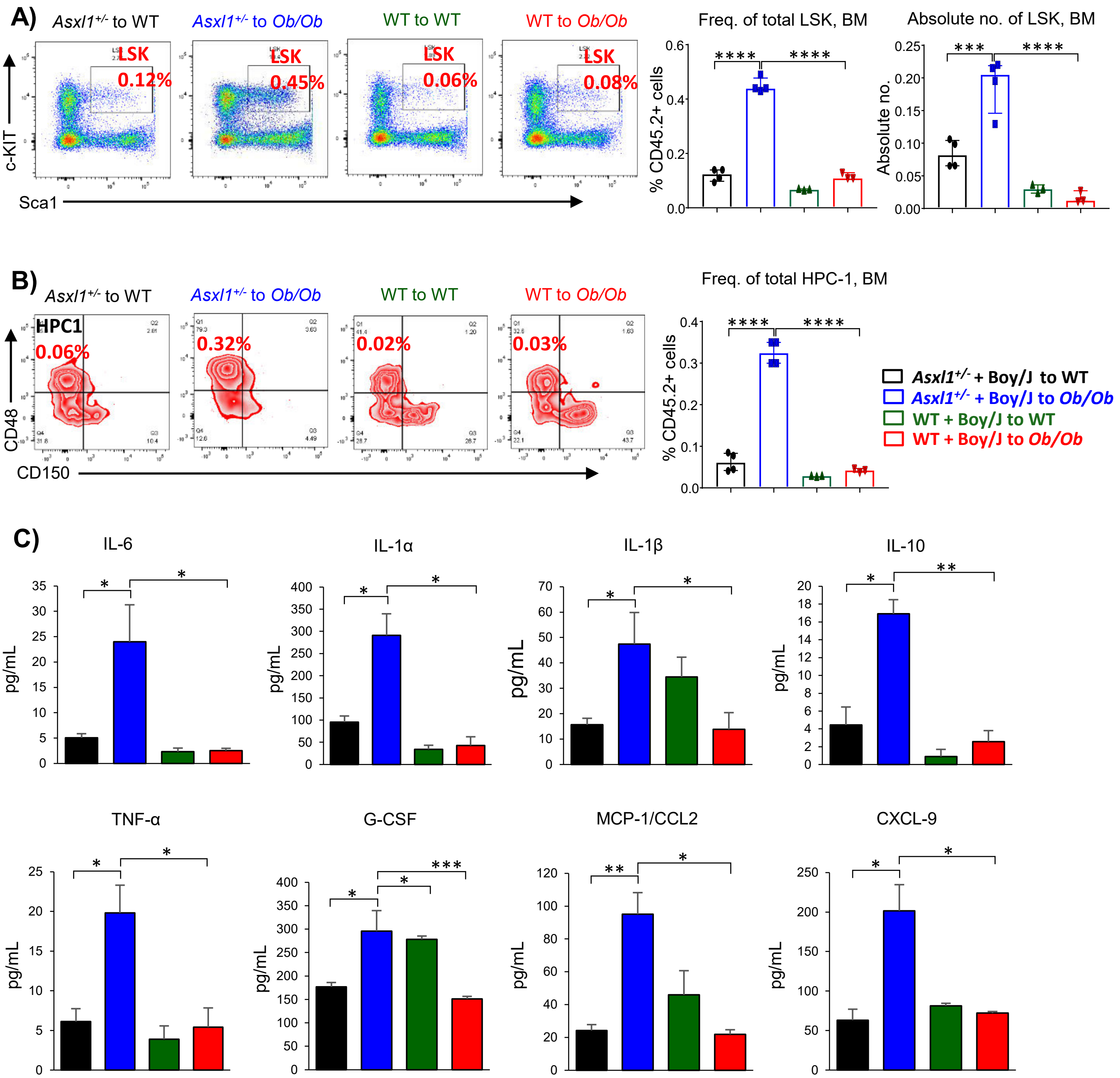

Supplemental Figure 8: Obesity drives *Asxl1*$^{+/-}$ mutant HSC/P expansion and upregulation of pro-inflammatory cytokines

(A) Representative flow cytometry profile of LSK cells in the BM, quantification of frequency and absolute number of total LSK cells in the BM of recipient mice at 26 weeks after BMT.

(B) Representative flow cytometry profile of CD48/CD150 cells and frequency of total HPC-1 cells and LT-HSCs in the BM of recipient mice at 26 weeks after BMT.

(C) Analysis of serum cytokines and chemokines, *IL-6*, *IL-1α*, *IL-1β*, *IL-10*, *TNF-α*, *GM-CSF*, *CCL-2* and *CXCL-9* concentrations in the serum of recipient mice at 26 weeks after BMT. (n= 4 mice per group). Data are shown as the mean ± SEM. *p<0.05, **p<0.005, ***p<0.0005, ****p<0.0001, by one-way ANOVA (**A** and **B**) or Student's *t*-test (2-tailed) (**C**).

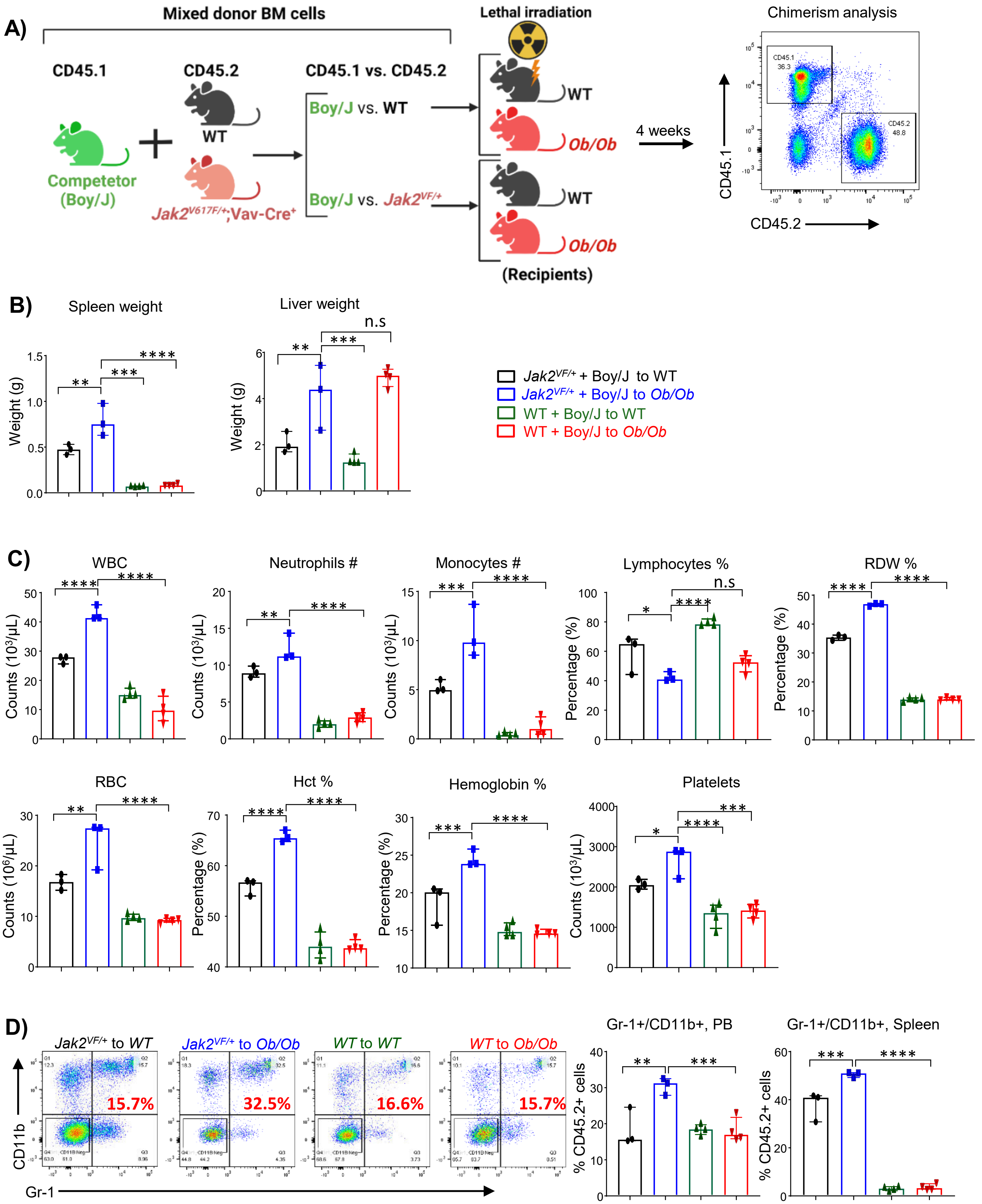

Supplemental Figure 9

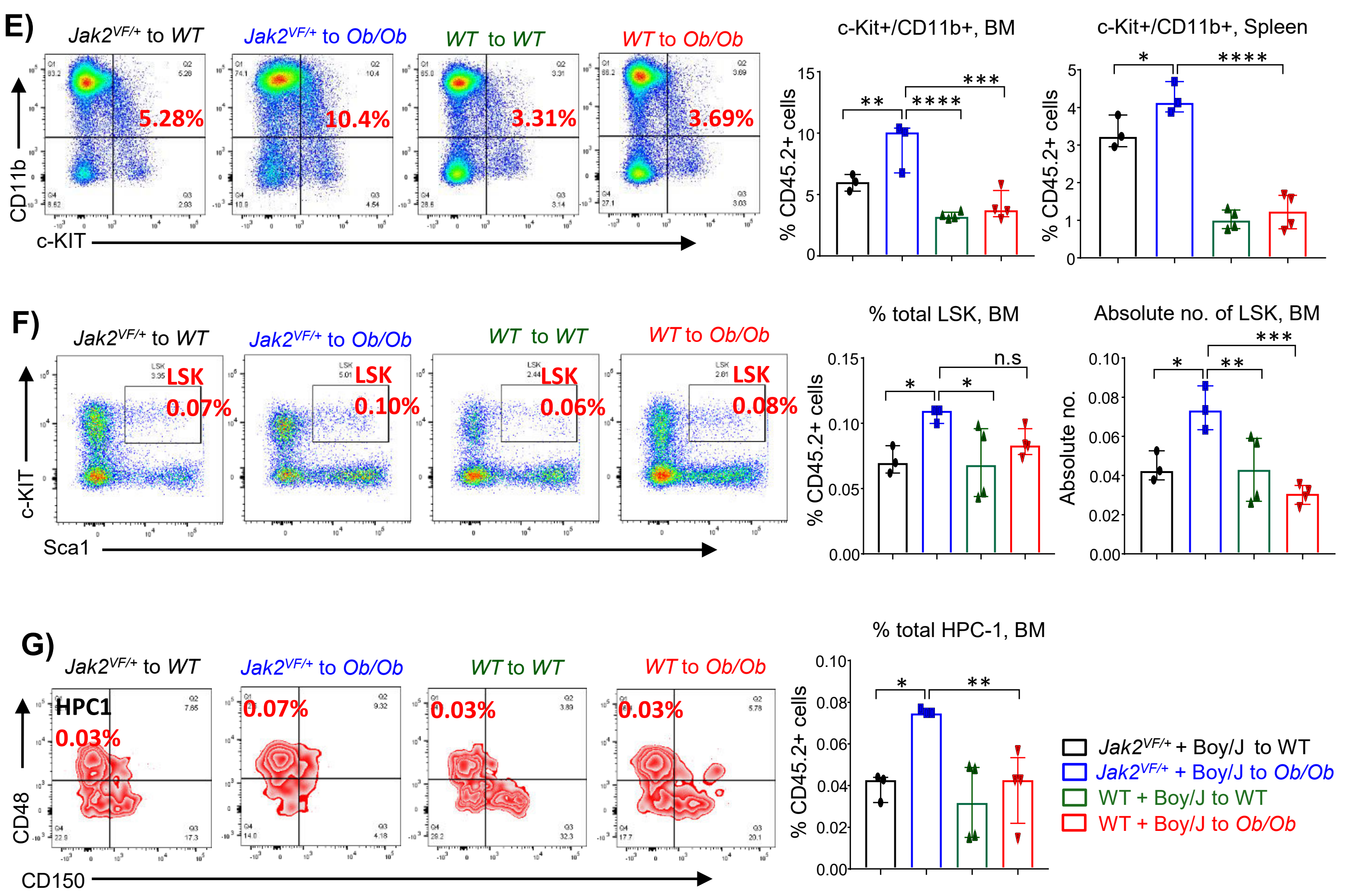

Supplemental Figure 9: *Jak2$^{V617F/+}$* mutant myeloid cells are rapidly expanded in obese recipients

(A) Schematic of the competitive BMT assay. Donor BM cells from *Jak2$^{V617F/+}$*;Vav-Cre^{+} or WT mice were mixed with Boy/J BM cells and a competitive BMT assay was performed using lethally irradiated WT or *Ob/Ob* mice as recipients. Donor-derived chimerism was observed using antibodies against CD45.1^{+} or CD45.2^{+}.

(B) Spleen and liver weights were examined at 28 weeks after BMT.

(C) Elevated PB counts including, WBCs, neutrophils, monocytes, RDW %, RBCs, hematocrit %, hemoglobin levels, platelet counts and decreased lymphocyte % were observed in *Jak2$^{V617F/+}$*;*Ob/Ob* mice compared to other groups.

(D) Representative flow cytometry profile of myeloid cells (Gr-1^{+}/CD11b^{+} double positive cells) and frequency of Gr-1^{+}/CD11b^{+} cells in the PB and spleens of recipient mice at 28 weeks after BMT.

(E) Representative flow cytometry profile of myeloid blasts (c-KIT^{+}/CD11b^{+} double positive cells) in the BM and frequency of c-KIT^{+}/CD11b^{+} cells in the BM and spleens of recipient mice at 28 weeks after BMT.

(F) Representative flow cytometry profile of LSK cells in the BM, quantification of frequency and absolute number of total LSK cells in the BM of recipient mice at 28 weeks after BMT.

(G) Representative flow cytometry profile of HPC-1 cells and LT-HSCs in the BM, frequency of total HPC-1 cells and LT-HSCs in the BM of recipient mice at 28 weeks after BMT. (n= 4 mice per group). Data are shown as the mean ± SEM. *$p<0.05$, **$p<0.005$, ***$p<0.0005$, ****$p<0.0001$, by one-way ANOVA.

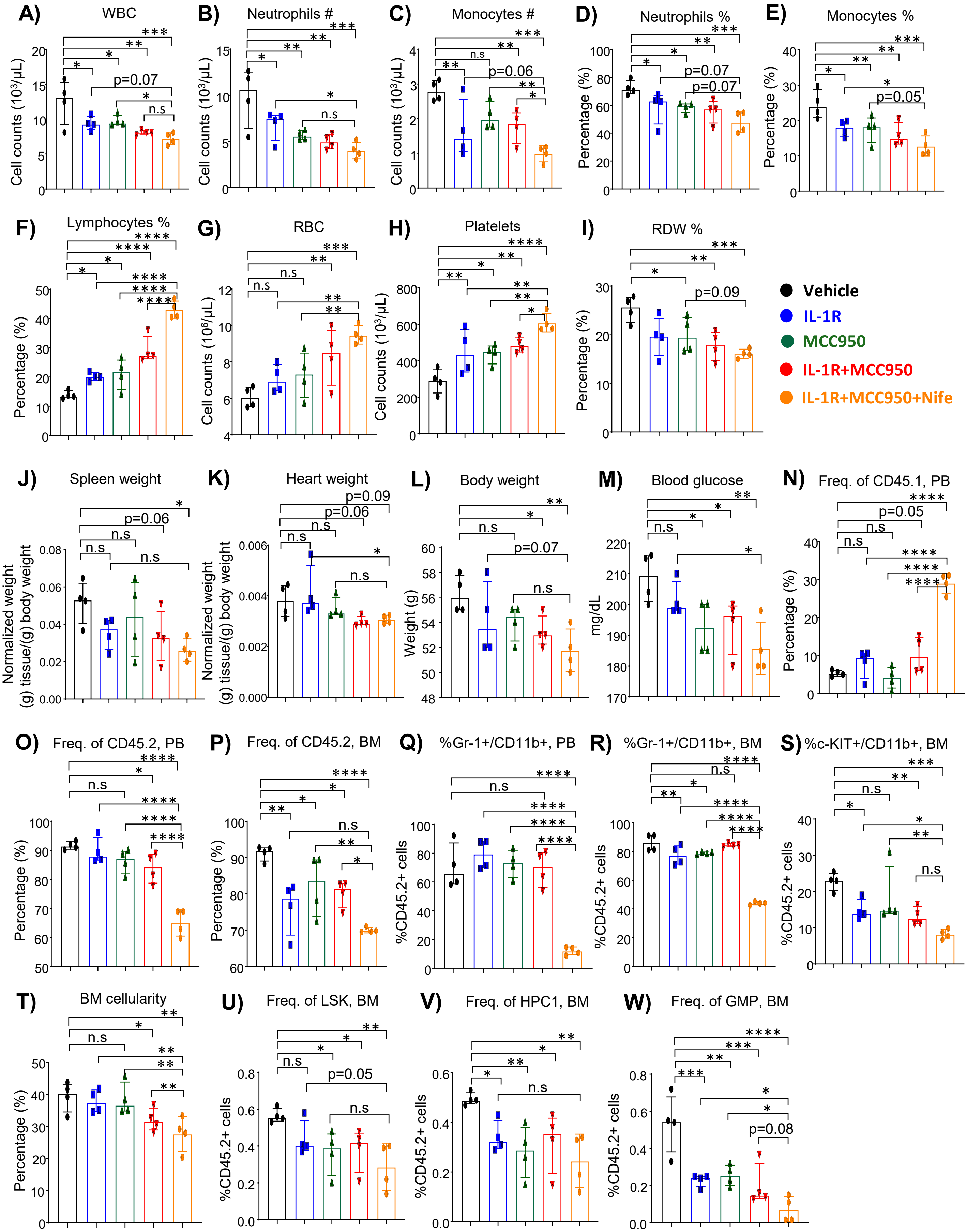

Supplemental Figure 10

Supplemental Figure 10: Effect of MCC950, anakinra and nifedipine treatment on CH

(A-I) PB counts (**A**) WBCs, **(B)** neutrophil counts, **(C)** monocyte counts, **(D)** neutrophil %, **(E)** monocyte %, **(F)** lymphocyte %, **(G)** RBCs, **(H)** platelet counts and **(I)** RDW % after 30 days of indicated drug treatment.

(J-M) Spleen weights, heart weights, body weights, and fasting blood glucose levels after 30 days of the indicated drug treatments.

(N-S) Percentages of CD45.1, CD45.2, myeloid cells (Gr-1^{+}/CD11b^{+}), and myeloid blasts (c-KIT^{+}/CD11b^{+}) in the PB and BM of indicated drug treatments.

(T-W) BM cellularity, percentages of total LSK cells, HPC1 cells and GMPs in the BM of indicated drug treatments. (n= 4 mice per group). Data are shown as the mean ± SEM. *p<0.05, **p<0.005, ***p<0.0005, ****p<0.0001, by one-way ANOVA.

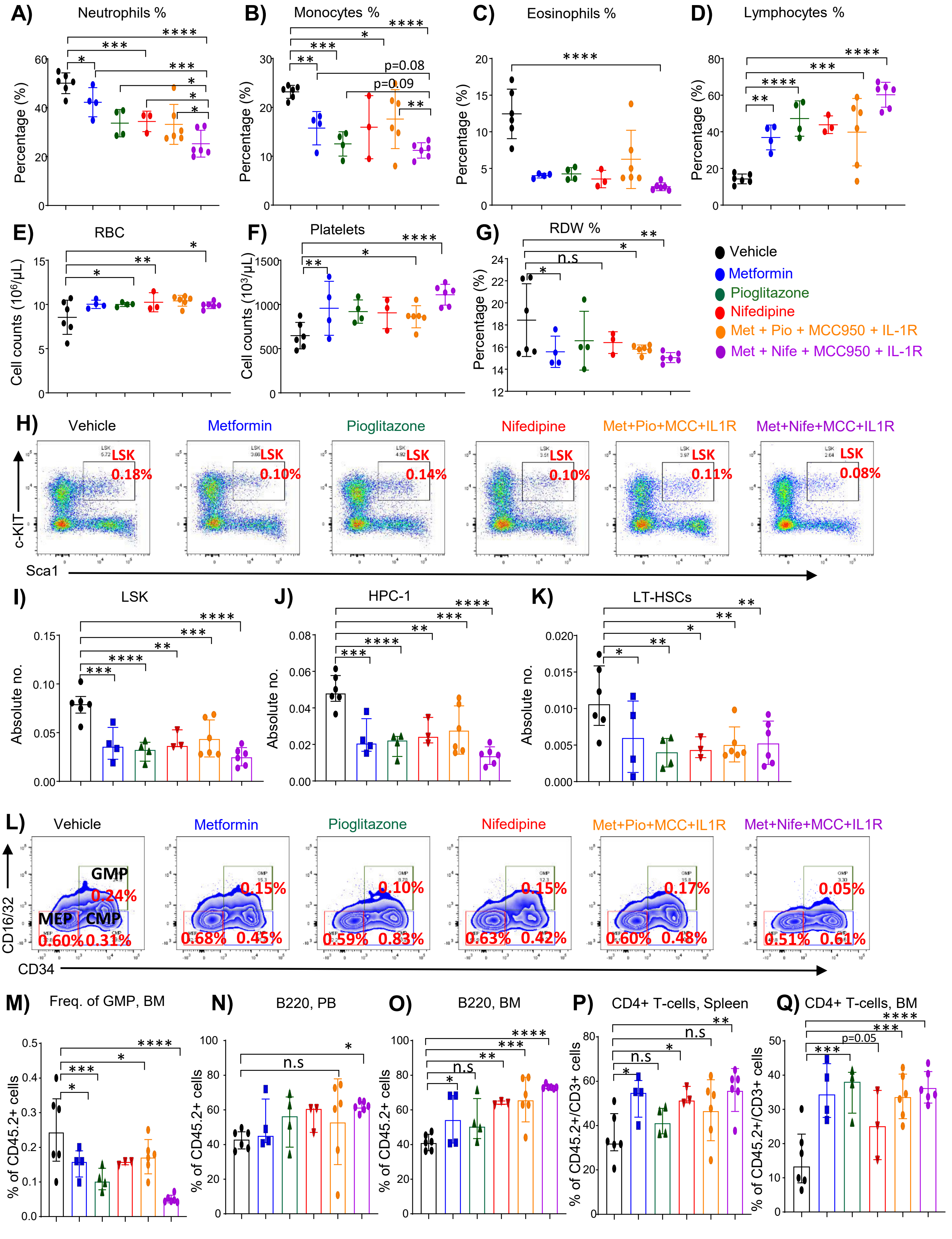

Supplemental Figure 11

Supplemental Figure 11: Combination of metformin, nifedipine, MCC950 and anakinra treatment rescued myeloid skewing and *Tet2*$^{-/-}$ pre-LHSC/Ps

(A-G) PB counts **(A)** neutrophil %, **(B)** monocyte %, **(C)** eosinophil %, **(D)** lymphocyte %, **(E)** RBCs, **(F)** platelet counts and **(G)** RDW % after 30 days of indicated drug treatment.

(H) Representative flow cytometry profile of LSK cells in the BM of recipient mice after 30 days of indicated drug treatment and **(I)** frequency of total LSK cells in the BM after 30 days of the indicated drug treatments.

(J) Absolute number of total HPC-1 cells (LSK/CD48^{+}/CD150^{-}) and **(K)** total LT-HSCs (LSK/CD48^{-}/CD150^{+}) in the BM after 30 days of the indicated drug treatments.

(L) Representative flow cytometry profile of progenitors in the BM of recipient mice after 30 days of indicated drug treatment and **(M)** frequency of total GMPs (Lin-/C-KIT^{+}/CD16/32^{+}/CD34^{+}) in the BM after 30 days of indicated drug treatment.

(N, O) Frequency of B220^{+} B-cells in the PB **(N)** and BM **(O)** after 30 days of the indicated drug treatments.

(P, Q) Frequency of CD4^{+} T-cells in the spleen **(P)** and BM **(Q)** after 30 days of the indicated drug treatments. (n= 3 to 6 mice per group). Data are shown as the mean ± SEM. *p<0.05, **p<0.005, ***p<0.0005, ****p<0.0001, by one-way ANOVA.

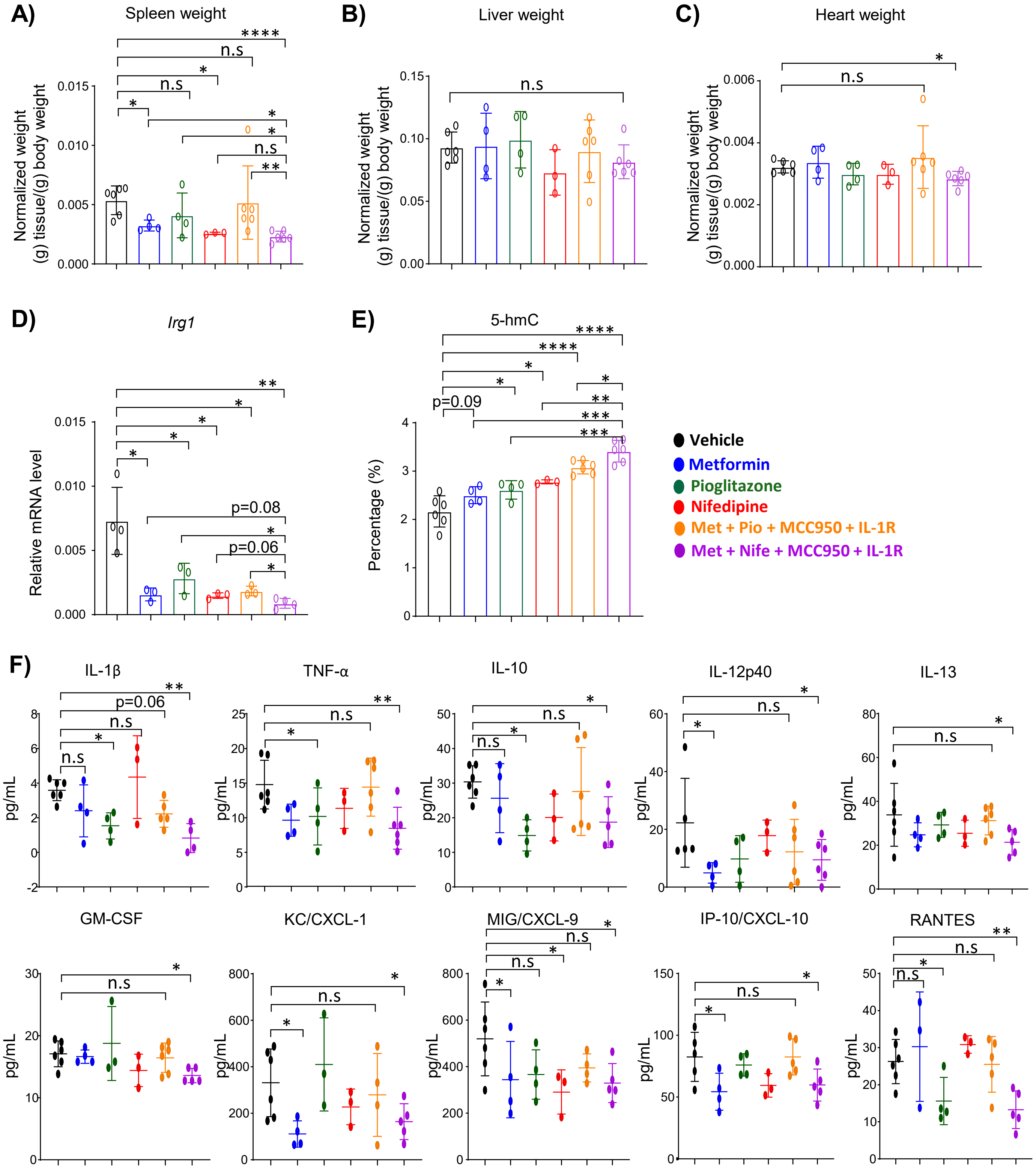

Supplemental Figure 12: Combination of metformin, nifedipine, MCC950 and anakinra treatment suppress the inflammatory cytokines

(A-C) Normalized spleen, liver, and heart weights of *Ob/Ob* recipient mice after 30 days of the indicated drug treatments.

(D, E) *Irg1* and 5-hmC levels in *Ob/Ob* recipient mice after 30 days of the indicated drug treatments.

(F) Serum cytokine and chemokine analysis including, *IL-1β*, *TNF-α*, *IL-10*, *IL-12p40*, *IL-13*, *GM-CSF*, *KC/CXCL-1*, *MIG/CXCL-9*, *IP-10/CXCL-10* and *RANTES* concentrations in the serum of *Ob/Ob* recipient mice after 30 days of the indicated drug treatment. (n= 3 to 6 mice per group). Data are shown as the mean ± SEM. *p<0.05, **p<0.005, ***p<0.0005, ****p<0.0001, by Student's *t*-test (2-tailed) (**A-E**) or one-way ANOVA (**F**).

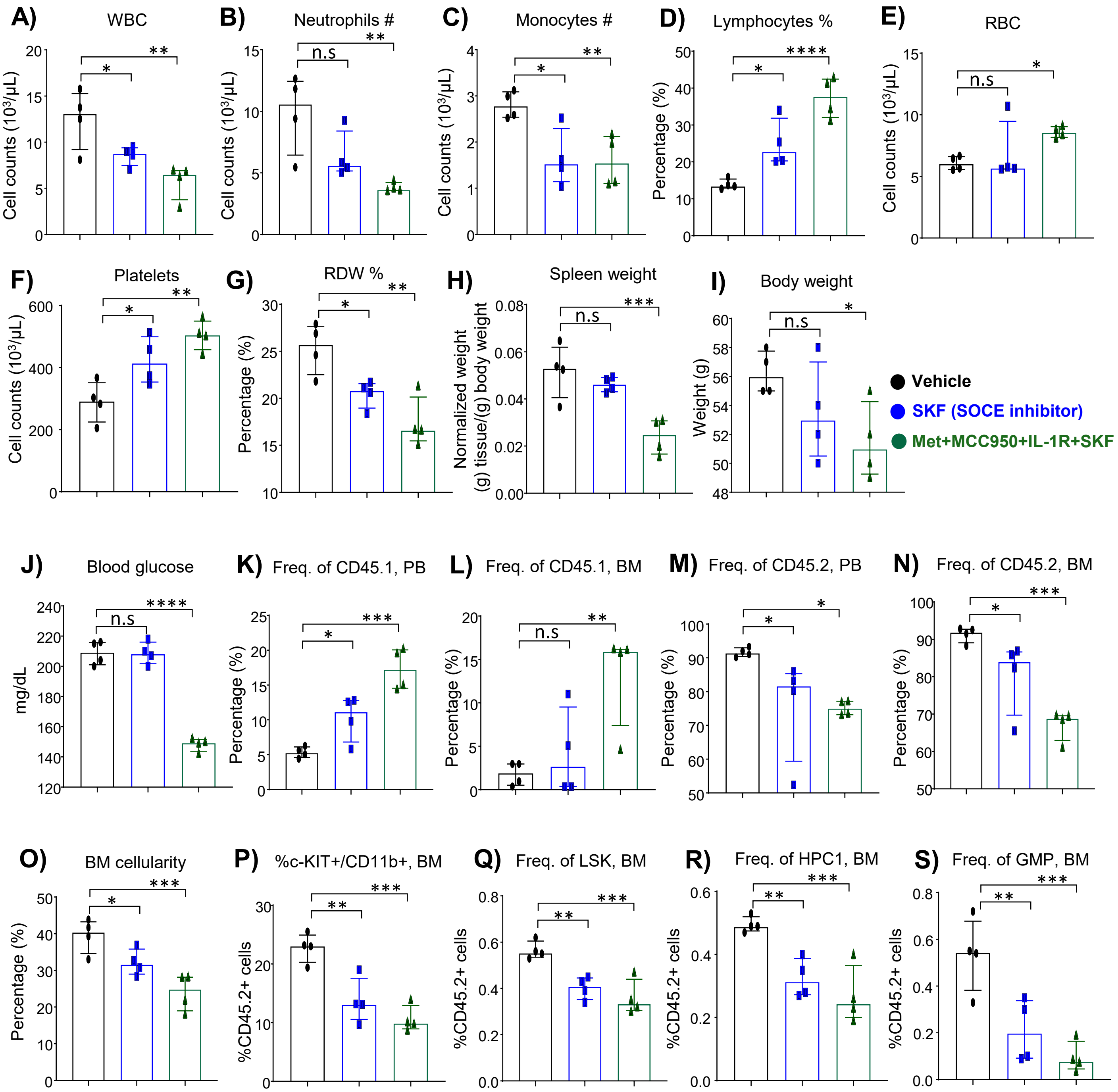

Supplemental Figure 13: Effect of SKF-96365 (store operated calcium channel blocker (SOCE)) on CH
(A-G) PB counts **A)** WBCs, **(B)** neutrophil counts, **(C)** monocyte counts, **(D)** lymphocyte %, **(E)** RBCs, **(F)** platelet counts and **(G)** RDW % after 30 days of indicated drug treatment.
(H-J) Normalized spleen weights, body weights and fasting blood glucose levels after 30 days of the indicated drug treatment.
(K-N) Percentages of CD45.1 and CD45.2 cells in the PB and BM of indicated drug treatments.
(O-S) BM cellularity, percentages of myeloid blasts (c-KIT+/CD11b+), LSK cells, HPC1 cells and GMPs in the BM of indicated drug treatments. (n= 4 mice per group). Data are shown as the mean ± SEM. *p<0.05, **p<0.005, ***p<0.0005, ****p<0.0001, by one-way ANOVA.

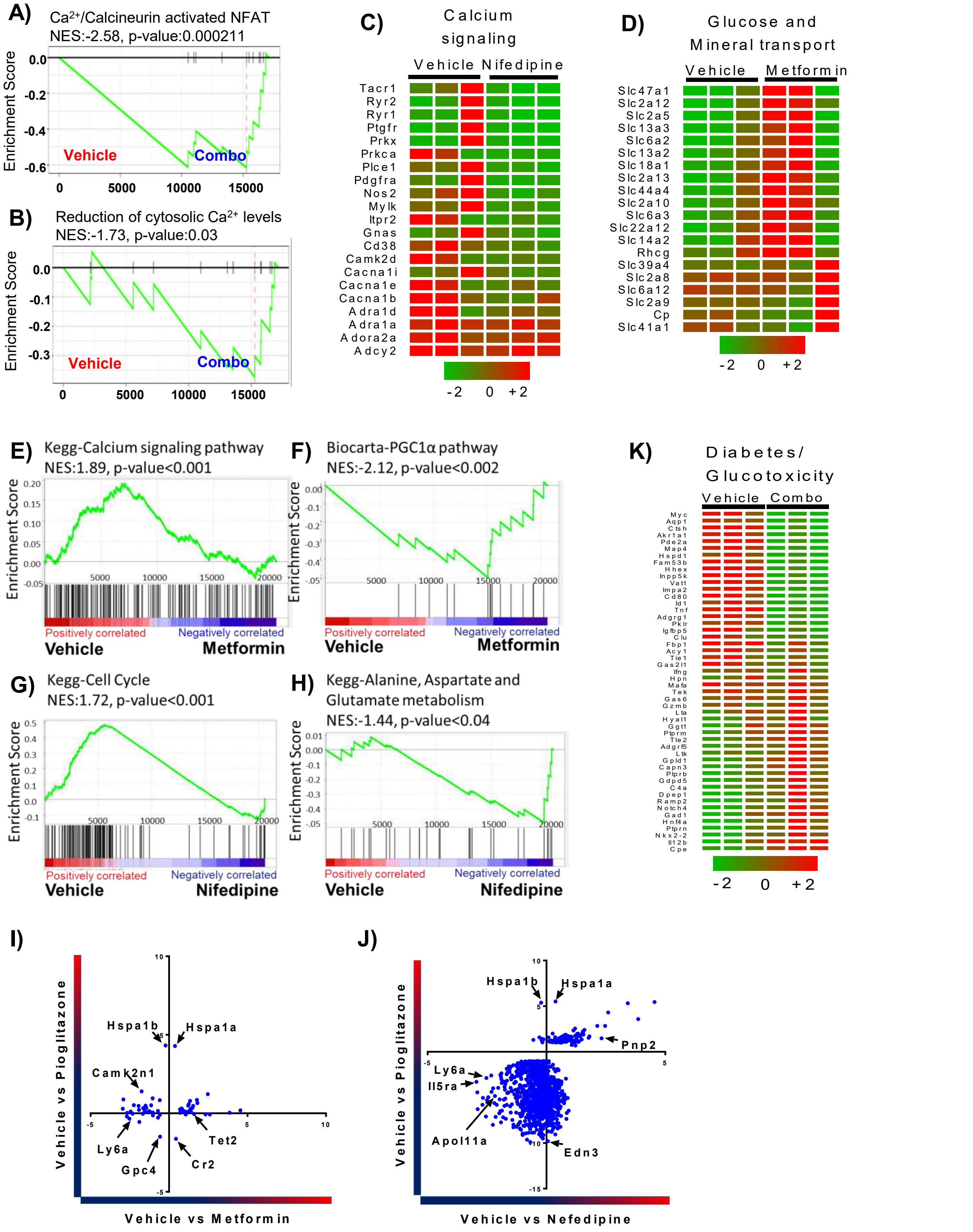

Supplemental Figure 14

Supplemental Figure 14: Effect of metformin, nifedipine, MCC950 and anakinra treatment on gene expression

(A, B) GSEA plots of gene sets related to Ca2+/calcineurin activated NFAT pathway **(A)** and reduction of cytosolic Ca^{2+} levels pathway **(B)** was repressed in combination of drug treated *Ob/Ob* mice bearing *Tet2*$^{-/-}$ cells compared to vehicle treatment. NES, normalized enrichment score.

(C, D) Heatmaps of differentially expressed genes in the calcium signaling pathway (**C**), glucose and mineral transport signaling pathway (**D**) after 30 days of the indicated drug treatment.

(E-H) GSEA plots of gene sets related to calcium signaling (**E**), PGC1α pathway (**F**), cell cycle (**G**) and alanine, aspartate, and glutamate metabolism signaling pathway (**H**) after 30 days of the indicated drug treatment. NES, normalized enrichment score; KEGG, Kyoto Encyclopedia of Genes and Genomes.

(I, J) SPA analysis of differential gene expression in BM cells was assessed by RNA-seq from indicated drug treatment groups.

(K) Heatmap of differentially expressed genes of diabetes/glucotoxicity pathway after 30 days of the indicated drug treatment.
